# Unified bilayer membranes with mechanically reinforced interface for stage-adaptive bone regeneration

**DOI:** 10.1038/s41467-026-74932-x

**Published:** 2026-06-30

**Authors:** Yuhan Du, Yangyi Nie, Zhiheng Luo, Junjie Deng, Zili Xu, Yuyang Zhang, Chenyu Gao, Yujie Liu, Xiangcheng Lei, Jing Long, Cairong Li, Zhenyu Yao, Wei Zhang, Ling Qin, Yuanchi Zhang, Yuxiao Lai

**Affiliations:** 1 Centre for Translational Medicine Research and Development, Shenzhen Institutes of Advanced Technology, Chinese Academy of Sciences, Shenzhen, China; 2 University of Chinese Academy of Sciences, Beijing, China; 3 Tianjin Hospital, Tianjin University, Tianjin, China; 4 The Brain Cognition and Brain Disease institute of Shenzhen Institutes of Advanced Technology, Chinese Academy of Sciences, Shenzhen, China; 5 Faculty of Health Sciences, University of Macau, Macau, China; 6 Department of Orthopaedics and Traumatology, The Chinese University of Hong Kong, Hong Kong, China; 7 Key Laboratory of Biomedical Imaging Science and System, Chinese Academy of Sciences, Shenzhen, China; 8 Guangdong Engineering Laboratory of Biomaterials Additive Manufacturing, Shenzhen, China; 9 National Innovation Center for Advanced Medical Devices, Shenzhen, China

**Keywords:** Fracture repair, Biomedical materials, Biomedical engineering, Biomedical materials

## Abstract

Guided bone regeneration using double-layered membranes is often hindered by poor interfacial integration, insufficient stability, and limited adaptability to regulate the complex healing process. Herein, we develop a unified bilayer membrane featuring a reinforced dual-crosslinking interface between barrier and porous layers through a homogeneous isomeric in-situ fabrication strategy. Its superior mechanical strength (~156 MPa in dry state and ~8.6 MPa in wet state) ensures the long-term degradation stability and bioactivity in vivo. Combined with sequentially released bioactive components, we show that the unified bilayer membrane regulates antibacterial functions, immunomodulation, neurovascularization, and osteogenesis, thereby enabling stage-adaptive bone regeneration. We test in a *rat* critical-sized cranial defect model to demonstrate that the membrane significantly outperforms the clinical collagen membrane, achieving ~2.7-fold greater bone volume regeneration after 8 weeks. This work further elucidates the spatiotemporal osteogenic mechanisms of the unified bilayer membrane, which constructs a well-integrated regenerative microenvironment and promotes a tightly interconnected neurovascular-osteogenic communication network during stage-adaptive bone repair.

## Abstract (web summary)

Guided bone regeneration membranes suffer from insufficient mechanical stability and bioactive adaptability. Here, the authors report a unified bilayer membrane with reinforced interface for stage-adaptive bone regeneration and investigate its spatiotemporal osteogenic mechanisms.

## Introduction

Delayed bone healing and dysregulated tissue regeneration still remain the most critical biological challenges in clinical bone repair, significantly compromising postoperative recovery and the quality of bone reconstruction^1–3^. Bone repair proceeds as a staged cascade comprising early infection control, immune-microenvironment remodeling, cell recruitment/migration, and neurovascular/bone regeneration, where disruption at any stage compromises downstream stages^4,5^. Specifically, after implantation of the device, bacterial infection and the host inflammatory response often predominate, followed by endothelial dysfunction and aberrant angiogenesis due to ensuing chronic inflammation. Then the neural/vascular sprouting and neurovascular coupling are disturbed, thereby further degrading the local microenvironment and ultimately limiting subsequent regenerative capacity^6–9^. However, current implants lack adaptive regulation capacity for above repairing stages. Moreover, the mechanical incompatibility of these implants predisposes to early degradation or structural instability under complex pathological microenvironments, resulting in device deterioration and functional failure for unsatisfactory bone regeneration^10^. Therefore, developing stage-adaptive implants with reinforced mechanical properties to deliver cascade-wide regulation is essential for effective bone regeneration and functional reconstruction.

Native bilayer periosteum offers a particularly informative model for unifying mechanical integrity with staged bioactive delivery, which tightly envelopes the bone surface to provide mechanical protection and dynamic biological regulation during bone healing process^11,12^. The outer fibrous layer of a periosteum acts as a physical barrier and anchoring interface for tendons, effectively preventing external mechanical stress, bacterial invasion, and soft tissue infiltration. The inner cambium layer of a periosteum is rich in osteoprogenitor cells, neurovascular networks, and regenerative signaling molecules, which collaboratively orchestrate various stages in bone regeneration^13^. Clinically, natural periosteum is scarce due to surgical removal or limited donor sources. Even when obtained, its biological functions are compromised and unstable by individual variability^14^. Therefore, a series of bioactive membranes has been developed to simulate properties of native periosteum for accelerating bone healing^15,16^. Recently, these bioactive membranes with double-layer structures have been fabricated by advanced technologies, such as electrospinning and 3D printing, but most layered structures of the membranes are still confronted with limited interfacial integration and easy to delaminate issues^17–19^. In addition, despite that these membranes achieve certain functions of the native periosteum e. g. barrier protection and angiogenic activity, most implants cannot adaptively regulate the bone regeneration according to biological requirements at various healing stages.

**Fig. 1:**
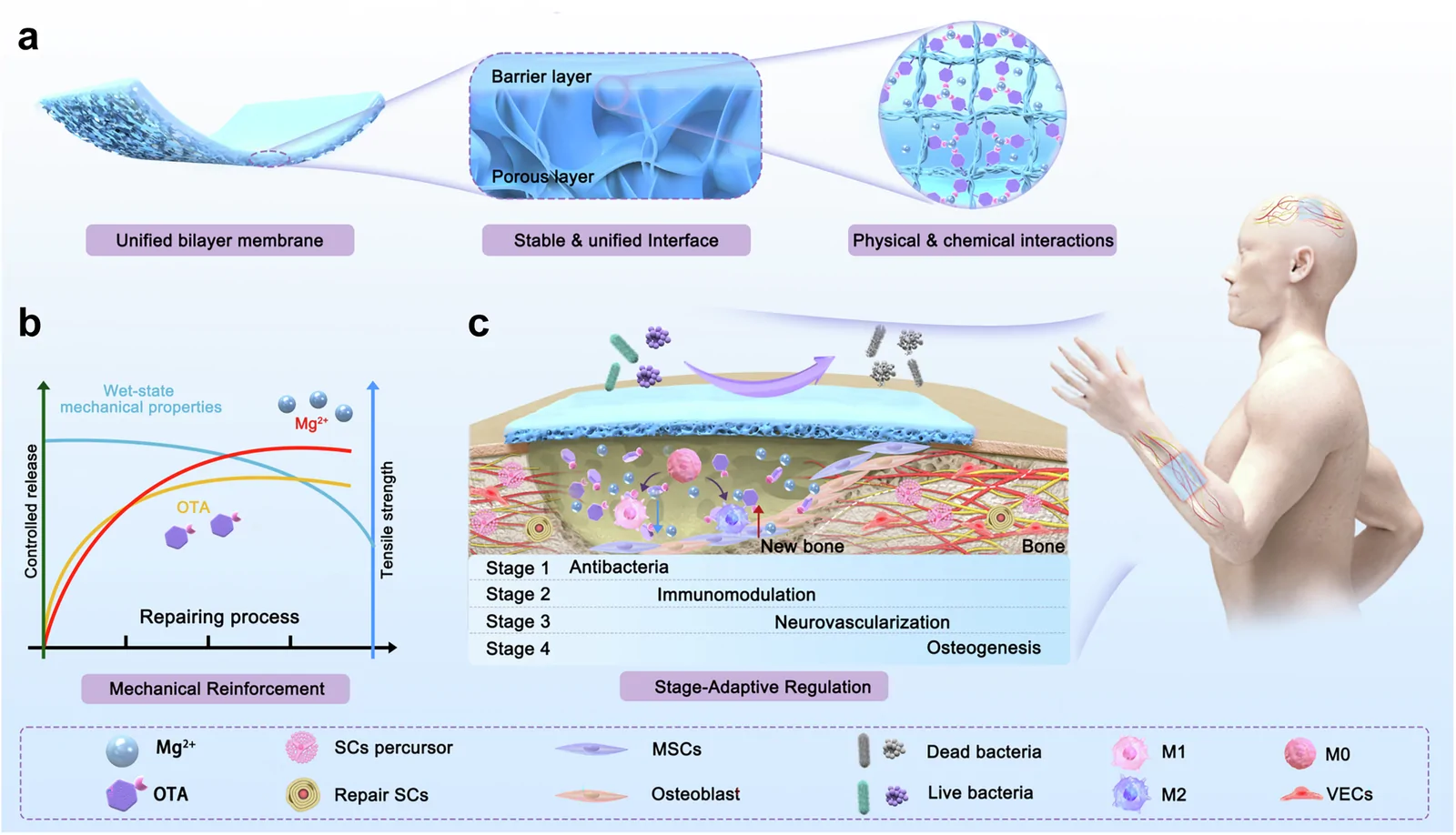
Schematic illustration of unified bilayer membranes for stage-adaptively regulated bone generation. **a** The CSO_1_M_3_ membranes were constructed with a stable and unified interface between the barrier layer and porous layer via inherent physical and chemical interactions. **b** The membranes exhibited a controlled release profile for bioactive OTA and Mg^2+^, and concurrently maintained structural stability under hydrated conditions during repairing process. **c** Due to the mechanical reinforcement and sustained release of OTA and Mg^2+^, the membranes enabled stage-adaptive regulation including antibacteria (Stage I), immunomodulation (Stage II), neurovascularization (Stage III), and osteogenesis (Stage IV), which ultimately enhanced bone regeneration at various defective sites.

Herein, we propose unified bilayer composite membranes containing chitosan (CS), oxidized tannic acid (OTA) and magnesium oxide (MgO) nanoparticles to achieve reinforced interface and stage-adaptive regulation for improving ultimate bone regeneration (Fig. 1a). The structural backbone of the composite membranes was constructed via covalent Schiff-base linkages between the amino groups of CS and the quinone groups of oxidized OTA (Suppl. Fig. 1). This primary network was further strengthened through phenolic coordination systems formed by the phenolic hydroxyl groups of OTA and MgO nanoparticles. To achieve high mechanical properties and mimic the bilayer architecture of native periosteum, we utilized a homogeneous isomeric in-situ forming method to unify the two disparate layers of the composite membranes. This fabrication strategy brings physical stacking with chemical crosslinking interaction at the interface between the two layers of the composite membranes, which can eliminate interfacial discontinuities between layers to enhance structural uniformity and mechanical reliability of the membranes for supporting the entire bone repair process. The barrier layer of the membranes, initially formed by solvent evaporation, serves to provide long-term mechanical support, prevent bacteria and soft tissue infiltration, as well as maintain defect space integrity (Fig. 1b). The porous layer, formed on the barrier layer by freeze-drying the composite solution, confers stage-adaptive regulation across the bone healing process via the sequential and stable release of OTA and Mg^2+^ (Fig. 1c). As a proof of concept, we first prepared a series of unified bilayer membranes including the pristine CS membranes, CS incorporated with OTA (CSO_1_, CSO_2_, CSO_3_, respectively) membranes, CSO_1_ incorporated with MgO nanoparticles (CSO_1_M_1_ (1 wt% MgO), CSO_1_M_2_ (2 wt% MgO), CSO_1_M_3_ (3 wt% MgO), CSO_1_M_4_ (4 wt% MgO), CSO_1_M_8_ (8 wt% MgO), respectively) membranes. Then we conducted a series of in vitro studies to investigate the structures, mechanical performance, degradation, releasing ability and biocompatibility of the membranes. Further, we comprehensively evaluated stage-adaptive regulation of the unified bilayer membranes on repairing process, including antibacteria (Stage I), immunomodulation (Stage II), neovascularization (Stage III) and osteogenesis (Stage IV) for ultimate bone regeneration.

## Results

### Characterization of the unified bilayer membranes

**Fig. 2:**
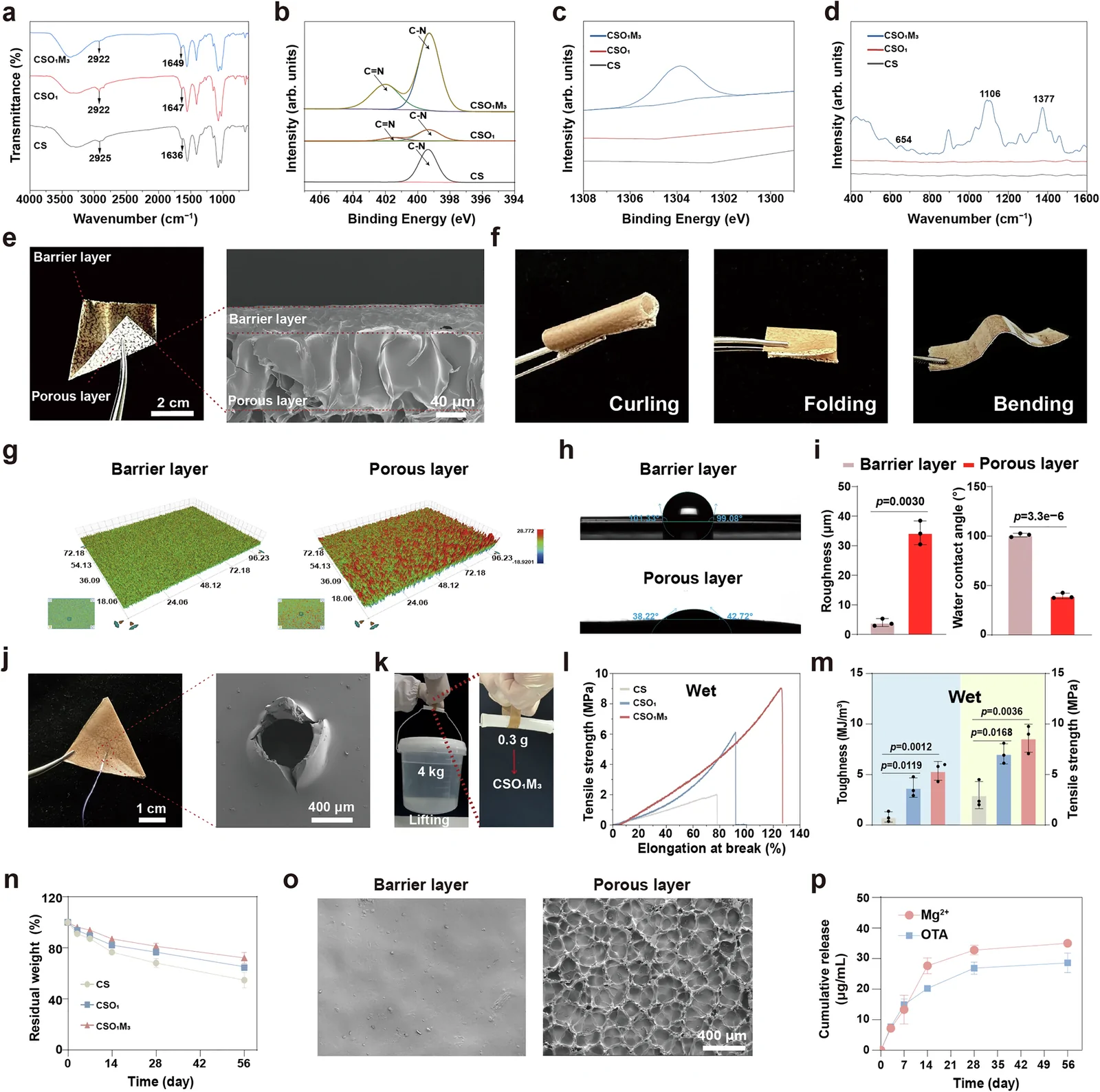
Characterization of the unified bilayer membranes. **a** FTIR spectra of various membranes. CS: pristine CS. CSO_1_: CS incorporated with 1 wt% OTA. CSO_1_M_3_: CSO_1_ with 3 wt% MgO. **b** Binding energies of nitrogen (N 1*s*) in the XPS spectra of membranes. **c** Binding energies of Mg (Mg 1*s*) in the XPS spectra of membranes. **d** Raman spectra of the membranes. **e** Macroscopic photographs and cross-sectional SEM images of the CSO_1_M_3_ membranes. The images were repeated at least three times with consistent results. **f** Images of the CSO_1_M_3_ membranes with various deformations. **g**–**i** Heterogeneous morphologies of barrier layer and porous layer including surface roughness analysis (**g**), water contact angle measurements (**h**) and corresponding data statistics (**i**). **j** Surgical suture puncture tests of the CSO_1_M_3_ membranes and amplified SEM images of the puncture sites. The images were repeated at least three times with consistent results. **k** Lifting tests with a 4 kg load using the CSO_1_M_3_ membranes, the red arrow indicates the CSO_1_M_3_ membranes. **l**, **m** Representative tensile stress-strain curves (**l**) and derived tensile strength and toughness (**m**) of various composite membranes under hydrated conditions. **n** Degradation in vitro of the composite membranes in 56 days. **o** SEM images of the two layers of the CSO_1_M_3_ membranes after 56-days in vitro degradation. The images were repeated at least three times with consistent results. **p** Cumulative magnesium ions and OTA release curve of the CSO_1_M_3_ membranes in 56-days in vitro degradation. Values in (**i**, **m**, **n**), and (**p**) represent the mean and standard deviation (*n*  =  3 independent samples per group). Statistical analyses were performed using two-tailed Student’s *t*-test (**i**) and one-way ANOVA followed by Tukey’s post hoc test (**m**). *p* < 0.05 was considered statistically significant differences between the compared groups. Source data are provided as a Source Data file.

Inspired by natural periosteum, we designed and engineered composite membranes with unified barrier and porous layers through dual crosslinking network. The reinforced interface of the membrane was expected to significantly enhance mechanical strength and degradable stability for long-term bone healing process. Structures of the membranes were first characterized. The appearance of MgO nanoparticles and OTA was confirmed via scanning electron microscopy (SEM) images and Fourier transform infrared spectroscopy (FTIR) spectra (Suppl. Fig. 2). FTIR results showed the shifts of characteristic bands of carbonyl groups from 1636 cm^−1^ (CS) to 1647 cm^−1^ and 1649 cm^−1^ (CSO_1_ and CSO_1_M_3_, respectively), which indicated amide bonds formation through Schiff-base reactions^20^. X-ray photoelectron spectroscopy (XPS) confirmed the formation of Schiff-base linkages in the CSO_1_ and CSO_1_M_3_ with characteristic C = N peaks at 401.5 eV and 402.0 eV in the spectra, respectively (Fig. 2b)^21^. Additionally, the peak at 1303.8 eV in the CSO_1_M_3_ spectrum indicated successful incorporation of MgO nanoparticles (Fig. 2c). Raman spectra further observed peaks at 654, 1106, and 1377 cm^−1^, corresponding to the Mg^2+^-catechol coordination, demonstrating the formation of metal-phenolic networks within the composite membranes (Fig. 2d)^22^. Notably, cross-sectional SEM images of the CSO_1_M_3_ membranes proved a continuous bilayer in well-integrated unified bulk without visible delamination or interfacial discontinuity, highlighting the effects of the integrated fabrication strategy on promoting interlayer cohesion and structural integrity (Fig. 2e). Due to the special unified bilayer structures, the membranes could be curled, folded, and bent without delaminating, cracking or structural compromise (Fig. 2f). Such flexibility endowed convenient surgical operation and satisfactory conformity to irregular bone defects. In addition, the barrier layer and porous layer of the CSO_1_M_3_ membranes were characterized (Fig. 2g–i and Suppl. Fig. 3). The barrier layer had a surface roughness (Ra) of ~4.0 µm and a water contact angle of ~101° to minimize bacterial adhesion, while the porous layer had a Ra of ~34.4 µm and a water contact angle of ~40° to enhance fluid infiltration and bioactive molecules delivery^23^. Energy dispersive spectrometry (EDS) images confirmed a uniform distribution of Mg^2+^ across both layers. This asymmetric bilayer in unified bulk realized a rational imitation of the structures and functions of the native periosteum.

Mechanical performance of the membranes was important for providing robust support to defective bones. The dosage effects of varying concentrations of OTA (1–3 wt%) and MgO nanoparticles (1–8 wt%) on the mechanical properties of the membranes were first explored (Suppl. Fig. 4). The results showed that introducing 1 wt% OTA and 3 wt% MgO could significantly enhance the ultimate strength (~156 MPa) and toughness (~72 MJ m^−3^) of the composite membranes. Therefore, the CSO_1_M_3_ membranes were used to conduct subsequent experiments. To validate qualitatively the mechanical properties of the CSO_1_M_3_ membranes under clinically relevant conditions, surgical suture puncture tests were performed (Fig. 2j). No secondary crack propagation was observed around the puncture sites, indicating good tear resistance of the CSO_1_M_3_ membranes. In addition, we used the CSO_1_M_3_ membranes to lift a 4 kg load, highlighting their robust load-bearing capability (Fig. 2k, Suppl. Movie 1). Considering the physiological environments in clinical applications, the swelling ratio and mechanical performance of the membranes were quantitatively evaluated under hydrated conditions. While the pristine CS membranes showed an excessive swelling ratio of ~638% in 24 h, the CSO_1_ and CSO_1_M_3_ membranes showed significantly reduced swelling ratios of ~231% and ~165%, respectively (Suppl. Fig. 5). This might be attributed to the dual crosslinking network that restricted polymer chains mobility and water diffusion^24,25^. The uniformly distributed MgO nanoparticles further functioned as nanoscale rigid anchors, impeding water penetration and mitigating expansion^25^. As for the mechanical properties at wet state, the CSO_1_M_3_ membranes had an enhanced strength of ~8.6 MPa and a toughness of ~5.4 MJ m^−3^, which were significantly greater than those of the CS membranes (~3.0 MPa and 0.8 MJ m^−3^), CSO_1_ membranes (~7.1 MPa and 3.7 MJ m^−3^), and native periosteum (~4.0 MPa) (Fig. 2l, m). Moreover, the CSO_1_M_3_ membranes displayed an elongation at break of ~135%, indicating favorable stretchability and mechanical stability of the CSO_1_M_3_ membranes under physiological conditions. The unified bilayer membranes exhibited superior comprehensive mechanical properties among the leading double-layer membrane materials reported to date, which could provide a stable environment for bone regeneration (Suppl. Fig. 6).

Long-term degradation behaviors of the membranes in vitro were further investigated. After being placed in phosphate-buffered saline (PBS) buffer containing lysozyme for 56 days, the CS membranes retained ~55% of their initial weights, indicating rapid degradation due to enzymatic cleavage and hydration-induced polymer relaxation (Fig. 2n). The CSO_1_ and CSO_1_M_3_ membranes showed improved stability with respective residual weights of ~65% and ~73% due to the dual-crosslinked polymer networks in the membranes. Macroscopic observations confirmed the shape stability of the CSO_1_M_3_ membranes on the *rat* skull after 8 weeks of degradation, with a corresponding residual tensile strength of ~300 kPa (Suppl. Fig. 7). This mechanical performance could ensure stable tissue suturing for structural support during bone repair process. SEM images showed that the barrier and porous layers of the CSO_1_M_3_ membranes could maintain their structural integrity without any signs of delamination or structural fragmentation after 56 days (Fig. 2o). Moreover, cross-sectional observations of degradation further confirmed that the bilayer interface had no obvious separation or disintegration, highlighting the unified stability of the composite membranes for long-term employment in vivo (Suppl. Fig. 8). The long-term stability of the unified bilayer membranes could be attributed to the reinforced dual-crosslinking network (Schiff-base and phenolic-Mg^2+^ coordination) that limited hydrolytic and enzymatic access to the matrix^26,27^. Taken together with the residual weight curve, we extrapolated that the complete resorption would happen in ~6–8 months, which could meet the clinically required degradation period for practical use. In addition, the pH values of the degradation medium for all groups remained within a stable physiological range (7.2–7.4), providing a favorable and consistent microenvironment to support new bone growth (Suppl. Fig. 9).

Correspondingly, the release of OTA and Mg^2+^ ions from the CSO_1_ and CSO_1_M_3_ membranes was quantitatively measured (Fig. 2p). During the initial 14 days, rapid release of Mg^2+^ ions and OTA occurred predominantly with degradation of the membranes. This burst phase effectively delivered immediate anti-inflammatory and immunomodulatory components, facilitating macrophage polarization toward the pro-regenerative (M2) phenotype, crucial for initiating the healing process^28,29^. After 14 days, the release of these bioactive components transitioned into a stable phase, mediated by the controlled erosion and gradual degradation of the membranes. During this phase, the released OTA provided sustained antioxidative protection and alleviated oxidative stress, while effectively eliminating bacterial invasion. Simultaneously, the released Mg^2+^ ions could promote neurovascular regeneration as well as osteoblast differentiation and mineralization^30,31^. The unified bilayer CSO_1_M_3_ membranes integrated robust mechanical properties with biological functionality, contributing to adaptively regulate the distinct physiological stages of inflammation resolution and tissue regeneration.

### Barrier and antibacterial effects of unified bilayer membranes

**Fig. 3:**
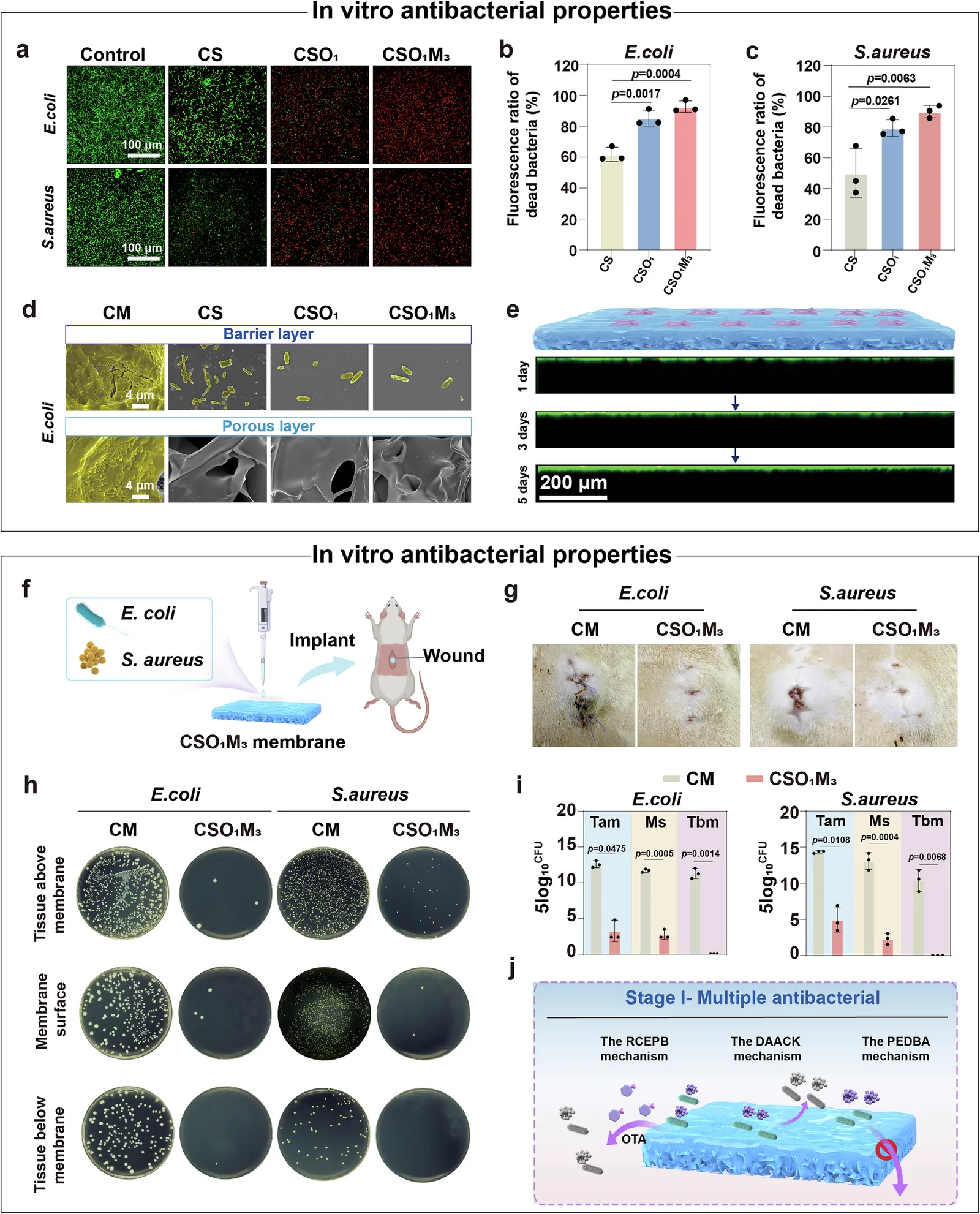
Barrier and antibacterial properties of the unified bilayer membranes. **a**–**c** Live/dead staining results (**a**) and quantitative analysis of bacterial death rates including *E. coli* (**b**) and *S. aureus* (**c**) after 3-days co-culture with various composite membranes. **d** SEM images of the barrier layers and the porous layers in various groups after *E. coli* penetration for 7 days. The images were repeated at least three times with consistent results. **e** Vertical growth (z-axis) of L929 fibroblasts on the barrier layer of the CSO_1_M_3_ membranes for incubating 1, 3 and 5 days. **f** Schematic of a *rat* subcutaneous infection model. Bacterial suspension was carefully dripped onto the barrier layer of the membranes, followed by implanting at subcutaneous sites of the *rat* model for 7 days. **g** Macroscopic evaluation of the surgical sites after implanting the membranes for 7 days. **h**, **i** Representative images (**h**) and quantitative analysis (**i**) of bacterial burden from three areas: tissue above membranes (Tam), membrane surface (Ms), and tissue below membranes (Tbm). **j** Schematic of antibacterial mechanisms of the unified bilayer membranes. The RCEPB mechanism: Rapid Chemical Elimination of Planktonic Bacteria mechanism. The DAACK mechanism: Dynamic Anti-Adhesion and Contact-Killing mechanism. The PEDBA mechanism: Physical Exclusion by Dense Barrier Layer mechanism. Values in (**b**, **c**, **i**) represent the mean and standard deviation (*n*  =  3 independent samples per group). Statistical analyses were performed using one-way ANOVA followed by Tukey’s post hoc test (**b**, **c**) and two-tailed Student’s *t*-test (**i**). *p* < 0.05 was considered statistically significant differences between the compared groups. Source data are provided as a Source Data file.

At the initial stage of bone repairing process, orthopedic implants are susceptible to infectious complications, mainly caused by bacterial invasion from external sources^32^. Therefore, we designed the bilayer structure and the components of the membranes for achieving multiple antibacterial effects once implanted (Stage I)^33–35^. In bacteria co-culture tests, the CSO_1_M_3_ membranes were demonstrated with significantly higher 24-hour antibacterial rates against Staphylococcus aureus (*S. aureus*) (~74%) and Escherichia coli (*E. coli*) (~70%) compared to those of the CS membranes (~46% and ~40%), representing a ~ 1.6-fold improvement (Suppl. Fig. 10). Live/dead staining images after three days of the co-culture tests confirmed the antibacterial effects of the CSO_1_M_3_ membranes, showing ~90% of death rate for *S. aureus* and ~93% of death rate for *E. coli* (Fig. 3a–c). The agar plate images also proved obviously reduced colony density of *S. aureus* and *E. coli* in the CSO_1_M_3_ group (Suppl. Fig. 11). To simulate the inward penetration and invasion of bacteria through implants, a transmembrane penetration model was established. The sample was fixed within a well plate, then bacteria were applied to the outer surface of the sample and then incubated for 7 days. Commercial collagen membranes (CM) were used as the control group, which had also bilayer structures (Suppl. Fig. 12). SEM images showed dense biofilm formation by *S. aureus* and *E. coli* on the CM group’s barrier layer, with bacterial penetration into the porous layer (Fig. 3d, Suppl. Fig. 13). In contrast, CS-based membranes, especially the CSO_1_ and CSO_1_M_3_ membranes showed only a few distorted bacteria attached to the barrier layer surface, effectively preventing biofilm formation. Moreover, there was no bacterial penetration into the porous layer of these CS-based membranes, confirming their superior anti-adhesion and barrier properties. Besides, the unified bilayer membranes could effectively prevent fibroblast cells (e.g., L929 cells) from penetrating through the barrier layer for prohibiting connective tissue from invading into the damaged sites (Fig. 3e). Meanwhile, the membranes had satisfactory biocompatibility to support cell adhesion and proliferation (Suppl. Fig. 14).

In vivo antibacterial studies using a *rat* subcutaneous infection model were further performed (Fig. 3f). After subcutaneous implantation of the CSO_1_M_3_ and CM membranes, a bacterial suspension was carefully dripped onto each surface of their barrier layers. The surgical incisions were subsequently sutured, and the animals were monitored for 7 days to evaluate the antibacterial performance of the membranes. The results indicated no signs of infection were observed in the CSO_1_M_3_ group, whereas the CM group exhibited swelling incisions (Fig. 3g). Plate count assays demonstrated that the CSO_1_M_3_ membranes had robust antibacterial efficacy in all tissue layers (Fig. 3h, i). Specifically, *E. coli* and *S. aureus* colony counts in the CSO_1_M_3_ group were respectively reduced by ~74% and ~65% in the tissue above membranes (Tam) areas compared to the CM group. In tissue below membranes (Tbm) areas where bacterial infiltration poses the greatest threat to regeneration, the CSO_1_M_3_ group achieved ~100% antibacterial exclusion for *E. coli* and *S. aureus*. Hematoxylin and eosin (H&E) staining images of the skin tissue sections indicated severe inflammatory infiltration in the CM group, while the CSO_1_M_3_ group exhibited significantly reduced inflammation and preserved tissue architecture (Suppl. Fig. 15). These results confirmed that the unified bilayer membranes had good antibacterial functions, which was the prerequisite for effective bone generation.

Based on above results, the antibacterial mechanisms of the CSO_1_M_3_ membranes were summarized in Fig. 3j. The first one was the Rapid Chemical Elimination of Planktonic Bacteria (RCEPB) mechanism: Released OTA from the membranes had polyphenolic structures that could interact non-specifically with bacterial membrane proteins and lipids, followed by disrupting membrane integrity and quickly eliminating free-floating (planktonic) bacteria during the initial phase of contamination. The second one was the Dynamic Anti-Adhesion and Contact-Killing mechanism (DAACK): The barrier layer of the membranes was engineered to be highly smooth and hydrophobic, effectively reducing bacterial adhesion and lowering infection risk. Even few bacteria adhered, the positive charges of the CS matrix enhanced antibacterial effects by disrupting bacterial cell membranes. Additionally, antibacterial agents e.g., OTA continuously released from the surface induced further bactericidal reactions, thereby preventing colonization and biofilm development^36^. The third one was Physical Exclusion by Dense Barrier Layer (PEDBA) mechanism: A compact barrier layer of the membranes provided physical protection by blocking microbial penetration into deeper tissues. This spatial insulation was essential for preserving a sterile interface between soft tissues and bone defects, particularly during the critical early stages following implantation^37^. Among these mechanisms, the RCEPB mechanism might be the dominant one because it provided rapid and effective antibacterial defense at the initial stage of bacterial contamination. The DAACK mechanism then eliminated bacteria that reached the membrane’s surface, preventing the formation of mature biofilms through the smooth surface and positive charges of the CS matrix. Furthermore, the PEDBA mechanism could provide long-term barrier function to block bacterial penetration into the defective site. Integrating structural stability with controlled degradability, the unified bilayer membrane provided multiple antibacterial functions to achieve a sterile local microenvironment.

### Immunomodulation of the unified bilayer membranes

**Fig. 4:**
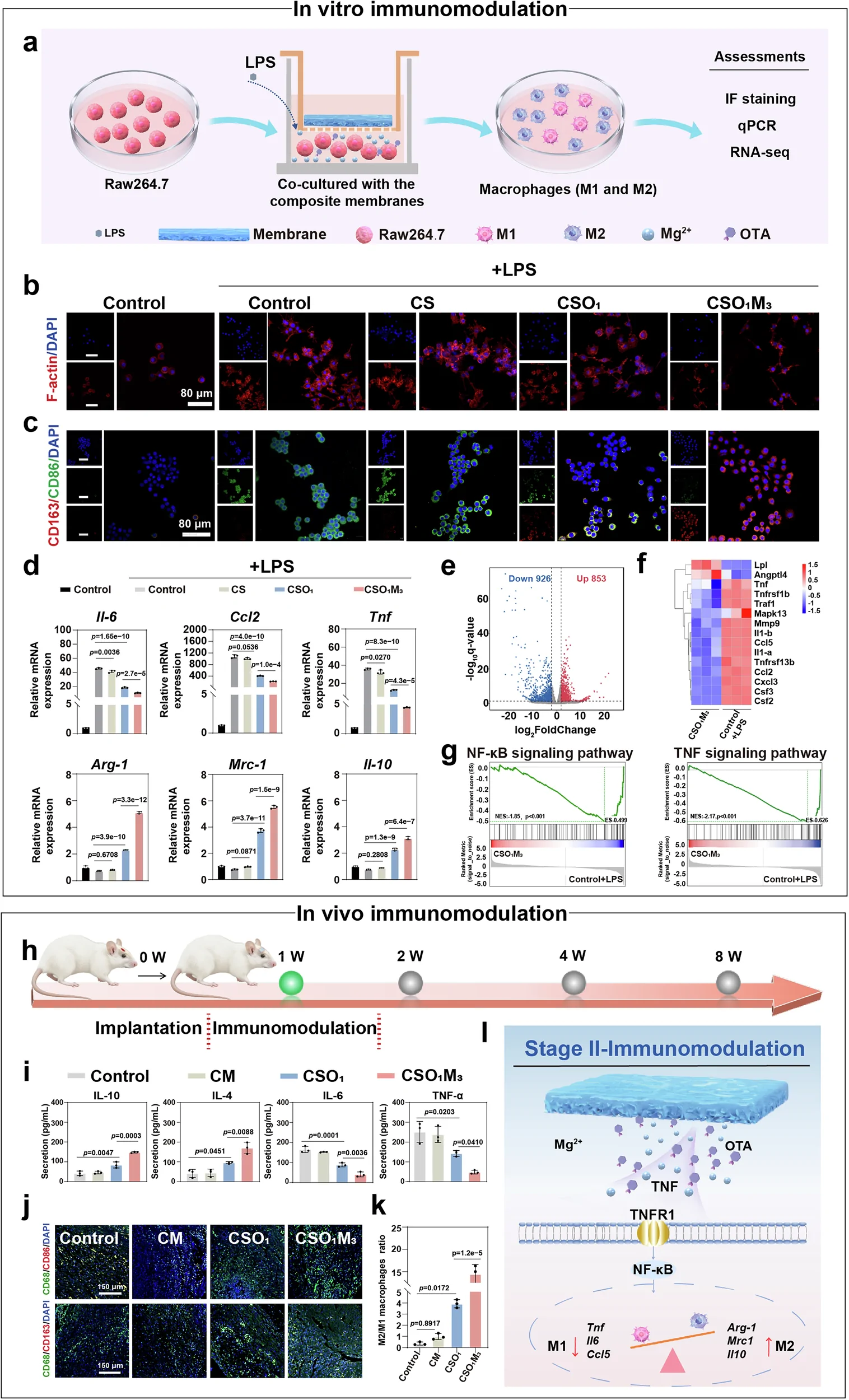
In vitro and in vivo immunomodulation of the unified bilayer membranes. **a** Schematic of in vitro experimental design on the anti-inflammatory effects of composite membranes. **b** Representative IF images of RAW 264.7 cells co-cultured with the membranes under no or LPS stimulation after 48 h. Red: F-actin. Blue: nuclei. **c** Representative IF staining of CD86 (M1 marker, green) and CD163 (M2 marker, red) images of RAW 264.7 cells co-cultured with the membranes under no or LPS stimulation after 48 h. **d** qPCR analysis of M1-related genes (*Il6, Ccl2, Tnf*) and M2-related genes *(Arg1, Mrc1, Il10*) expression. **e** Volcano plot of differentially expressed genes obtained from RNA-seq of LPS-stimulated RAW 264.7 cells with or without the CSO_1_M_3_ membranes treatment. **f** Heatmap showing expression levels of immune-related genes derived from RNA-seq data. **g** GSEA analysis of the NF-κB and TNF-α signaling pathways of the RAW 264.7 cells with the CSO_1_M_3_ membranes treatment. Statistical analysis was performed using a one-sided permutation test without adjustment for multiple comparisons. NES indicates the normalized enrichment score. *p* < 0.05 was considered statistically significant differences between the compared groups. **h** Schematic of the in vivo experimental design using a *rat* cranial critical-sized defect model to evaluate local immune responses after implantation for 1 week. **i** The levels of IL-6, TNF-α, IL-10 and IL-4 cytokines in the serum of the *rat* model measured by the Luminex assay after implantation for 1 week. **j** Representative IF staining of CD68 (pan-macrophage marker, green), CD86 (M1 marker, red), and CD163 (M2 marker, red) images of tissues from the defective areas to evaluate macrophage polarization in vivo. **k** Quantitative analysis of CD86⁺ (M1 phenotype) and CD163⁺ (M2 phenotype) macrophages based on the IF staining images in (**j**), with calculation of the M1/M2 ratios among various groups. **l** Schematic of the immunomodulation mechanisms by the CSO_1_M_3_ membranes. Values in (**d**), (**i**), and (**k**) represent the mean and standard deviation (*n*  =  3 indepe*n*dent samples per group). Statistical analyses were performed using one-way ANOVA followed by Tukey’s post hoc test. *p* < 0.05 was considered statistically significant differences between the compared groups. Source data are provided as a Source Data file.

Inflammation is of great significance for effective bone regeneration. One week after the implantation, immune modulation (Stage II) was indispensable for further neurovascular and osteogenic progression. Among various immunoregulatory mechanisms, the transition of macrophages from the pro-inflammatory M1 phenotype to the pro-regenerative M2 phenotype is widely recognized as a critical process that mitigates inflammation and supports bone regeneration^38^. In vitro studies were first conducted to investigate the anti-inflammatory effects of composite membranes (Fig. 4a). The cytocompatibility of the composite membranes co-cultured with RAW264.7 macrophages was first proved by the Cell Counting Kit-8 (CCK-8) assay, which the CSO_1_M_3_ membranes were demonstrated to notably promote macrophages proliferation (Suppl. Fig. 16). Under lipopolysaccharide (LPS) stimulation, macrophages tended to polarize into M1 phenotype as indicated by the small and flattened shape in the control groups. In contrast, macrophages co-cultured with the CSO_1_M_3_ membranes predominantly adopted an elongated and spindle-shaped morphology, proving the polarization to M2 phenotype (Fig. 4b). To validate this morphological evidence, immunofluorescence (IF) staining and quantitative analysis of CD86 (M1 marker) and CD163 (M2 marker) were performed. Compared to the control and CS groups with LPS, the CSO_1_ and CSO_1_M_3_ groups significantly downregulated CD86 expression while upregulating CD163 expression, indicating high promotion of M2 macrophage polarization. Particularly, the CSO_1_M_3_ membranes could further reduce CD86 expression by ~56% and increasing CD163 expression by ~104% compared to those of the CSO_1_ membranes (Fig. 4c and Suppl. Fig. 17). OTA could create a favorable microenvironment by scavenging localized reactive oxygen species, which further improved the immunomodulatory efficiency of Mg^2+^. Therefore, this enhanced M2 polarization was attributed to the synergistic immunomodulatory effects of OTA and Mg^2+^ released from the CSO_1_M_3_ matrix^39,40^. Consistent with these phenotypic changes, quantitative Polymerase Chain Reaction (qPCR) analysis showed marked downregulation of M1-related genes (*Il6*, *Ccl2*, *Tnf*) and upregulation of M2-related genes (*Arg1*, *Mrc1*, *Il10*) of the macrophages co-cultured with the CSO_1_M_3_ membranes, showing the strongest regulatory capacity among the CS-based membranes (Fig. 4d). Enzyme-linked immunosorbent assay (ELISA) results further confirmed the decreased secretion of pro-inflammatory cytokines IL-6 and TNF-α in the CSO1 and CSO_1_M_3_ groups, particularly in the latter (Suppl. Fig. 18). To further elucidate the underlying molecular mechanisms, RNA sequencing (RNA-seq) was conducted using LPS stimulated RAW264.7 macrophages cultured with the CSO_1_M_3_ membranes for 48 hours. Differential expression analysis revealed 926 upregulated and 853 downregulated genes (|log₂FC | > 1, adjusted *P* < 0.05) relative to the LPS control group (Fig. 4e). Gene Ontology (GO) enrichment analysis showed suppression of inflammatory and innate immune processes, while upregulated enrichment of IL-4-related terms (Suppl. Figs. 19, 20)^41^. Kyoto Encyclopedia of Genes and Genomes (KEGG) pathway analysis further revealed downregulation of pro-inflammatory pathways such as TNF, NF-κB, and cytokine-cytokine receptor interaction, alongside upregulation of the PPAR signaling pathway, which was known to drive M2 macrophages polarization^42^. A heatmap of representative immune-related genes based on Fragments Per Kilobase of exon per Million mapped reads (FPKM) values highlighted the reduced expression of inflammatory mediators including *Mmp9*, *Tnf*, *Il1b*, and *Ccl5* (Fig. 4f). Additionally, Gene Set Enrichment Analysis (GSEA) showed significantly negative enrichment of NF-κB and TNF signaling pathways in the CSO_1_M_3_ group, further supporting the anti-inflammatory and immunoregulatory properties of the membranes (Fig. 4g). Collectively, these in vitro findings demonstrated that the CSO_1_M_3_ membranes effectively regulated macrophages by suppressing pro-inflammatory signaling and providing a regeneration microenvironment for new tissue growth.

In vivo immunomodulation studies were performed to investigate the stage-adaptive regulation of the composite membranes for ultimate bone generation via a *rat* cranial critical-sized defect model (~5 diameter) (Fig. 4h). After 1 week implantation, *rat* serum and cranial defects were collected for Luminex-based cytokine assay and IF staining analysis, respectively. Compared to the untreated control group, all composite membrane groups exhibited reduced levels of pro-inflammatory cytokines (IL-6, TNF-α, IL-1α, IL-1β) and increased levels of anti-inflammatory cytokines (IL-10, IL-4) (Fig. 4i, Suppl. Fig. 21). Notably, the CSO_1_M_3_ membranes were proved to have the strongest immunomodulatory ability. IF staining results showed enhanced M2 polarization in the CSO_1_M_3_ group, which exhibited a markedly elevated M2/M1 ratio of ~14.5, significantly higher than other treatment groups (Fig. 4j and k). Meanwhile, the CSO_1_M_3_ membranes also markedly downregulated the expression of TNF-α in the tissues around the defective sites (Suppl. Fig. 22). These results were attributed to the controlled release of OTA and Mg²⁺ from the CSO_1_M_3_ membranes, which synergistically suppressed TNF-α-induced NF-κB activation by disrupting receptor engagement and downstream signaling pathways, thereby inhibiting the expression of pro-inflammatory cytokines, including IL-1β, TNF-α, and CCL5, while enhancing the expression of anti-inflammatory factors associated with M2 polarization, such as IL-10, CD206, and Arg-1 (Fig. 4l). Comparative analysis with the clinical CM products showed that the CSO_1_M_3_ unified bilayer membranes exhibited superior immunomodulatory effects, characterized by decreased pro-inflammatory factors expression and increased M2 polarization.

### Neurovascularization regulation of the unified bilayer membranes

Regulating immune microenvironment of the bone defects at early stages is critical for activating subsequent endogenous regeneration mechanisms^43–45^. As inflammation subsides, regeneration-related cells are recruited, and neurovascular networks are reconstructed in parallel (Stage III)^46,47^. Transwell migration results proved the CSO_1_M_3_ membranes significantly enhanced the recruitment of regeneration-related cells including *rat* schwann cells (RSCs), *human* umbilical vein endothelial cells (HUVECs), and *human* bone marrow stromal cells (hBMSCs) through the suppression of pro-inflammatory signaling and the induction of chemokine expression (Suppl. Figs. 23, 24). In addition, promoted cells attachment and spreading were observed on the CSO_1_M_3_ membranes compared with cells on the control group as well as on the CS and CSO_1_ membranes, as evidenced by significantly improved cytoskeletal structures of the RSCs, HUVECs, and hBMSCs (Suppl. Fig. 25). Similarly, CCK-8 assays and live/dead staining results proved qualitatively and quantitatively the best cells viability and proliferation of the RSCs, HUVECs, and hBMSCs co-cultured with the CSO_1_M_3_ membranes (Suppl. Fig. 26).

**Fig. 5:**
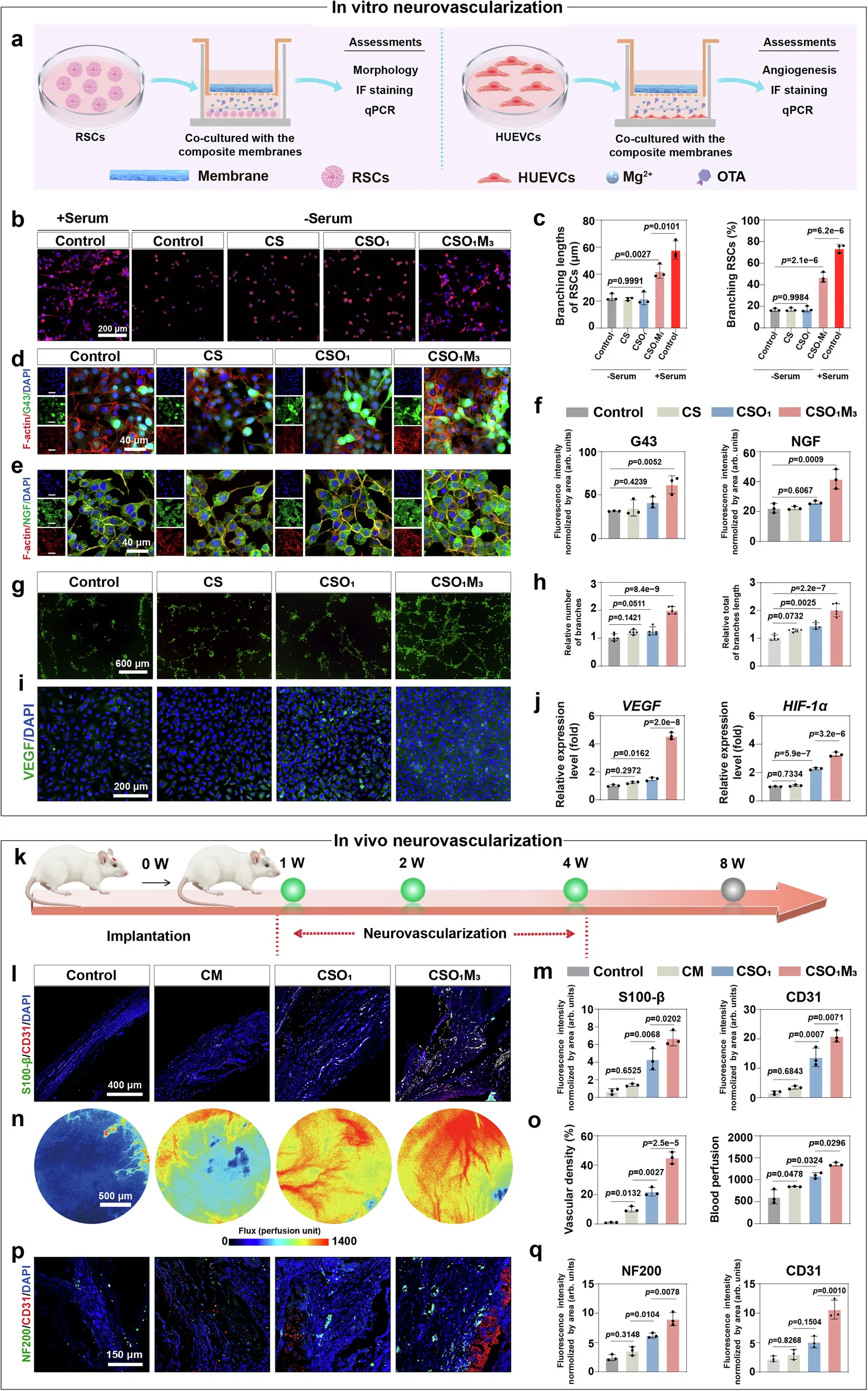
In vitro and in vivo neurovascularization regulation of the unified bilayer membranes. **a** Schematic of in vitro experimental design. **b**, **c** Representative images (**b**) and corresponding quantitative analysis (**c**) of the RSCs co-cultured in different groups after 24 h. **d**, **e** Representative IF staining of GAP-43 (**d**) and NGF (**e**) images of the RSCs co-cultured in different groups for 3 days. **f** Quantitative analysis of GAP-43 and NGF expressions of the RSCs based on the images in (**d**, **e**). **g**, **h** Representative images (**g**) and corresponding quantitative analysis (**h**) of the HUVECs tube formation in vitro. **i** Representative IF staining of VEGF images of the HUVECs co-cultured in different groups for 3 days. **j** qPCR analysis of *VEGF* and *HIF-1α* genes expressions of the HUVECs co-cultured in different groups for 3 days. **k** Schematic of in vivo experimental design using the *rat* cranial critical-sized defect model to continually evaluate neurovascularization at 1, 2, and 4 weeks post implantation. **l**, **m** Representative IF staining of S100-β (green) and CD31 (red) images of tissues from the defective areas (**l**) and corresponding quantitative analysis of the S100-β and CD31 expressions (**m**) at 1 week post implantation. **n**, **o** Laser speckle contrast imaging (LSCI) images (**n**) and corresponding quantitative analysis (**o**) of angiogenesis at the cranial defective sites at 2 weeks post implantation. **p**, **q** Representative IF staining of NF200 (green) and CD31 (red) images of tissues from the defective areas (**p**) and corresponding quantitative analysis of the NF200 and CD31 expressions (**q**) at 4 weeks post implantation. Values in (**c**), (**f**, **j**, **m**, **o**, **q**) represent the mean and standard deviation (*n*  =  3 independent samples per group). Values in (**h**) represent the mean and standard deviation (*n*  =  5 independent samples per group). Statistical analyses were performed using one-way ANOVA followed by Tukey’s post hoc test. *p* < 0.05 was considered statistically significant differences between the compared groups. Source data are provided as a Source Data file.

Based on these, we further investigated the regulation of the composite membranes on neurovascularization in vitro and in vivo. Morphological changes of the RSCs co-cultured with different membranes were evaluated qualitatively and quantitatively (Fig. 5a). After 24 h of co-culture under serum-free conditions, neither branching length nor the proportion of branched RSCs differed significantly among the control, CS, and CSO_1_ groups (Fig. 5b, c). Notably, the CSO_1_M_3_ group exhibited a ~ 1.8-fold increase in branching length and a ~ 2.8-fold increase in the proportion of branched RSCs compared to the control group. This suggested that the CSO_1_M_3_ membranes could enhance the conversion of RSCs into repair Schwann cells (repair SCs), thereby facilitating the formation of the Büngner bands that were crucial for guiding nerve repair^48^. The expressions of growth-associated protein 43 (GAP-43) and nerve growth factor (NGF) after 3-days co-culture with the membranes under standard serum conditions were investigated (Fig. 5d–f). IF staining images indicated that the CSO_1_M_3_ group showed higher expressions of the GAP-43 and NGF factors than the other groups. Quantitative analysis suggested a ~ 97% improvement of GAP-43 expression and ~88% improvement of NGF levels of the CSO_1_M_3_ group compared to the control group. Consistently, the CSO_1_M_3_ membranes were proved to significantly enhance the mRNA expression of *Ngf* and brain-derived neurotrophic factor (*Bdnf*) of the RSCs, which were beneficial for nerve regeneration (Suppl. Fig. 27). In addition, PC12 cells were co-cultured with different membranes to investigate the neurodifferentiation. Among all groups, the CSO_1_M_3_ membranes had the strongest ability to promote axonal elongation of the PC12 cells, which was critical for supporting neuroregeneration around the defective sites (Suppl. Fig. 28). Angiogenesis regulated by the membranes was subsequently evaluated. The scratch assay using the HUVECs suggested that the CSO_1_M_3_ group significantly enhanced cells migration after 16 h compared to the control, CS, and CSO_1_ groups (Suppl. Fig. 29). Tube formation assays confirmed that the CSO_1_M_3_ group had significantly more tube-like structures than those of other groups, with a ~ 2-fold increase in both branch number and total tube length relative to those of the control group (Fig. 5g, h). IF staining images showed that the CSO_1_M_3_ membranes significantly enhanced VEGF expression in the HUVECs (Fig. 5i, Suppl. Fig. 30). qPCR results further indicated the significant upregulation of *VEGF* and hypoxia-inducible factor 1-alpha (*HIF-1α*) expressions in the CSO_1_M_3_ group, proving the pro-angiogenic regulation of the CSO_1_M_3_ membranes in vitro (Fig. 5j).

To systematically investigate the dynamic neurovascular regeneration, the *rat* models for the immunomodulation experiments were continually used to conduct in vivo neurovascularization studies (Fig. 5k). After implanting the membranes for 1 week, the IF staining images showed the S100β (RSCs marker) and CD31 (HUVECs marker) expressions were significantly upregulated in the CSO_1_M_3_ group compared to the control, CM and CSO_1_ groups, which might be attributed to the sustained release of the bioactive Mg^2+^ (Fig. 5l, m). Similarly, the CSO_1_M_3_ membranes were also proved to enhance the co-localized expressions of NGF and VEGF (Suppl. Fig. 31). Therefore, the results indicated that the CSO_1_M_3_ membranes could regulate positively the behaviors and functions of the neural and vascular cells at the beginning of the neurovascular regeneration (1w) after constructing an antibacterial and anti-inflammatory microenvironment at the bone defective sites.

After implantation 2 weeks, the newly formed blood vessels and blood perfusion at the neoperiosteal region overlying the original cranial defect were investigated through the laser speckle contrast imaging (LSCI) method that could generate real-time maps of microvascular blood flow and surface perfusion. Compared to the control and the CM groups, the CSO_1_ and CSO_1_M_3_ groups had prominent new blood vessel formation (Fig. 5n and Suppl. Fig. 32). Moreover, the CSO_1_M_3_ group showed an ~2.1-fold increase of vascular density and a ~ 1.2-fold increase in blood perfusion at the cranial defective sites compared with the CSO_1_ group (Fig. 5o). The newly formed blood vessels could supply essential oxygen and nutrients, and support the recruitment and activity of osteogenic cells, thereby accelerating bone formation and defect healing^49,50^. After 4 weeks of implantation, IF staining of neurofilament 200 (NF200) and CD31 images showed that the CSO_1_M_3_ group exhibited significantly stronger fluorescence intensities compared to other groups, where the newly formed nerve fibers were closely aligned with newly formed blood vessels (Fig. 5p, q). Calcitonin gene-related peptide (CGRP) is a sensory neuropeptide released from dorsal root ganglia, which has been proved to promote osteogenic differentiation and serve as a key marker of neuronal differentiation in vivo^51^. H-type vessels, a specialized subtype of capillaries, are closely associated with the recruitment of osteoprogenitor cells and activate bone formation. These vessels are typically characterized by high expression of endomucin (Emcn) and CD31^52^. Therefore, we also investigated the expressions of the CGRP and Emcn/CD31 at the bone defective sites after implantation for 4 weeks (Suppl. Figs. 33, 34). As expected, the CSO_1_M_3_ membranes significantly improved the expression of the CGRP and the formation of Emcn^+^/CD31^+^ H-type vessels, confirming their potent regulation to promote neurovascular regeneration.

### Osteogenesis regulation of the unified bilayer membranes

**Fig. 6:**
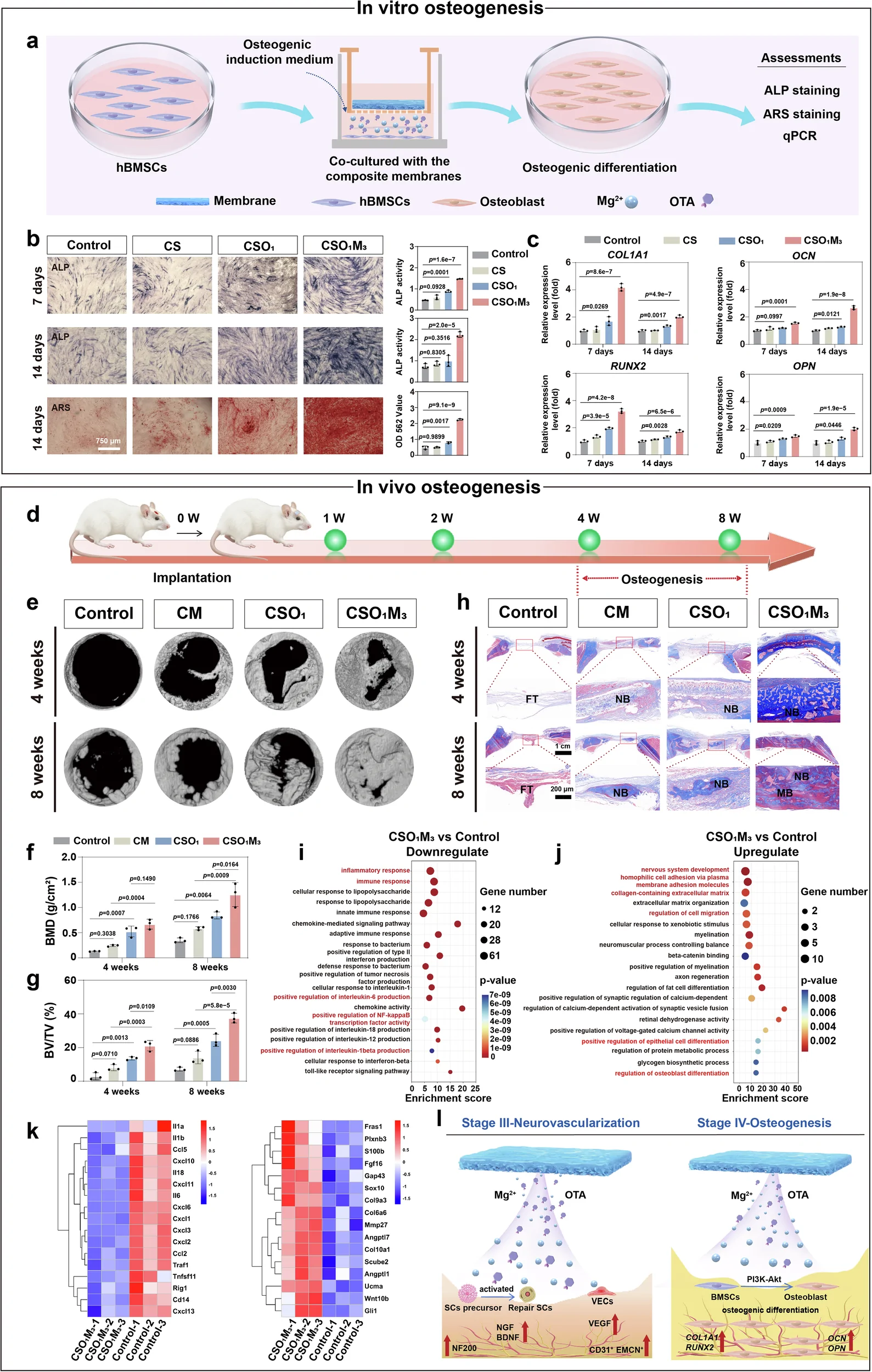
In vitro and in vivo osteogenesis regulation of the unified bilayer membranes. **a** Schematic of in vitro experimental design. **b** Qualitative and quantitative results of ALP staining of hBMSCs co-cultured with different membranes groups at 7 and 14 days, and ARS staining images of hBMSCs at 14 days. **c** qPCR analysis of osteogenic related genes (*COL1A1, OCN, RUNX2 and OPN*) expressions of hBMSCs co-cultured with different membranes groups at 7 and 14 days. **d** Schematic of in vivo experimental design using the *rat* cranial critical-sized defect model to evaluate continuously osteogenesis after implantation for 4 and 8 weeks. **e** Micro-CT 3D reconstruction images of the cranial defective sites after various membranes implantation for 4 and 8 weeks. **f**, **g** Quantitative analysis of BV/TV (**f**) and BMD (**g**) of different groups at 4 and 8 weeks. **h** Histological evaluation of tissues around bone defective sites through Masson’s trichrome staining (**h**). FT: fibrous tissue. NB: newly formed bone. MB: Mature bone. **i**, **j** Transcriptomic comparison between the CSO_1_M_3_ and control groups at 4 weeks post-implantation. GO enrichment analysis of downregulated (**i**) and upregulated (**j**) genes related to various biological responses and functions. Statistical analysis was performed using a one-sided hypergeometric test without adjustment for multiple comparisons. *p* < 0.05 was considered statistically significant differences between the compared groups. **k** Heatmap of differentially expressed genes. Red: upregulated genes. Blue: downregulated genes. **l** Schematic of the proposed mechanisms by which the CSO_1_M_3_ unified bilayer membranes regulated neurovascularization and osteogenesis. Values in (**b**, **c**, **f**, **g**) represent the mean and standard deviation (*n*  =  3 indepe*n*dent samples per group). Statistical analyses were performed using one-way ANOVA followed by Tukey’s post hoc test. *p* < 0.05 was considered statistically significant differences between the compared groups. Source data are provided as a Source Data file.

With robust neurovascular remodeling and microenvironmental normalization established, we sequentially evaluated the osteogenesis regulation (Stage IV) of the membranes. To investigate the osteogenic differentiation of hBMSCs enhanced by the membranes, hBMSCs were co-cultured with CS, CSO_1_, and CSO_1_M_3_ membranes in transwell respectively (Fig. 6a). The alkaline phosphatase (ALP) activity of hBMSCs was measured with ALP staining after 7 days co-culture, and the calcium nodules were measured with Alizarin Red S (ARS) staining after 14 days. The images showed that the purple ALP-stained colonies and red mineralized nodules in the CSO_1_M_3_ group were more abundant and darker than those in the other groups at each time point. Quantitative analysis confirmed that the CSO_1_M_3_ membranes significantly enhanced ALP activity and mineralization of the hBMSCs (Fig. 6b). Furthermore, qPCR results indicated that the CSO_1_M_3_ membranes significantly upregulated osteogenic related genes expressions of the hBMSCs including collagen type I alpha 1 chain (*COL1A1*), osteocalcin (*OCN*), runt-related transcription factor 2 (*RUNX2*), and osteopontin (*OPN*) compared to the control groups at all time points (Fig. 6c). IF staining of Runx2 and OCN images presented significantly enhanced fluorescence intensities in the CSO_1_M_3_ group compared to other groups, confirming the upregulated expression of these osteogenic proteins (Suppl. Fig. 35). The pro-osteogenic effects of the composite membranes on *human* periosteum-derived stem cells (hPDSCs) were also demonstrated by CCK-8 assay, live/dead staining, as well as ALP and ARS staining methods (Suppl. Figs. 36, 37).

The critical-sized cranial defect models were sequentially observed and analyzed (Fig. 6d). Tissue samples from the defective sites were harvested after implanting for 4 and 8 weeks. Micro-computed tomography (micro-CT) reconstruction images showed obviously more new bone in the CSO_1_M_3_ group compared with the control, CM and CSO_1_ groups (Fig. 6e). Particularly, the defective sites were nearly filled with newly formed bones in the CSO_1_M_3_ group at 8 weeks. Quantitative analysis of the percentages of the bone volume to tissue volume (BV/TV) and the local volumetric bone mineral density (BMD) confirmed the significantly enhanced osteogenic regulation of the CSO_1_M_3_ membranes (Fig. 6f, g). At 8 weeks, the BMD and BV/TV in the CSO_1_M_3_ group reached ~1.26 g cm^−^^3^ and ~37.6%, while those in the CM group were only ~0.59 g cm^−3^ and ~14.1%, respectively. H&E and Masson’s trichrome staining results demonstrated reduced inflammation and enhanced new bone formation in the CSO_1_M_3_ group compared to those of the other groups after 4 and 8 weeks of implantation (Fig. 6h and Suppl. Fig. 38). IF staining images showed higher OCN expression in the CSO_1_M_3_ group at both 4 and 8 weeks compared with the control, CM, and CSO_1_ groups (Suppl. Fig. 39). Furthermore, the histological examinations on major organs (heart, kidney, liver, lung, and spleen), indicating good biocompatibility and biosafety of the unified bilayer membranes (Suppl. Fig. 40).

To further investigate the mechanisms of in vivo osteogenesis regulation of the CSO_1_M_3_ membranes, transcriptomic sequencing was conducted using tissues at defective sites collected from the control and CSO_1_M_3_ groups after implantation for 4 weeks. The volcano plot identified 558 upregulated and 156 downregulated genes in the CSO_1_M_3_ group (Suppl. Fig. 41). GO enrichment analysis revealed significant downregulation of the genes sets related to inflammatory response, immune response, and bacterial defense in the CSO_1_M_3_ group at 4 weeks post implantation (Fig. 6i). These results confirmed the sustainable antibacterial and anti-inflammatory effects of the CSO_1_M_3_ membranes. In addition, upregulated GO terms were enriched associated with bone regeneration, including nervous system development, cell migration, epithelial differentiation, and osteoblast differentiation (Fig. 6j). A heatmap of differentially expressed genes showed downregulation of inflammatory markers including *Tnf*, *Il-1α*, *Il-1β* and *Ccl5*, and upregulation of pro-regenerative genes including *Gap43*, *Fgf13*, *Col9a3* and *Mmp27* (Fig. 6k). KEGG pathway enrichment analysis of differentially expressed genes revealed the downregulated pathways in the CSO_1_M_3_ group including cytokine-cytokine receptor interaction, TNF signaling pathway, and NF-κB signaling pathway, consistent with the in vitro immunomodulation experiments (Suppl. Fig. 42a). Additionally, the phosphoinositide 3-kinase (PI3K)-Akt (PI3K-Akt) signaling pathway was highlighted in the KEGG analysis of upregulated pathways (Suppl. Fig. 42b). Previous studies have demonstrated that the activation of the PI3K-Akt signaling pathway could promote the proliferation, migration and differentiation of osteoblasts^53,54^. Thus, the phosphorylated PI3K (p-PI3K) and phosphorylated Akt (p-Akt) as essential activation nodes within this pathway were further evaluated^55,56^. The results showed p-PI3K expression was upregulated in the CSO_1_M_3_ group at 4 weeks post implantation (increased by ~7.6-fold and ~4.0-fold compared with the control and CM groups, respectively) (Suppl. Fig. 43). In addition, the expression of p-Akt/Runx2 was also significantly increased in the CSO_1_M_3_ group (Suppl. Fig. 44). Based on the experimental results, Fig. 6l summarized the mechanisms of neurovascularization and osteogenesis through regulation of the CSO_1_M_3_ membranes. Specifically, the CSO_1_M_3_ membranes facilitated the coordinated regeneration of nerves and blood vessels by upregulating the growth factors and markers including NGF, BDNF, CD31, and VEGF for reconstructing neurovascular networks. After that, the Mg²⁺ released from the CSO_1_M_3_ membranes further activated the PI3K-Akt signaling pathway to upregulate osteogenic differentiation-related genes and proteins including *COL1A1, OCN, RUNX2, and OPN*, therefore promoting proliferation, differentiation, and mineralization of osteoblasts. Meanwhile, the CSO_1_M_3_ membranes could provide a sustainable antibacterial and anti-inflammatory microenvironment through releasing Mg^2+^ and OTA during the regeneration process, which ensured the enhancements of the CSO_1_M_3_ unified bilayer membranes on regulating ultimate bone regeneration.

## Discussion

The clinical utility of degradable bilayer bioactive membranes in bone repair is constrained by poor hydrated-state mechanical performance and bioactivity that does not adapt to the prolonged course of healing. Firstly, degradation-induced discontinuities at the bilayer interface undermine interlaminar cohesion, leading to delamination, shape instability, and early disintegration under fluid ingress and mechanical loading; as a result, space maintenance and barrier function against fibroblast ingrowth are compromised, yielding poor bone regeneration^57^. Secondly, antibacterial and bioactive regulation is often temporally mismatched and non-adaptive, failing to deliver tailored effects across the healing cascade stages including antibacteria (Stage I), immunomodulation (Stage II), neurovascularization (Stage III), and osteogenesis (Stage IV).

In this study, the CSO_1_M_3_ membranes were fabricated through a homogeneous isomeric in-situ forming strategy to construct a unified bilayer structure. This strategy can also be extended to other material systems. Specifically, natural polysaccharides (e.g., chitosan, sodium alginate, hyaluronic acid, and gelatin) are the primary choice for the precursor solution due to their good biocompatibility, tunable solubility, and ability to undergo in-situ crosslinking/phase transformation. Additionally, polyphenol derivatives (e.g., OTA and oxidized epigallocatechin gallate) act as dual-functional crosslinkers that strengthen the bilayer interface through covalent and non-covalent interactions. Besides, bioactive inorganic/organic nanoparticles (e.g., MgO, CaO, and hydroxyapatite) can be incorporated as reinforcing fillers to enhance mechanical performance and enable sustained release of bioactive ions (e.g., Mg^2+^, Ca^2+^). By adjusting these material combinations, the strategy could also be used to fabricate unified bilayer membranes for repairing damaged skin, cartilage and other tissues.

Due to the Schiff-base linkages and phenolic coordination systems in the molecular network, the barrier layer and porous layer of the membranes were unified with interfacial continuities for ensuring superior mechanical properties. The CSO_1_M_3_ membranes exhibited a tensile strength of ~156 MPa and a toughness of ~72 MJ m^−3^ under dry conditions, and a tensile strength of ~8.6 MPa and a toughness of ~5.4 MJ m^−3^ under hydrated conditions, which provided satisfactory protection and conformability to the irregular bone defective sites in a long period. In addition, the bioactive components OTA and Mg^2+^ could be sequentially released for ≥ 56 days to regulate various stages during bone healing process. Due to triple antibacterial mechanisms (releasing killing, contact killing, and barrier effects) at Stage I, the CSO_1_M_3_ membranes contributed to respective death rates of ~90% for *S. aureus* and ~93% for *E. coli*, as well as ~100% antibacterial exclusion for both *S. aureus* and *E. coli*. The unified bilayer membranes could also effectively prevent the ingrowth of the fibroblasts to the bone defective sites. At Stage II, the CSO_1_M_3_ membranes regulated immune responses by inhibiting the TNF-α/NF-κB signaling pathway to promote M2 macrophages polarization. Accordingly, endogenous regeneration-related cells e.g., RSCs, HUVECs, and BMSCs were recruited collectively to accelerate inflammation diminution and initiate tissue regeneration. More importantly, the sequential release of bioactive Mg^2+^ from the CSO_1_M_3_ membranes improved neurovascular ingrowth by upregulating the growth factors and markers including NGF, BDNF and VEGF, CD31 at Stage III. In vivo studies showed the CSO_1_M_3_ membranes achieved an ~4.5-fold increase of vascular density and a ~ 1.6-fold increase in blood perfusion at the cranial defective sites compared to the commercial products. In addition, a triple-color immunofluorescence staining results suggested the TUBB3^+^ nerves and CD31^+^ blood vessels were not randomly distributed, but closely surrounded Osterix^+^ newly formed bone tissues in all groups at 4 weeks post-implantation (Suppl. Fig. 45). This specific spatial arrangement indicated these three systems might have a mutually reinforcing relationship. In particular, the CSO_1_M_3_ membrane induced significantly improved new-tissue formation and coupling intensity in the surrounding region. At subsequent Stage IV, the Mg^2+^ released from the CSO_1_M_3_ membranes persistently regulated osteogenesis through activating the PI3K-Akt pathway to enhance osteoblasts’ proliferation, differentiation, and mineralization. After being implanted in vivo for 8 weeks, the BMD and BV/TV in the CSO_1_M_3_ group were ~1.26 g cm^−^^3^ and ~37.6%, while those in the CM group were only ~0.59 g cm^−3^ and ~14.1%, respectively.

Generally, bones were covered by neural and vascular networks essential for the development, remodeling, and repair^58^. Specifically, nerve regeneration regulated osteogenic differentiation and bone metabolism via neuropeptides and cytokines (e.g., CGRP, NGF). The neural pathways could also guide the spatial alignment of newly formed bone tissues. Angiogenesis established a nutrient supply network to supply the basic necessities (e.g., oxygen, nutrients, growth factors) and remove the waste products for the defect region. Blood vessels also played a key role in the recruitment of osteoprogenitor cells. Additionally, nerves and blood vessels interacted synergistically, which nerves promoted angiogenesis via neurotransmitter secretion, and blood vessels in turn supported nerve fiber growth by supplying oxygen and nutrients. Upregulated GO terms including nervous system development, epithelial differentiation, and osteoblast differentiation in Fig. 6j and Suppl. Fig. 46 also confirmed that the CSO_1_M_3_ group significantly enhanced the synergistic interaction among these new tissues compared to the control group. The CSO_1_M_3_ membrane was proved to establish a well-integrated spatial structure, highlighting its superior bioactivity for regulating stage-adaptive bone regeneration.

**Fig. 7:**
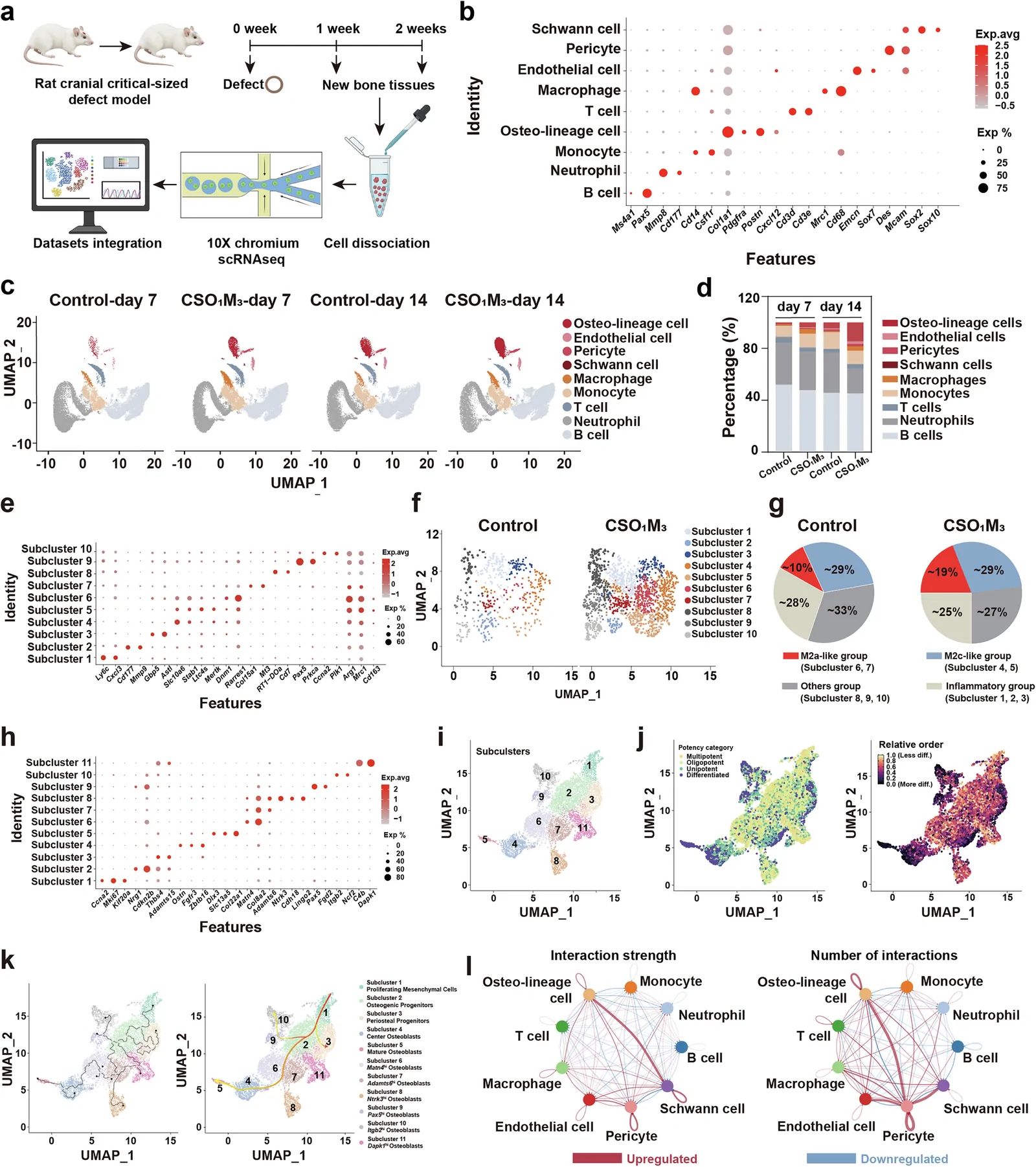
scRNA-seq of the regenerative bone tissues from the control and CSO_1_M_3_ groups. **a** Flowchart of the single-cell transcriptome study on a rat cranial critical-sized defect model at different time points. Created in BioRender. Gao, C. (2026) https://BioRender.com/9uclu7y. **b** Dot plots of marker genes expression to define 9 distinct cell clusters, including B cell, Neutrophil, T cell, Monocyte, Macrophage, Osteo-lineage cell, Endothelial cell, Pericyte, and Schwann cell. Dot size represented the proportion of cells expressing a given gene in the cluster, and the color bar represented the gene expression levels. **c** UMAP visualization of the 9 cell clusters in the control and CSO_1_M_3_ groups after 1 and 2 weeks post-surgery, respectively. **d** Relative proportion of cell populations among the above four groups. Values represent the mean (*n*  =  3 independent samples per group). **e** Dot plots of marker genes expression to define 10 cell subclusters of macrophages in the control and CSO_1_M_3_ groups after 1 week post-surgery. Dot size represented the proportion of cells expressing a given gene in the cluster, and the color bar represented the gene expression levels. **f** UMAP visualization of the 10 macrophage subclusters. **g** Quantitative analysis of functional groups of the macrophage subpopulations in the control and CSO_1_M_3_ groups. Values represent the mean (*n*  =  3 independent samples per group). **h** Dot plots displaying the expression of representative marker genes for 11 cell subclusters of the OLCs in the control and CSO_1_M_3_ groups after 1 and 2 weeks post-surgery. **i** UMAP visualization of the 11 OLC subclusters. **j** CytoTRACE analysis illustrating the OLCs differentiation potential. Left: Cellular potency. Right: Relative differentiation order. **k** Developmental trajectory of the OLCs inferred by Monocle 3. Left: Pseudotime trajectory colored by subclusters. Right: Schematic of the predictable trajectory. **l** Cell-cell communication analysis between the control and CSO_1_M_3_ group at 1 week post implantation. Red lines: upregulated interactions. Blue lines: downregulated interactions. Cell clusters: B cell, Neutrophil, T cell, Monocyte, Macrophage, Osteo-lineage cell, Endothelial cell, Pericyte, and Schwann cell. Source data are provided as a Source Data file.

To further explore the underlying mechanism of bone repair, the single-cell RNA sequencing (scRNA-seq) was performed on harvested newly formed tissues from the control and CSO_1_M_3_ groups (Fig. 7a). Dot plots and Uniform Manifold Approximation and Projection (UMAP) were calculated to visualize cell heterogeneity (Fig. 7b and Suppl. Fig. 47). 9 distinct cell clusters were identified based on marker gene expression, including B cell (*Pax5, Ms4a1*), Neutrophil (*Mmp8, Cd177*), T cell (*Cd3d, Cd3e*), Monocyte (*Csf1r, Cd14*), Macrophage (*Cd68, Mrc1*), Osteo-lineage cell (*Col1a1, Postn*), Endothelial cell (*Emcn, Sox7*), Pericyte (*Des, Mcam*), and Schwann cell (*Sox2, Sox10*)^59,60^. These results provided a single-cell atlas of the regenerative bone tissues to reveal the cellular heterogeneity during repair. Notably, the CSO_1_M_3_ group exhibited a higher proportion of osteo-lineage cells than the control group during the first two weeks, with a sequential increase (Fig. 7d).

To dissect cellular heterogeneity and classify the macrophage subsets, we merged macrophages obtained from the control group and CSO_1_M_3_ group at 1 week post-implantation for subclustering. Based on marker gene expression, 10 macrophage subclusters were identified, including Subcluster 1 (*Cxcl3, Ly6c*), Subcluster 2 *(Mmp9, Cd177)*, Subcluster 3 *(Gbp5, Astl)*, Subcluster 4 *(Slc10a6, Stab1)*, Subcluster 5 *(Mertk, Ltc4s)*, Subcluster 6 *(Dnm1, Rarres1)*, Subcluster 7 *(Col5a1, Mt3)*, Subcluster 8 *(RT1-Doa, Cd7)*, Subcluster 9 *(Pax5, Prkca)*, and Subcluster 10 *(Ccna2, Plk1)* (Fig. 7e, f, Suppl. Fig. 48). According to published reports, these subclusters could be categorized into four groups, including the Inflammatory macrophage group (Subcluster 1-3), M2c-like macrophage group (Subcluster 4-5), M2a-like macrophage group (Subcluster 6-7), and Others macrophage group (Subcluster 8-10). In particular, the M2c-like group was associated with clearing cellular debris and suppressing inflammation, and the M2a-like group contributed to tissue repair and remodeling through secreting regenerative factors^61,62^. In addition, qualitative and quantitative analysis revealed that the CSO_1_M_3_ membranes markedly increase the population of the M2a-like group during the early stage of bone repair (Fig. 7g).

The osteo-lineage cells (OLCs) in all the groups at 1 and 2 weeks post-implantation were also merged to analyze the cell heterogeneity. We identified and visualized the OLCs subpopulations through dot plots and UMAP analysis based on the clustering-specific markers (Fig. 7h and Suppl. Fig. 49). As a result, the OLC subpopulations were annotated as follows: Subcluster 1 represented the Proliferating Mesenchymal Cells (expressing *Ccna2, Kif2c*); Subcluster 2 represented the Osteogenic Progenitors (expressing *Cdkn2b, Nrg1*); Subcluster 3 represented the Periosteal Progenitors (expressing *Thbs4, Adamts15*); Subcluster 4 represented Center Osteoblasts (expressing *Ostn, Fgfr3*); Subcluster 5 represented Mature Osteoblasts (expressing *Slc13a5, Dlx3*); Subcluster 6 represented *Matn4*^hi^ Osteoblasts (expressing *Matn4, Ucma*); Subcluster 7 represented *Adamts6*^hi^ Osteoblasts (expressing *Adamts6, Col8a2*); Subcluster 8 represented *Ntrk3*^hi^ Osteoblasts (expressing *Ntrk3, Lingo2*); Subcluster 9 represented *Pax5*^hi^ Osteoblasts (expressing *Pax5, Fgd2*); Subcluster 10 represented *Itgb2*^hi^ Osteoblasts (expressing *Itgb2, Ncf2*); and Subcluster 11 represented *Dapk1*^hi^ Osteoblasts (expressing *Dapk1, C4b*) (Fig. 7i)^63^. Furthermore, we performed CytoTRACE analysis to evaluate the cell differentiation potential, which identified the Proliferating Mesenchymal Cells (Subcluster 1) as the population with the highest stemness (Fig. 7j). Consequently, we defined Subcluster 1 as the root for the subsequent pseudotime analysis. Afterwards, Monocle 3 was employed to construct the single-cell trajectories, forming a clear, continuous developmental progression (Fig. 7k). The trajectory originated from the high-stemness proliferating pool (Subcluster 1) and first toward the functional state of the Osteogenic Progenitors (Subcluster 2). Then, trajectory bifurcated successively to the Periosteal Progenitors (Subcluster 3) and the osteoblasts (Subcluster 9-10). Notably, a major lineage commitment was identified moving through the Osteogenic Progenitors (Subcluster 2) and ultimately advancing toward osteoblasts (Subcluster 7-4-5). This trajectory illustrated the transition of the OLCs from an undifferentiated and proliferative state to a functionally specialized mineralizing state during the bone repair process.

Comprehensive CellChat analysis was also performed to evaluate cellular crosstalk among various cell clusters (Fig. 7l). The strength and number of interactions were indicated by the degree of line thickness. Comparison between the control and CSO_1_M_3_ groups at 1 week post-implantation revealed a pronounced remodeling of the cellular communication network. Specifically, the CSO_1_M_3_ group exhibited enhanced interaction strengths and numbers among key regenerative cell clusters (such as the Schwann cells, endothelial cells, and OLCs) relative to the control group. Within this optimized regenerative niche, Schwann cells and endothelial cells exhibited a robust, synchronized crosstalk with the OLCs. These results collectively demonstrated that the CSO_1_M_3_ membrane could promote a tightly integrated neurovascular‑osteogenic communication network, thereby effectively improving bone regeneration.

Consequently, these results indicated the CSO_1_M_3_ bilayer membranes integrated reinforced mechanical stability with stage-adaptive regulation through the unified bioactive architecture, which concurrently addressed above two key challenges during long-term healing process. In addition, there are also some limitations. For example, in vivo studies in large animal models have not been performed in this work. The membranes were fabricated from naturally derived biomaterials via a simple and low-cost process, offering strong scalability and clinical translation potential. Therefore, we will further investigate longer-term efficacy and safety of the unified bilayer membranes in large animal models to delineate their practical utilization for bone repair.

## Methods

### Materials

CS (viscosity > 400 mPa·s), OTA, and NaOH (analytical grade) were purchased from Aladdin (Shanghai, China). MgO nanoparticles were obtained from Nanjing XFNANO Materials Tech Co., Ltd. (Nanjing, China). Lysozyme and LPS (from *E. coli* O111:B4) were purchased from Sigma-Aldrich (Shanghai, China). CM were purchased from Geistlich Pharma AG (Switzerland), marketed under the trade name Geistlich Bio-Gide®. DMEM, α-MEM, FBS, and P/S were obtained from Gibco (Thermo Fisher, USA). Live/Dead kit, DAPI, Alexa Fluor 555-phalloidin, Fluoromount-G, and secondary antibodies were purchased from Thermo Fisher (USA). hBMSCs was obtained from Cyagen (Suzhou, China). RAW264.7, PC12, L929, and HUVECs were purchased from ATCC (Shanghai, China). RSCs and hPDSCs were obtained from iCell (Shanghai, China). qPCR primers and reagents were from Takara (Japan). Antibodies for IF was sourced from Abcam (Shanghai, China), Biorbyt (Wuhan, China), Proteintech (Wuhan, China), Bioss (Beijing, China), Beyotime (Shanghai, China), Sigma-Aldrich (Shanghai, China), Invitrogen (Shanghai, China), and Thermo Fisher (Shanghai, China), as listed in Suppl. Table 1.

### Preparation of the unified bilayer membranes

CS powders were dissolved in 1 v v^−1^% acetic acid solution to prepare final 2 w v^−1^% CS solution. Subsequently, different mass fractions of OTA and MgO aqueous solution were mixed into the CS solution. The pH value of the mixture was adjusted to 6.0 using 3 M NaOH solution with vigorous stirring. Then, the solution was cast into Petri dishes (Corning, Guangzhou, China) followed by being dried at 40 °C to form the barrier layer of the membranes. After that, the solution was spray-coated onto the dried membranes followed by immediately flash-freezing the whole membranes using liquid nitrogen. The frozen membranes were immersed in precooled (−20 °C) 3 M NaOH solution to neutralize residual acetic acid. After being triple rinsed with deionized water, the samples were lyophilized to yield the final unified bilayer membranes. According to the usage of the CS, OTA, and MgO nanoparticles, the membranes were prepared as CS membranes (pure CS), CSOx membranes (CS with x wt% OTA), and CSOxMy membranes (CS with x wt% OTA and y wt% MgO nanoparticles).

### Characterization of unified bilayer membranes

FTIR spectra was used to analyze the variations of chemical bonds in the membranes by an FTIR spectrometer (Thermo Fisher, Nicolet 6700, USA). XPS was conducted to confirm the formation of Schiff-base linkages and incorporation of MgO nanoparticles by an XPS spectrometer (Thermo Fisher Scientific, USA). Raman spectra of the membranes were recorded using a LabRAM ARAMIS system (Horiba-Jobin Yvon, France). For microstructural analysis of cross-sections, membranes were cryofractured in liquid nitrogen. The morphological characterization of the surfaces and cross-sections of the membranes was conducted by the SEM (Sigma 300, Carl Zeiss, Germany) at 5 kV acceleration voltage. Elemental composition in the membranes was analyzed using an EDS spectrometer (XFlash 6130, Bruker, Germany). Flexibility assessment: composite membranes were manually curled, folded, and bent at room temperature followed by being recorded using a smartphone. Surface roughness of the barrier and porous layers of the membranes was quantified via 3D optical profilometry (Bruker, ContourGT-K, Germany). Wettability of the sample was measured using a static contact angle meter (Dataphysics, OCA, 15EC, Germany). The composite membranes were sutured using standard surgical sutures, and the suture hole was observed by the SEM (Sigma 300, Carl Zeiss, Germany) at 5 kV acceleration voltage. To evaluate the swelling behaviors, the membranes were first cut into a size of 1 cm × 1 cm and the initial dry mass of the square sample was recorded. Each sample was immersed in 10 mL of PBS (pH = 7.4) for various periods (2, 4, 8, 12, and 24 h). At each time point, membranes were removed, gently blotted to remove surface moisture on the membranes was removed followed by being weighed. The swelling ratio was calculated using the Eq. (1):

1 $${{\rm{Swelling\; ratio}}}=\frac{{M}_{1}-{M}_{0}}{{M}_{0}}\times 100\%$$

Where *M*_*0*_ (g) is the weight of the original sample and *M*_*1*_ (g) is the weight of the swollen sample at 2, 4, 8, 12, and 24 h, respectively. Mechanical properties of the membranes before and after degradation for 56 days were investigated using an electronic universal testing machine (TSE503C, WANCE, China). Membranes were cut to a size of ~3 mm (width) × ~50 mm (length) and the testing rate was 1 mm min^−1^ at room temperature. Enzymatic degradation of the membranes was conducted using the square sample with a size of 1 cm × 1 cm. Each sample was immersed in 10 mL of PBS (pH = 7.4) containing 1.5 µg mL^−1^ lysozyme (from chicken egg white, Sigma-Aldrich) at 37 °C in a shaking water bath at 60 rpm. To maintain enzymatic activity, fresh lysozyme was supplemented into the incubation medium every two days. At predetermined time points (3, 7, 14, 28, and 56 days), the samples were rinsed thoroughly with deionized water and then vacuum-dried using a vacuum oven (DZF-6050, Shanghai Yiheng Scientific Instrument Co., China). The dried samples were then weighed to obtain the residual weight (W_t_). The residual ratio was calculated using the Eq. (2):

2 $${{\rm{Weight}}}\; {{\rm{residual}}}\; {{\rm{ratio}}}=\frac{{W}_{t}}{{W}_{0}}\times 100\%$$

Where *W*_*0*_ (g) is the weight of the original sample and *W*_*t*_ (g) is the weight of the sample after degradation for 3, 7, 14, 28, and 56 days, respectively. The pH value of the degradation medium at each time point was measured directly using a calibrated pH meter (S220 SevenCompact™, Mettler Toledo, Switzerland). The cumulative release of OTA was quantified by ultraviolet-visible (UV-Vis) spectroscopy using a UV-2600 spectrophotometer (Shimadzu, Japan). The release of Mg^2+^ ions was determined using inductively coupled plasma optical emission spectroscopy (ICP-OES, 700 Series, Agilent Technologies, USA) with an iCAP 7000 Series instrument (Thermo Fisher Scientific, USA).

### In vitro barrier and antibacterial effects

In vitro antibacterial performance of the composite membranes was evaluated through a series of experiments. The membranes were placed in a 96-well plate and incubated at 37 °C in a constant-temperature incubator (HF90, Heal Force, China) with a 10^5^ colony-forming units (CFUs) mL^−1^ suspension of *S. aureus* (ATCC 25923) or *E. coli* (ATCC 25922). Bacterial growth was measured at 2, 4, 8, 12, and 24 h using an automatic microplate reader (Epoch 2, BioTek, USA) to determine the absorbance at 600 nm. After being co-cultured with different composite membranes for 72 h, bacterial viability was assessed using the LIVE/DEAD^TM^ BacLight^TM^ bacterial viability detection kit (Thermo Fisher, USA). The staining samples were observed using a confocal laser scanning microscope (LSM 880, Zeiss, Germany). Simultaneously, the co-culture medium was collected to serially dilute followed by spreading onto LB agar plates (Rare Biotechnology Co., Ltd., China). After 24 h of incubation at 37 °C, CFUs were counted based on the recorded photographs to evaluate the antibacterial efficacy of the membranes. To investigate anti-adhesive properties and the barrier functions of the membranes against bacterial penetration, membranes were fixed to the bottom of the 24-well plates. A 300 µL bacterial suspension (10^8^ CFU mL^−1^) was applied on the surface of the barrier layer of the membranes and then incubated at 37 °C for 7 days, with the culture medium refreshed every 2–3 days. After 7 days, the membranes were rinsed with PBS and then fixed with 4% paraformaldehyde (Solarbio, Beijing, China). After that, the samples were dehydrated through a graded ethanol series (30–100%). The morphologies on the barrier layers and the porous layers of the membranes were observed by the SEM (Sigma 300, Carl Zeiss, Germany) at 5 kV acceleration voltage. Cytocompatibility of the membranes with L929 cells were quantified through the CCK-8 assay (CK04-500, Dojindo, Kumamoto, Japan).

### In vitro immunomodulation

RAW264.7 murine macrophages were expanded in Dulbecco’s modified Eagle medium (DMEM, high glucose, Gibco, USA) supplemented with 10 v v^−^^1^% fetal bovine serum (FBS, Gibco, USA) and 1% penicillin-streptomycin (Gibco, USA) at 37 °C in 5% CO_2_ atmosphere. For proliferation investigation, cells were seeded with a density of 5 × 10^3^ cells per well (Corning, USA) in 96-well flat-bottom plates that had been pre-covered with sterilized composite membranes. OD Values at 1, 3, and 5 days were quantified using the CCK-8 (CK04-500, Dojindo, Kumamoto, Japan) assay. To evaluate the behaviors of activated macrophages, RAW264.7 cells were pre-stimulated with 100 ng mL^−^^1^ LPS (L2630, Sigma-Aldrich, USA) for 4 h. After that, the cells were washed twice with sterile PBS solution followed by being reseeded with a density of 2 × 10^5^ cells per well onto the 24-well plates that had been pre-covered with sterilized composite membranes. After 48 hours of co-culture, cells were fixed in 4 w v^−^^1^% paraformaldehyde (Electron Microscopy Sciences, USA) for 30 min. After being rinsed 3 times with PBS solution, the samples were permeabilized with 0.1 v v^−^^1^% Triton X-100 (T8787, Sigma-Aldrich, USA) for 10 minutes at room temperature. The actin cytoskeletons were labelled with Alexa Fluor 555-phalloidin (CA1610, Solarbio) and nuclei were counter-stained with 4’,6-diamidino-2-phenylindole (DAPI, C1006, Beyotime). After being washed twice by the PBS solution, the samples were mounted in Fluoromount-G (Invitrogen, USA) and observed by a confocal microscope (LSM 900, Zeiss, Germany). RAW264.7 macrophages were seeded in glass-bottom confocal dishes with a density of 2 × 10^5^ cells per well and then co-cultured with composite membranes in the presence of LPS (100 ng mL^−1^, Sigma-Aldrich, USA) for 48 h. After being fixed, sealed, and lysed, the cells were incubated with anti-CD163 (Biorbyt, orb1333718) and anti-CD86 (Biorbyt, Orb500820-cf594) overnight at 4 °C. After being counter-stained by secondary antibodies and DAPI (C1006, Beyotime), the samples were observed by a laser-scanning confocal microscope (LSM 900, Zeiss, Germany). Total RNA was extracted using the TRIzol reagent kit (CW0581M, CWBIO, China) following the manufacturer’s instructions. RNA concentrations and purities were determined using a NanoDrop 2000 spectrophotometer (Thermo Fisher Scientific, USA). RNA integrity was assessed with an Agilent 2100 Bioanalyzer (Agilent Technologies, USA). Complementary DNA (cDNA) was synthesized using the PrimeScript^TM^ RT reagent kit (Takara, Japan). qPCR experiments were performed on a QuantStudio 6 Real-Time PCR System (Thermo Fisher Scientific, USA) using the TB Green® Premix Ex Taq^TM^ II kit (Takara, Japan). Glyceraldehyde-3-Phosphate Dehydrogenase (GAPDH) was used as the internal reference, and relative gene expression was calculated using the 2^−△△Ct^ method. The target genes included *Il6*, *Ccl2*, *Tnf*, *Arg1*, *Mrc1*, *Il10*. The used primer sequences (Sangon Biotech Co., Ltd., Shanghai, China) were listed in Suppl. Table 2. mRNA libraries were prepared using the TruSeq Stranded mRNA LT Sample Prep Kit (Illumina, USA) and sequenced on the HiSeq X Ten platform (Illumina, USA) with 150 bp paired-end reads (≥ 10 Gb per sample) by OE Biotech Co. (Shanghai, China). Raw reads were subjected to quality control with FastQC. Low-quality reads and adapter sequences were trimmed using Trimmomatic. Clean reads were aligned to the *mouse* reference genome (GRCm39) using HISAT2. Transcript abundance was quantified as FPKM values using StringTie. Differentially expressed genes were identified using DESeq2 (|log₂Fold Change | ≥ 1, false discovery rate ≤ 0.05). GO and KEGG enrichment analyses were conducted using the clusterProfiler package (*P* ≤ 0.05). After 48 h of co-culture, culture media were harvested from each well and cleared by centrifugating with a speed of 1000 g for 10 min at 4 °C. The concentrations of IL-6 and TNF-ɑ were quantified with DuoSet ELISA kits (R&D Systems, USA) in accordance with the manufacturer’s instructions.

### In vitro cells recruitment, proliferation, and adhesion

RSCs, HUVECs, and hBMSCs were cultured at 37 °C in a humidified 5% CO_2_ incubator. RSCs and HUVECs were cultured using high-glucose DMEM (Gibco, USA) with 10 v v^−1^% FBS and 1% penicillin-streptomycin. hBMSCs were cultured using ɑ-MEM (Gibco, USA) with 10 v v^−1^% FBS and 1 % penicillin-streptomycin. The culture media were refreshed every 2–3 days, and all cultures were passaged at 70–90% confluence using trypsin-EDTA. To evaluate the regulatory effects of composite membranes on the migration of RSCs, HUVECs, and hBMSCs under inflammatory conditions, a Transwell co-culture model was established. LPS-stimulated RAW264.7 macrophages and various composite membranes were placed in the lower chamber, while RSCs, HUVECs, or hBMSCs were respectively seeded in the upper chamber. After 48 h of co-culture, non-migrated cells were removed from the upper surface of the membranes. Subsequently, the membranes were stained with crystal violet after being fixed and washed. Migrated cells on the membranes were observed using an inverted microscope. The number and area of migrated cells were quantified using the ImageJ. To investigate cells adhesion, RSCs, HUVECs, and hBMSCs were respectively cultured on the porous layers of the membranes for certain periods. At each time point, IF staining-Alexa Fluor 555-phalloidin (CA1610, Solarbio) was performed to analyze the morphologies of cells adhered on composite membranes. To evaluate the viability and proliferation of RSCs, HUVECs, and hBMSCs co-cultured with different membranes, Live/Dead staining assay and CCK-8 assay (CK04-500, Dojindo) were performed at each time point.

### In vitro neurovascularization and osteogenesis regulations

RSCs (1 × 10^4^ cells well^−^^1^) were seeded into glass-bottom confocal dishes (Corning, USA) and co-cultured with composite membranes in serum-free medium for 24 h. Complete medium containing 10 % FBS served as the positive control. After being fixed with 4% paraformaldehyde and permeabilized with 0.1 v v^−^^1^% Triton X-100 (Sigma-Aldrich, USA), the cells were stained with Phalloidin (CA1610, Solarbio) and DAPI (C1006, Beyotime) to visualize their cytoskeletons and nuclei, respectively. Images were acquired using a confocal laser scanning microscope (LSM 900, Zeiss, Germany). ImageJ was used to measure the maximum extension length per branched cell (µm cell^−^^1^) and the ratio of branched cells. PC12 cells (1 × 10⁴ cells per well) were co-cultured with composite membranes in 24-well plates under standard conditions for 24 h. Cells morphologies and neurite outgrowth were observed using a phase-contrast inverted microscope (CKX53, Olympus, Japan). Neurite lengths of neurite-bearing cells were quantified using ImageJ. For tube formation, HUVECs (1.3 × 10⁴ cells well^−^^1^) were seeded onto Matrigel-coated 96-well plates (Corning, USA) and co-cultured with membranes for 8 h. Images of capillary-like networks were recorded under an inverted microscope (CKX53, Olympus, Japan) and then quantified using the Angiogenesis Analyzer plugin in ImageJ. For scratch wound assays, HUVECs (3 × 10⁵ cells well^−1^) were cultured in 12-well plates to ~70% confluency and then a sterile pipette tip was used to create a linear scratch on the plates. After that, HUVECs were subsequently incubated in the low-serum (2% FBS) medium containing membranes’ extracts for 16 h. IF staining experiments were performed as described in the section of In vitro immunomodulation. Primary anti-GAP43 (Beyotime, AG1988), NGF (Invitrogen, SI79-01), and VEGF (Invitrogen, JH121) were co-incubated with the cells overnight at 4 °C. After being washed three times with PBS solution, the cells were incubated with the corresponding secondary antibody diluent at 37 °C for 1 hour. Next, the cells nuclei were further stained with DAPI (C1006, Beyotime) for 5 minutes. Images were captured using a confocal laser microscope (LSM 900, Zeiss). The average fluorescence intensity for each group was analyzed using ImageJ software. To investigate osteogenic regulation of the membranes in vitro, hBMSCs were co-cultured with each membrane in osteogenic medium for 7 and 14 days followed by being stained respectively using a BCIP/NBT ALP kit (Beyotime) and 1% ARS solution for evaluate the ALP activity and mineralization of the cells. qPCR experiments on RSCs, HUVECs, and hBMSCs used same method as description above. Cells viability and proliferation of hPDSCs (iCell, China) co-cultured with the membranes were also evaluated through CCK-8 assay (CK04-500, Dojindo) and Live/Dead staining (Calcein-AM/PI, 1:1000; Beyotime). The osteogenic differentiation of hPDSCs co-cultured with the membranes was measured by ALP (7 days) and Alizarin Red S (14 days) staining methods as above description.

### In vivo experiments

In vivo *rat* critical size cranial defect experimental procedures and protocols were approved by the Ethics Review Committee of the Shenzhen Institutes of Advanced Technology (IACUC Number: SIAT-IACUC-240326-YGS-ZYC-A2527). In vivo antibacterial experiments were carried out according to the institutional guidelines and approved by Animal Care and Use Committee (IACUC) in Shanghai Yishang Biotechnology Co., Ltd (IACUC-2025-1-037). For in vivo antibacterial experiments, a total of 6 *Sprague-Dawley (SD) rats* (male, 7-8 weeks) were randomly divided into CM group or CSO_1_M_3_ group. The surfaces of the barrier layers of the samples were separately applied dropwise with 10 µL of *S. aureus* or *E. coli* suspension (1×10^8^ CFU mL^−1^), and then implanted subcutaneously on the dorsal side of rats. After 7 days, the animals were euthanized with the following specimens harvested: (1) soft tissues above the membranes, (2) the membranes, and (3) soft tissues below the membranes. Excised tissues were mechanically homogenized in sterile PBS (2 mL per sample) with a handheld homogenizer (TissueRuptor II, Qiagen). To recover bacteria adherent to the implanted membranes, each membrane was sonicated for 5 minutes in 1 mL PBS in a water-bath sonicator (Branson 2510). Homogenates and sonicates were serially diluted 5-fold, and then 100 µL of each dilution was spread onto tryptic soy agar plates (in duplicate) at 37 °C for 24 h. CFUs were enumerated with the ImageJ software (National Institutes of Health, USA), recorded as CFU per gram of tissues. Among them, plates with no visible bacterial colonies were recorded as 0. For histological examinations, collected tissues were fixed overnight in 4% paraformaldehyde at 4 °C and then dehydrated through graded ethanol. After that, the samples were embedded in paraffin followed by being cut into sections with a thickness of ~5 µm. After being stained, the sections were evaluated by a light microscopy (Olympus IX71, Japan).

In vivo neurovascularization and osteogenesis regulations were evaluated using a critical-sized cranial defect model (~5 mm diameter). A total of 64 *SD rats* (male, 7-8 weeks) were randomly divided into control group (defect only), CM group, CSO_1_ group, or CSO_1_M_3_ group. Specifically, rats were anaesthetized with isoflurane gas. Then the circular critical-sized cranial defects with diameters of ~5 mm were prepared, followed by removal of the periosteum above the defects. The membranes with diameters of ~6 mm were placed over the defective sites and then properly sutured. All experimental rats received intraperitoneal injections of penicillin for 3 days to prevent infection. After 1 week, blood of the rats was collected for cytokine array analysis (RayBiotech AAR-CYT-3-2) to measure the concentrations of inflammatory and anti-inflammatory factors. After 2 weeks, the newly formed blood vessels and blood perfusion were evaluated using the RFLSI Laser Speckle Imaging System (RFLSI-ZW) from Shenzhen Ruiward Life Technology Co., Ltd. After 4 weeks, RNA-seq (NovaSeq 6000, Illumina) was performed using collected tissues from the CSO_1_M_3_ and control groups. To identify differentially expressed mRNAs, paired comparisons were performed using the ‘edgeR’ R package based on fold changes > 2 or < 0.5. Furthermore, GO and KEGG enrichment analysis were evaluated using the OmicStudio tool, respectively. After 4 and 8 weeks, the experimental rats were respectively euthanized. The *rat* skull samples were harvested and then scanned using a micro-computed tomography (Skyscan 1176, Bruker, Belgium) scanner. 3D reconstruction and visualization of the samples were performed using an NRecon software (Bruker, Germany) and a CTvox software (Bruker, Germany), respectively. The region of interest (ROI) was defined as a cylindrical space (~5 mm of diameter, ~1 mm of height). The percentage of BV/TV and BMD were measured using a CTAn software (Bruker, Germany). After decalcification, samples were embedded in paraffin and then cut into sections with a thickness of 4 µm. At each time point, immunofluorescent staining images of the tissue sections were performed using the same method as described in the section of In vitro immunomodulation. The primary antibodies included CD86 (Biorbyt, orb500820-cf594), CD163 (Biorbyt, orb612214), CD68 (Abcam, ab125212), NGF (Invitrogen, SI79-01), VEGF (Invitrogen, JH121), S100-β (Proteintech,15146-1-AP), Emcn (Bioss, bs-5884R-PE), CGRP (Beyotime, AF6495), p-PI3K (Thermo Fisher, PA5-104853), p-Akt (Sigma-Aldrich, WH0000207M3), Runx2 (Thermo Fisher, H00000860-M01), OCN (Proteintech, 23418-1-AP), NF200 (Thermo Fisher, 711025), Osterix (Invitrogen, PSH07-29), CD31 (Thermo Fisher, 14-0311-82) and Tubb3 (Proteintech, 66375-1-lg). In addition, tissue sections were stained by the Masson trichrome staining kit (Beyotime, C0189S) and eosin (Beyotime, C0105) for histological evaluations.

### Single-cell RNA sequencing experiments

A *SD rat* cranial critical-sized defect model was established, and total 12 *SD rats* (male, 7-8 weeks) were randomly divided into the control and CSO_1_M_3_ groups. Newly formed tissues from both groups were harvested at 1 and 2 weeks post-surgery. The surrounding tissues were meticulously removed to eliminate interference from non-target cell populations. The samples were mechanically dissociated into fragments and enzymatically digested in a mixture of 0.1% Type I collagenase (Gibco, 17100017) and 0.1% Type II collagenase (Gibco, 17101015) at 37 °C for 40 min. The resulting cell suspension was filtered through a 40 µm nylon mesh and centrifuged with a speed of 300 g. After removing erythrocytes using Red Blood Cell Lysis Solution (Miltenyi Biotec, 130-094-183), cell concentration and viability (> 85%) were determined using a Luna™ Automated Cell Counter (Logos Biosystems, Anyang, South Korea). The single-cell suspension was adjusted to 700–1200 cells µL^−1^. Cell capture was performed according to the manufacturer’s instructions for the 10× Genomics Chromium Next GEM Single Cell 3ʹ Reagent Kits v3.1 (USA, PN-1000268). The final libraries were sequenced on the BGI DNBSEQ-T7 PE100 (MGI Tech Co., Ltd., Shenzhen, China) in paired-end 100 bp mode.

The raw FASTQ files were processed using Cell Ranger (v9.0.0) for alignment against the *rat* reference genome (Rnor_6.0) and generation of the feature-barcode matrix^64^. Quality control was conducted using the Seurat R package (v5.0); high-quality cells were retained based on the criteria of nFeature_RNA (200–5000) and mitochondrial gene percentage (< 10%)^65^. Principal Component Analysis (PCA) was applied for dimension reduction, and the Harmony algorithm was employed to integrate samples and remove batch effects. The first 30 principal components were visualized via t-SNE, and cell clustering was performed using the FindClusters function based on the SNN algorithm. For macrophage and osteoblast sub-clusters, coordinates were extracted for secondary dimension reduction and clustering. Sub-populations were identified based on differentially expressed genes (DEGs) and canonical markers. GO enrichment analysis was performed using clusterProfiler (v4.0) to investigate the dynamic evolution of biological processes (BP) across different time points and experimental groups^66^. CytoTRACE (v0.3.3) was utilized to predict differentiation potential and the origin of the osteogenic lineage^67^. Subsequently, Monocle 3 (v1.3.1) was used to construct pseudotime trajectories by learning the manifold structure, thereby revealing the dynamic differentiation landscape and lineage progression during the bone regeneration process^68^. Finally, intercellular communication was inferred using CellChat (v1.6.1) based on the ligand-receptor interaction database^69^.

### Statistical analysis

One-way ANOVA followed by Tukey’s post hoc test and two-tailed Student’s *t*-test were used for statistical analysis, respectively. Unless otherwise specified, all values represent the mean and standard deviation (*n*  =  3 independent samples per group). No other adjustments were made for multiple comparisons. *p* < 0.05 was considered statistically significant differences between the compared group. Quantitative results were analyzed using the software including ImageJ, GraphPad Prism 8, Microsoft Excel 2013, and OriginPro 2022.

### Reporting summary

Further information on research design is available in the Nature Portfolio Reporting Summary linked to this article.

## Supplementary information

Supplementary Information

Description of Additional Supplementary File

Supplementary Movie 1

Reporting Summary

Transparent Peer Review file

## Source data

Source Data

## Data Availability

All data generated in this study are provided in the main text and Supplementary Information. The RNA-seq and scRNA-seq raw data have been deposited respectively in the Genome Sequence Archive in National Genomics Data Center, China National Center for Bioinformation/Beijing Institute of Genomics, Chinese Academy of Sciences under the accession code “CRA035122 [https://ngdc.cncb.ac.cn/gsa/browse/CRA035122]” and under the accession code “CRA040859 [https://ngdc.cncb.ac.cn/gsa/browse/CRA040859]”. Any additional requests for information can be directed to and will be fulfilled by, the corresponding authors. Source data are provided with this paper.
