# Author Correction: Unified bilayer membranes with mechanically reinforced interface for stage-adaptive bone regeneration

**DOI:** 10.1038/s41467-026-78171-y

**Published:** 2026-09-28

**Authors:** Yuhan Du, Yangyi Nie, Zhiheng Luo, Junjie Deng, Zili Xu, Yuyang Zhang, Chenyu Gao, Yujie Liu, Xiangcheng Lei, Jing Long, Cairong Li, Zhenyu Yao, Wei Zhang, Ling Qin, Yuanchi Zhang, Yuxiao Lai

**Affiliations:** 1 Centre for Translational Medicine Research and Development, Shenzhen Institutes of Advanced Technology, Chinese Academy of Sciences, Shenzhen, China; 2 University of Chinese Academy of Sciences, Beijing, China; 3 Tianjin Hospital, Tianjin University, Tianjin, China; 4 The Brain Cognition and Brain Disease institute of Shenzhen Institutes of Advanced Technology, Chinese Academy of Sciences, Shenzhen, China; 5 Faculty of Health Sciences, University of Macau, Macau, China; 6 Department of Orthopaedics and Traumatology, The Chinese University of Hong Kong, Hong Kong, China; 7 Key Laboratory of Biomedical Imaging Science and System, Chinese Academy of Sciences, Shenzhen, China; 8 Guangdong Engineering Laboratory of Biomaterials Additive Manufacturing, Shenzhen, China; 9 National Innovation Center for Advanced Medical Devices, Shenzhen, China

**Keywords:** Fracture repair, Biomedical materials, Biomedical engineering, Biomedical materials

Correction to: *Nature Communications* https://doi.org/10.1038/s41467-026-74932-x, published online 30 June 2026

In the version of this article initially published, in the Fig. 2d Source Data and associated figure panel, the Raman intensity data for the CS membrane were all inadvertent duplicate values. The correct Source Data and Fig. 2d panel have been updated in the HTML and PDF versions of the article. For comparison, the original Fig. 2 is available as Supplementary Information accompanying this amendment.

## Supplementary information

Original Fig. 2

