# Supplementary material for: Unified bilayer membranes with mechanically reinforced interface for stage-adaptive bone regeneration
Source: Nat Commun. 2026 Jun 30;17:8193. doi: 10.1038/s41467-026-74932-x (PMC13462358; doi:10.1038/s41467-026-74932-x)
Supplement: Supplementary file 1 [file 41467_2026_74932_MOESM1_ESM.pdf]

Supplementary Information

Unified Bilayer Membranes with Mechanically Reinforced Interface for Stage-adaptive Bone Regeneration

Yuhan Du[1, 2], Yangyi Nie[1], Zhiheng Luo[3], Junjie Deng[1], Zili Xu[1], Yuyang Zhang[1, 2], Chenyu Gao[4], Yujie Liu[1], Xiangcheng Lei[1, 5], Jing Long[1, 2], Cairong Li[1, 2], Zhenyu Yao[1, 2], Wei Zhang[1, 2], Ling Qin[1, 6], Yuanchi Zhang[1, 2*], Yuxiao Lai[1, 2, 7, 8, 9*]

[1] Centre for Translational Medicine Research and Development, Shenzhen Institutes of Advanced Technology, Chinese Academy of Sciences, Shenzhen, 518055, China.

[2] University of Chinese Academy of Sciences, Beijing, 100433, China.

[3] Tianjin Hospital, Tianjin University, Tianjin 300211, China.

[4] The Brain Cognition and Brain Disease institute of Shenzhen Institutes of Advanced Technology, Chinese Academy of Sciences, Shenzhen, 518055, China.

[5] Faculty of Health Sciences, University of Macau, Macau, 999078, China.

[6] Department of Orthopaedics and Traumatology, The Chinese University of Hong Kong, Hong Kong, 999077, China.

[7] Key Laboratory of Biomedical Imaging Science and System, Chinese Academy of Sciences, Shenzhen, 518055, China.

[8] Guangdong Engineering Laboratory of Biomaterials Additive Manufacturing, Shenzhen, 518055, China.

[9] National Innovation Center for Advanced Medical Devices, Shenzhen, 518131, China

*Corresponding Author: Yuanchi Zhang, Yuxiao Lai

E-mail: zhangyc@siat.ac.cn, yx.lai@siat.ac.cn,

This PDF file includes:

Supplementary Figs. 1 to 49
Supplementary Tables 1 and 2
References

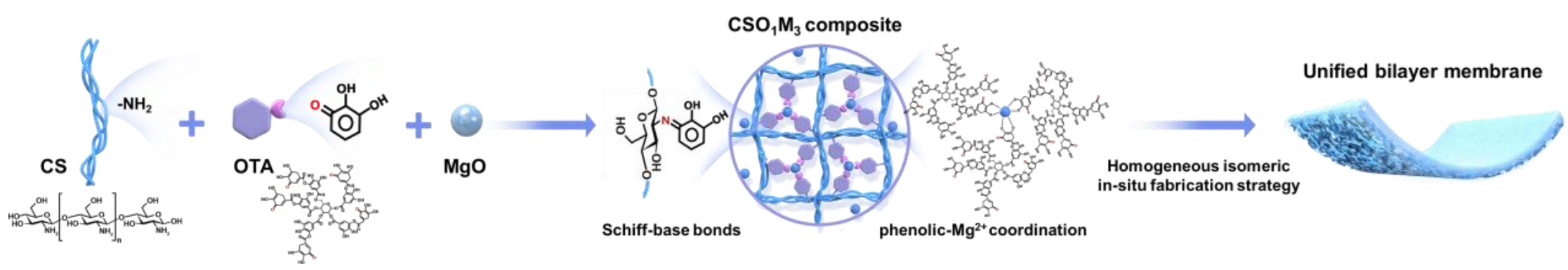

Supplementary Fig. 1. Schematic illustration of preparation of the unified bilayer membranes.

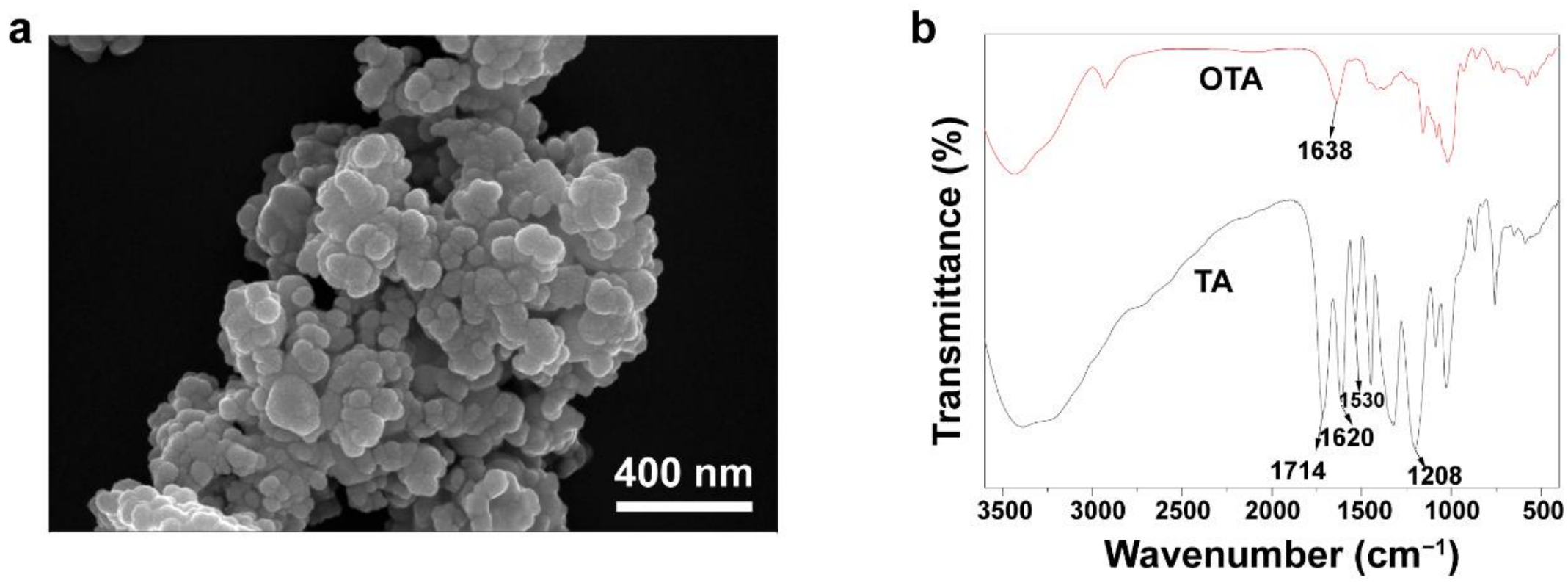

Supplementary Fig. 2. Characterization of magnesium oxide (MgO) nanoparticles and oxidized tannic acid (OTA). a Scanning electron microscopy (SEM) image of MgO nanoparticles. The images were repeated at least three times with consistent results. **b** FTIR spectra of tannic acid (TA) and oxidized tannic acid (OTA).

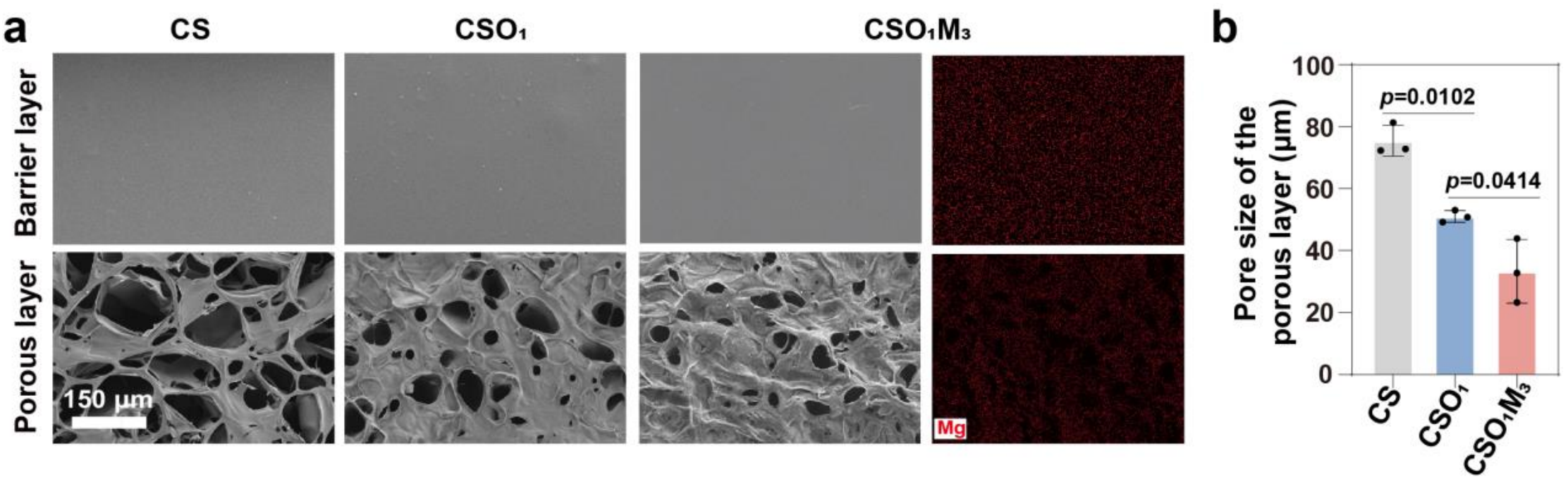

Supplementary Fig. 3. Surface morphologies and pore sizes of the chitosan (CS), CS-OTA (CSO_1), CS-OTA_1-MgO_3 (CSO_1M_3) membranes. a SEM images of the barrier layer and porous layer of various membranes. Energy dispersive spectrometry (EDS) images showed a uniform distribution of Mg^{2+} on the surface of barrier layer and porous layer of the CSO_1M_3 membranes. The images were repeated at least three times with consistent results. **b** Quantitative analysis of pore sizes based on SEM images. Values in (b) represent the mean and standard deviation ($n = 3$ independent samples per group). Statistical analyses were performed using one-way ANOVA followed by Tukey's post hoc test. $p < 0.05$ was considered statistically significant differences between the compared groups.

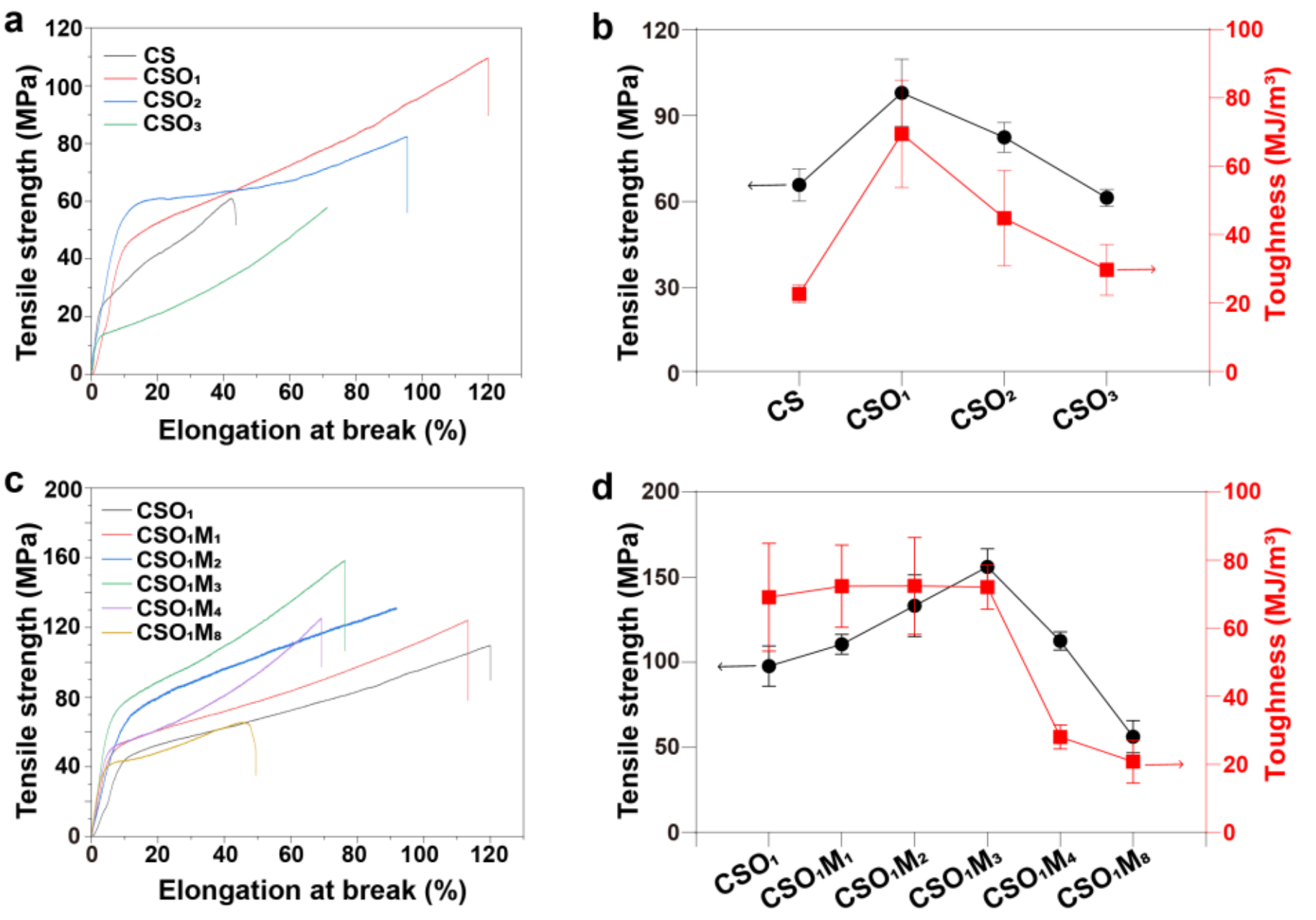

Supplementary Fig. 4. Mechanical properties of various composite membranes under dry conditions. **a** Stress-strain curves of pristine CS and CS incorporated with 1-3wt% OTA membranes. CSO$_1$: CS incorporated with 1wt% OTA. CSO$_2$: CS incorporated with 2wt% OTA. CSO$_3$: CS incorporated with 3wt% OTA. **b** Tensile strength and toughness derived from the stress-strain curves in **(a)**. **c** Tensile stress-strain curves of the CSO$_1$ membranes with 1-8 wt% of MgO nanoparticles. CSO$_1$M$_1$: CSO$_1$ with 1wt% MgO. CSO$_1$M$_2$: CSO$_1$ with 2wt% MgO. CSO$_1$M$_3$: CSO$_1$ with 3wt% MgO. CSO$_1$M$_4$: CSO$_4$ with 4wt% MgO. CSO$_1$M$_8$: CSO$_1$ with 8wt% MgO. **d** Tensile strength and toughness derived from the stress-strain curves in (**c**). Values in (b) and (d) represent the mean and standard deviation ($n = 3$ independent samples per group).

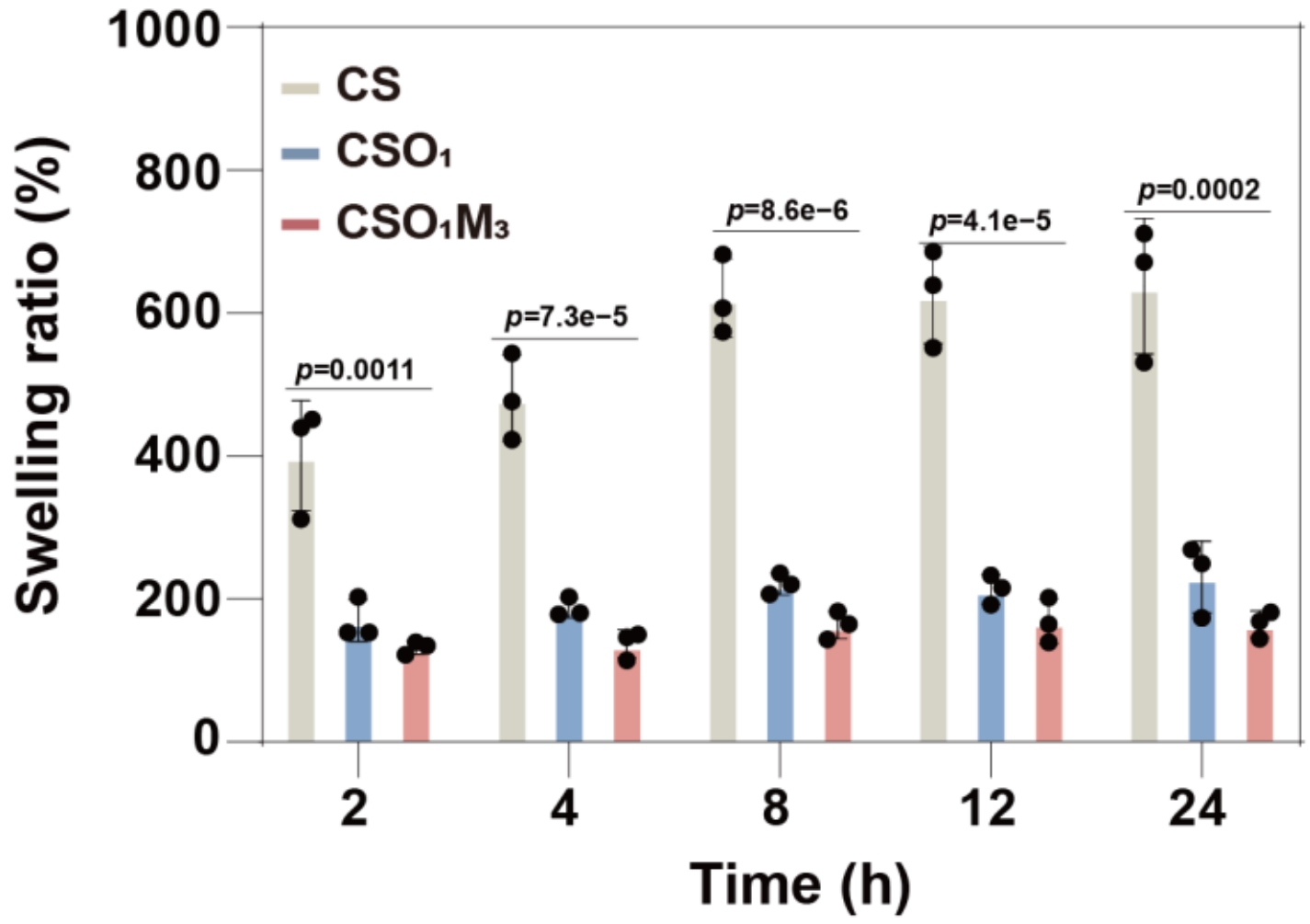

Supplementary Fig. 5. Quantitative analysis of the swelling ratios of CS, CSO_1, and CSO_1M_3 membranes over time (2, 4, 8, 12, and 24 h). The CSO_1 and CSO_1M_3 membranes exhibited significantly reduced swelling ratios compared to the pristine CS membranes, indicating enhanced network stability. Values represent the mean and standard deviation ($n = 3$ independent samples per group). Statistical analyses were performed using one-way ANOVA followed by Tukey's post hoc test. $p < 0.05$ was considered statistically significant differences between the compared groups.

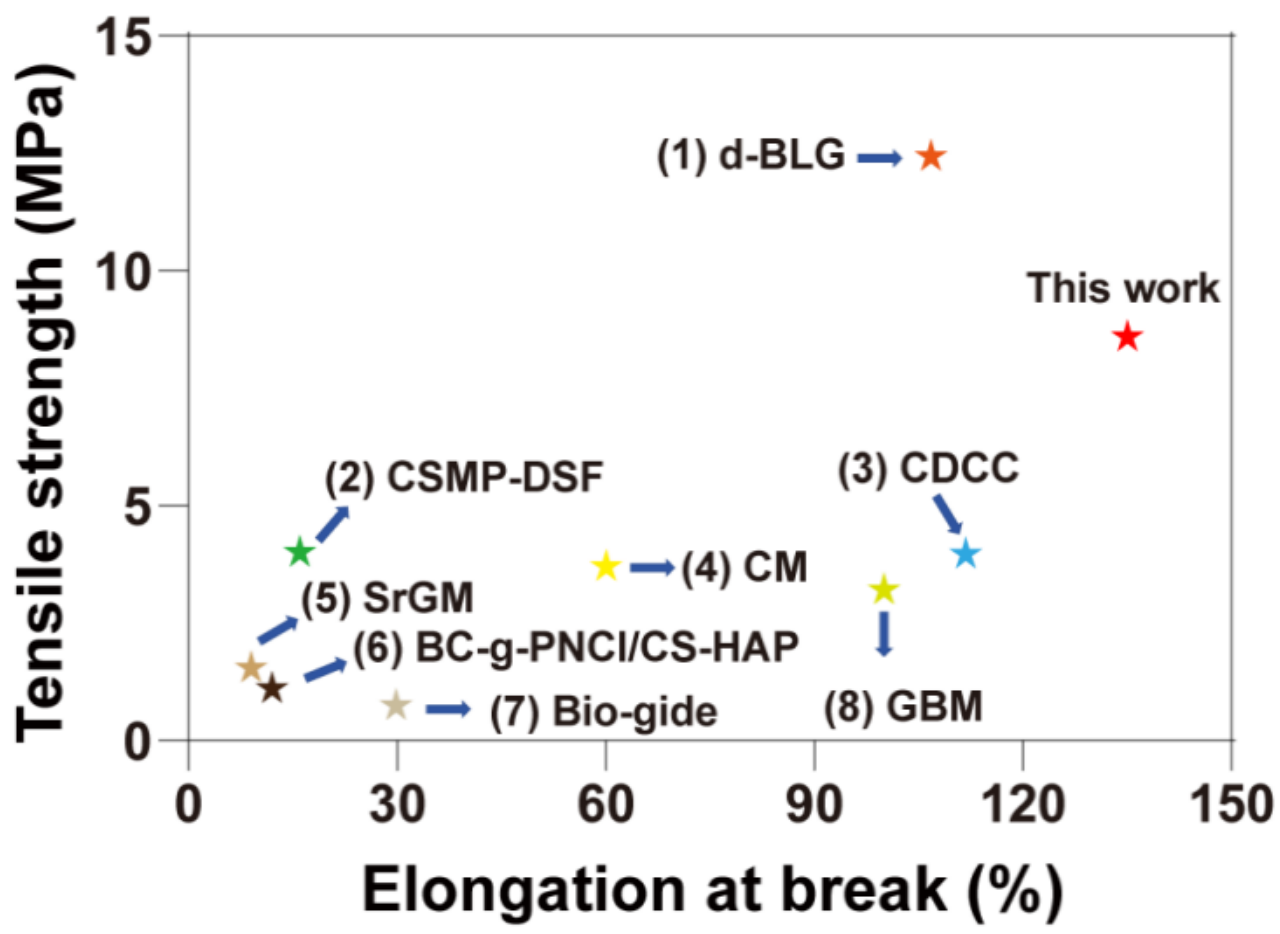

Supplementary Fig. 6. Comparative evaluation of the comprehensive mechanical properties of the CSO_1M_3 membranes and reported double-layer membranes[1-8].

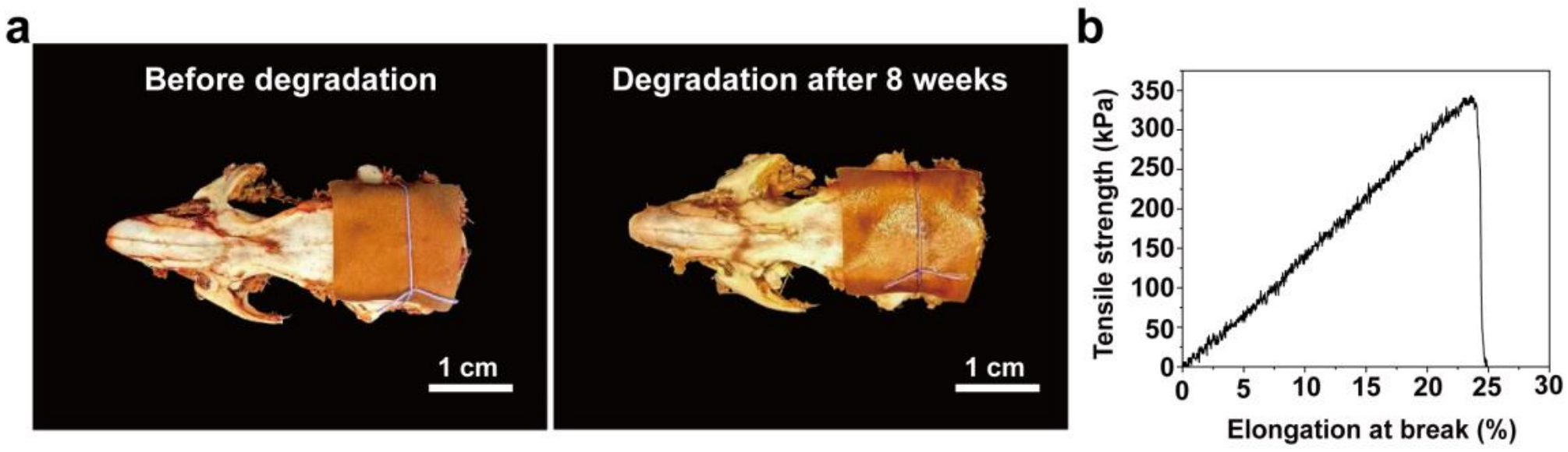

Supplementary Fig. 7. The qualitative (a) and quantitative (b) analysis of the structural stability of the CSO_1M_3 membrane after degradation for 8 weeks. $n = 3$ independent samples per group.

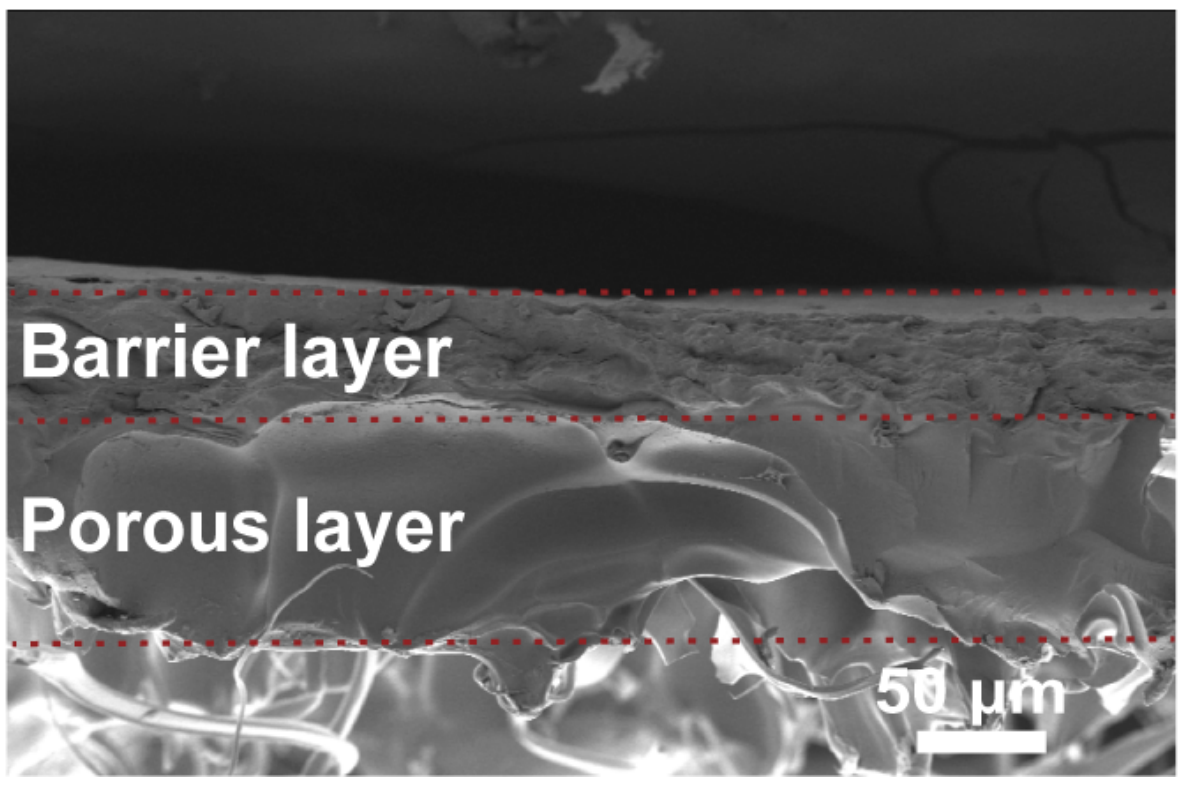

Supplementary Fig. 8. Cross-sectional morphology of the CSO_1M_3 membranes after 56 days of in vitro degradation in lysozyme-containing PBS solution. The images were repeated at least three times with consistent results.

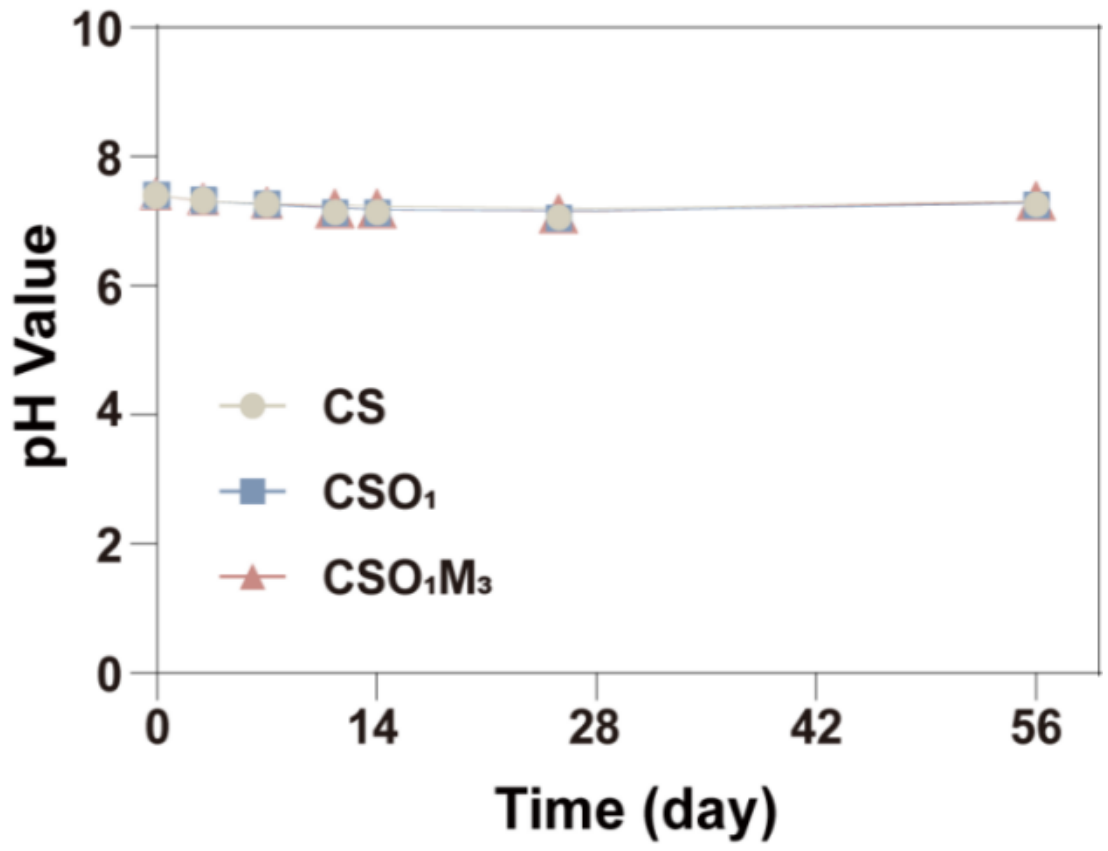

Supplementary Fig. 9. Time-dependent pH values measured at designated time points during in vitro degradation of the CS, CSO_1, and CSO_1M_3 membranes in lysozyme-containing PBS over 56 days. Values represent the mean and standard deviation ($n = 3$ independent samples per group).

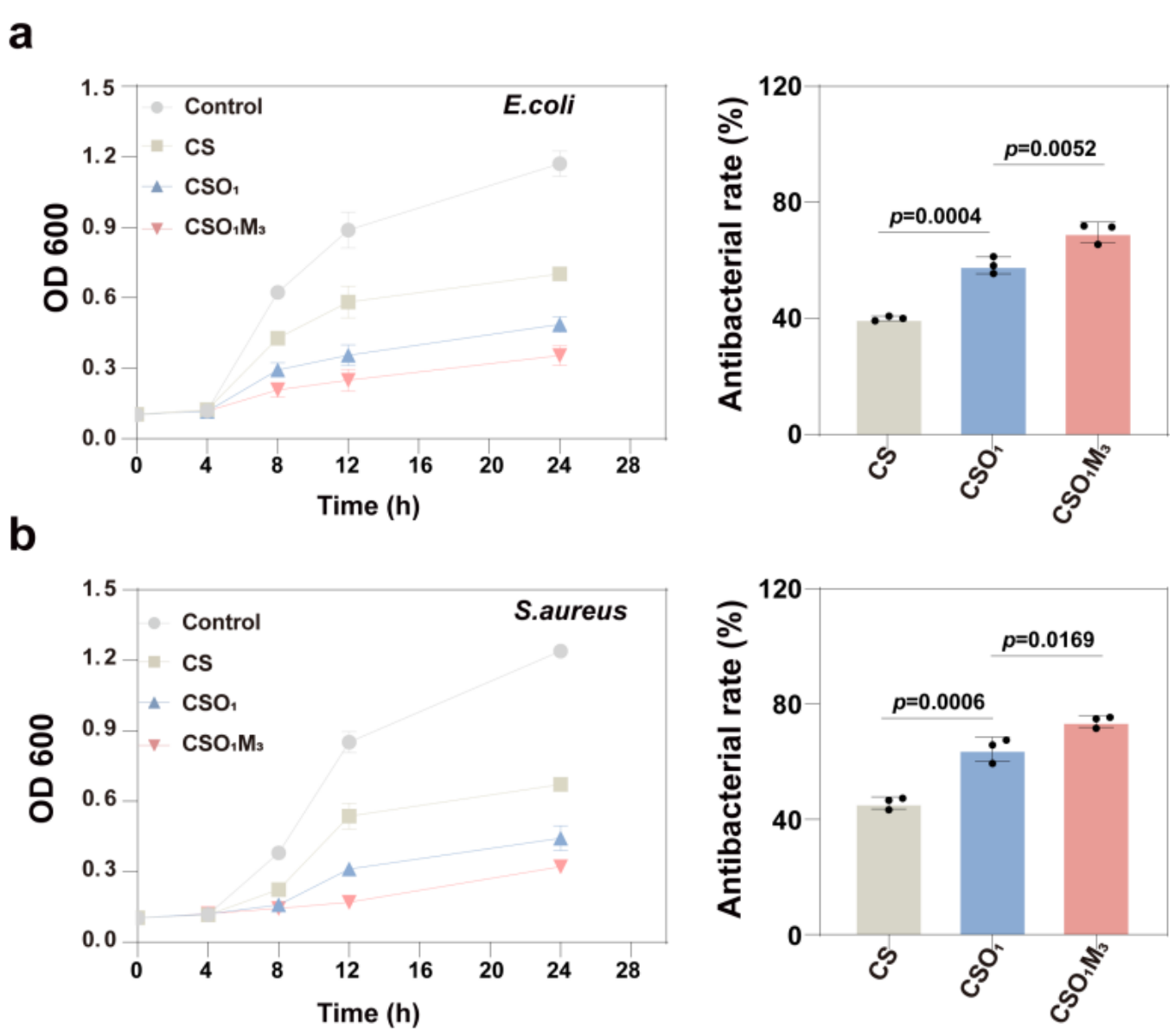

Supplementary Fig. 10. Antibacterial properties of the CS, CSO_1, and CSO_1M_3 membranes. a Bacterial growth curves and antimicrobial rate against Escherichia coli (*E. coli*). **b** Bacterial growth curves and antimicrobial rate against Staphylococcus aureus (*S. aureus*). Values in (a) and (b) represent the mean and standard deviation ($n = 3$ independent samples per group). Statistical analyses were performed using one-way ANOVA followed by Tukey's post hoc test. $p < 0.05$ was considered statistically significant differences between the compared groups.

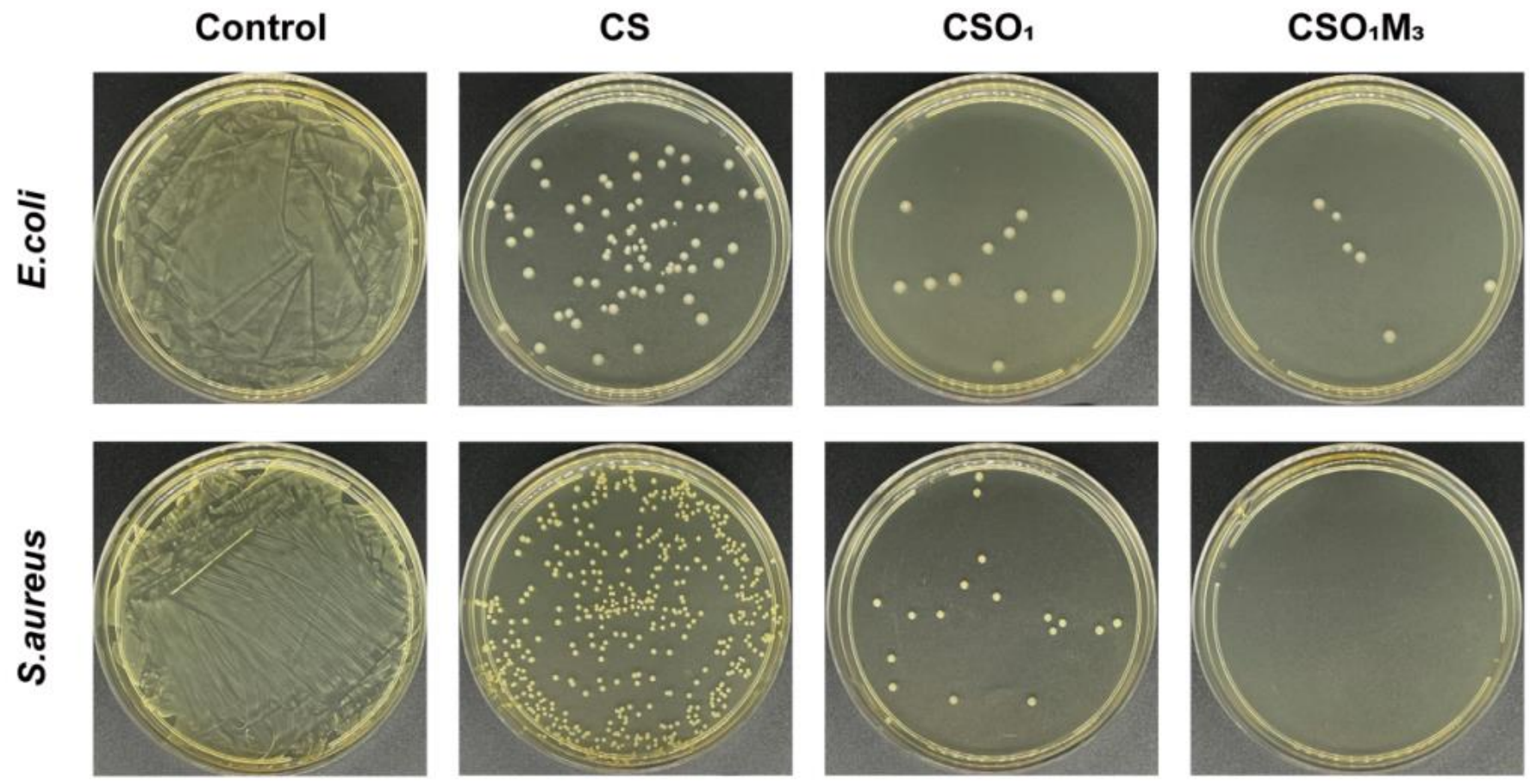

Supplementary Fig. 11. Representative images of *E. coli* and *S. aureus* cultured on Luria-Bertani (LB) agar plates for 24 h using the 3-days co-culture suspensions in various group ($n = 3$ independent samples per group).

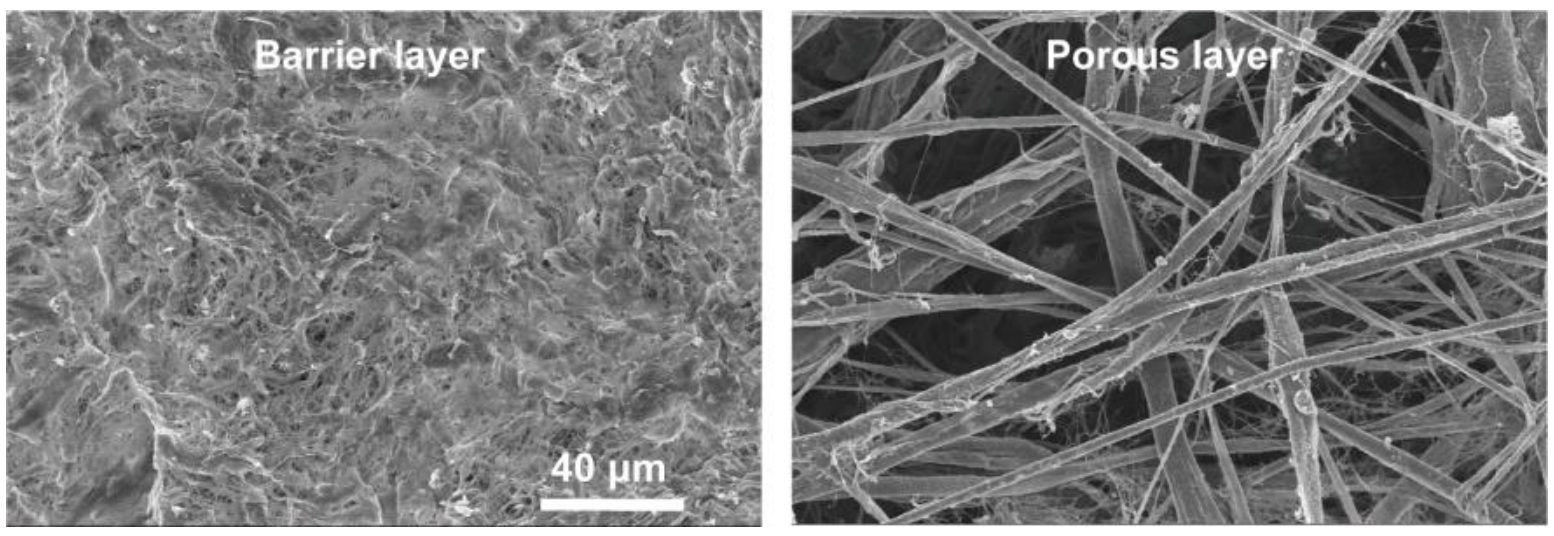

Supplementary Fig. 12. SEM images of the dense barrier layer and the porous layer of the commercial Bio-Gide membranes (CM). The images were repeated at least three times with consistent results.

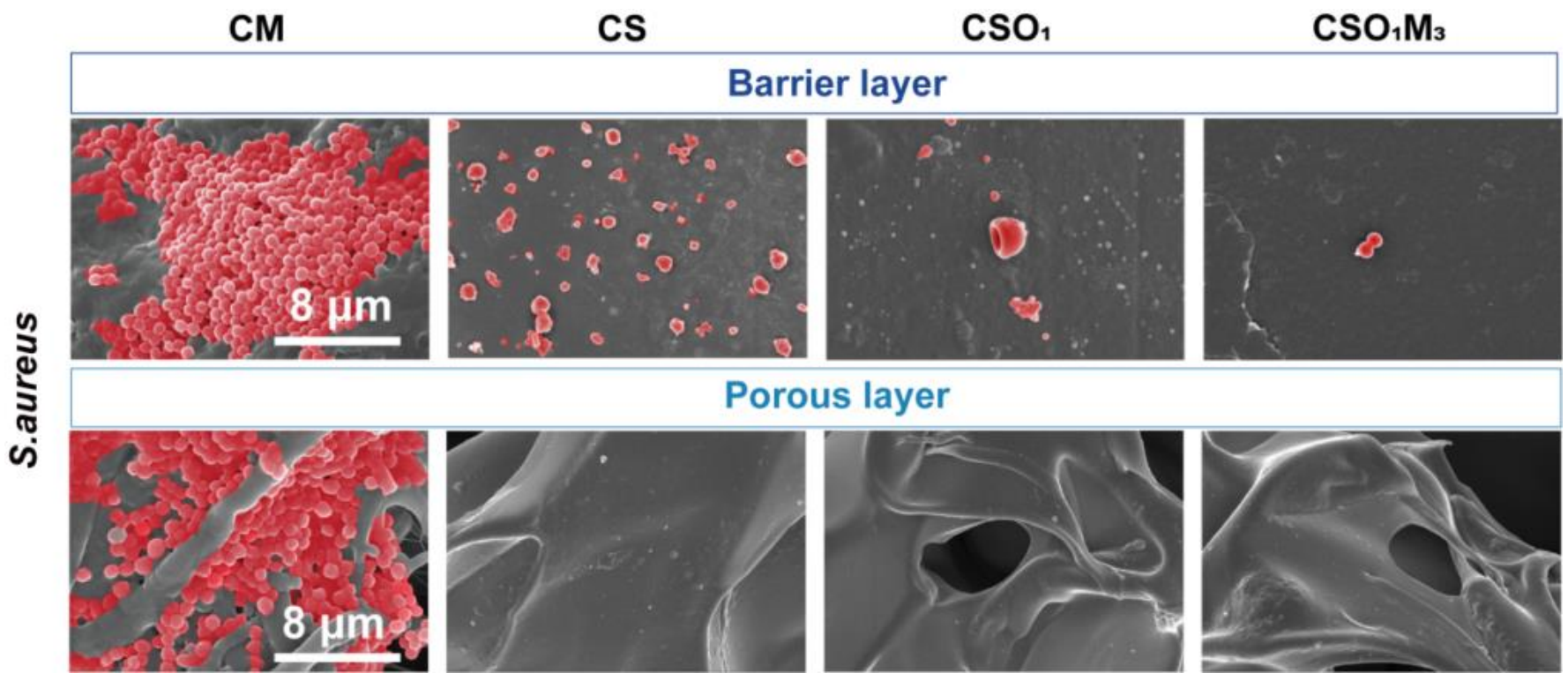

Supplementary Fig. 13. SEM images of the barrier layers and the porous layers in various groups after *S. aureus* penetration for 7 days. The images were repeated at least three times with consistent results.

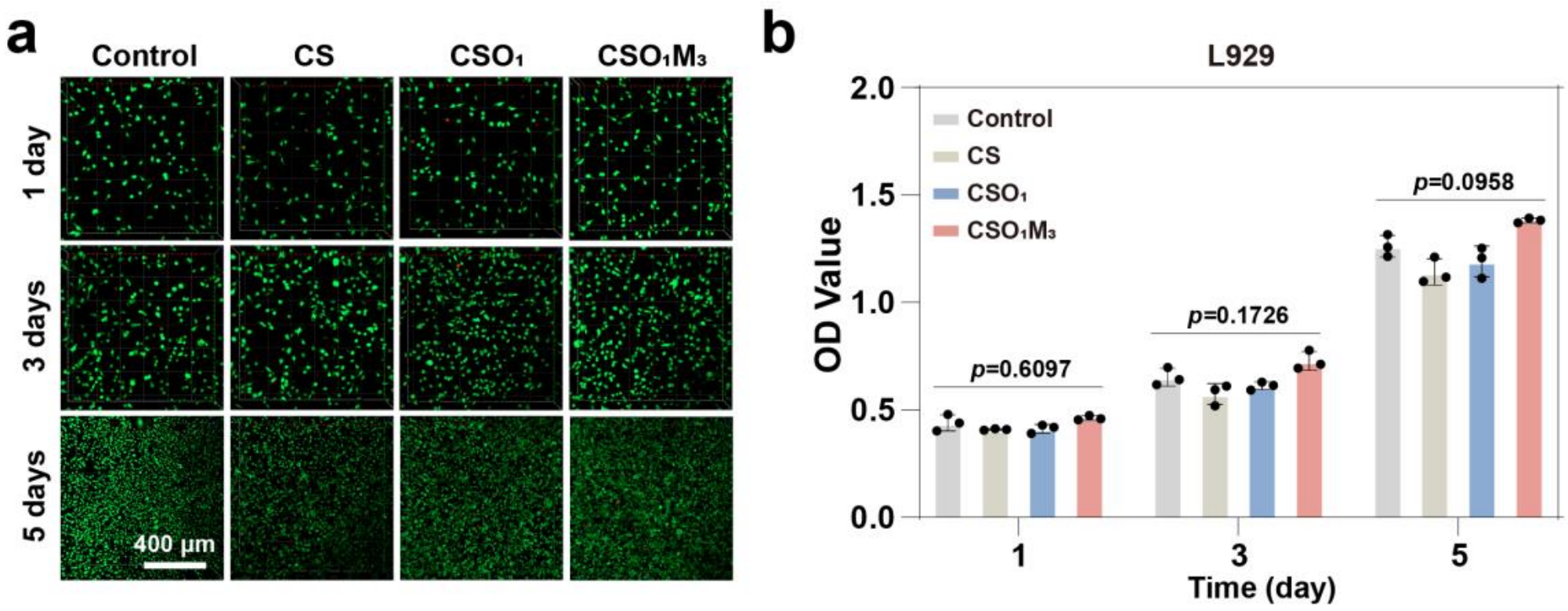

Supplementary Fig. 14. Cytocompatibility of the CS, CSO_1, and CSO_1M_3 membranes with L929 cells. a 3D confocal fluorescence images of live/dead-stained L929 cells cultured on the barrier layers of different membranes for 1, 3, and 5 days. **b** Quantitative CCK-8 results. Values in (b) represent the mean and standard deviation ($n = 3$ independent samples per group). Statistical analyses were performed using one-way ANOVA followed by Tukey's post hoc test. $p < 0.05$ was considered statistically significant differences between the compared groups.

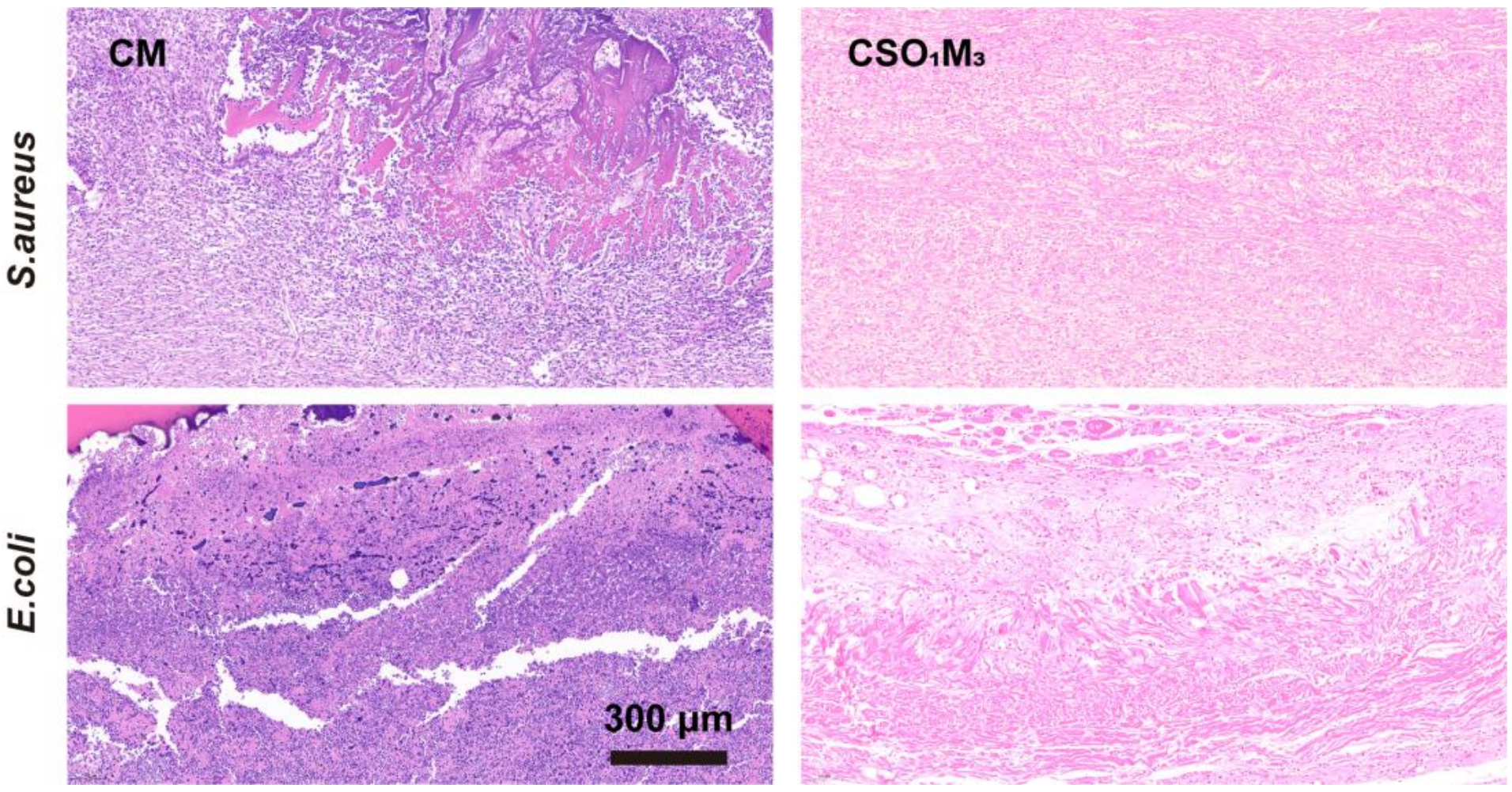

Supplementary Fig. 15. Hematoxylin and eosin (H&E) staining images of subcutaneous tissues of the *rat* models after implanting the membranes with various bacteria for 7 days. The images were repeated at least three times with consistent results.

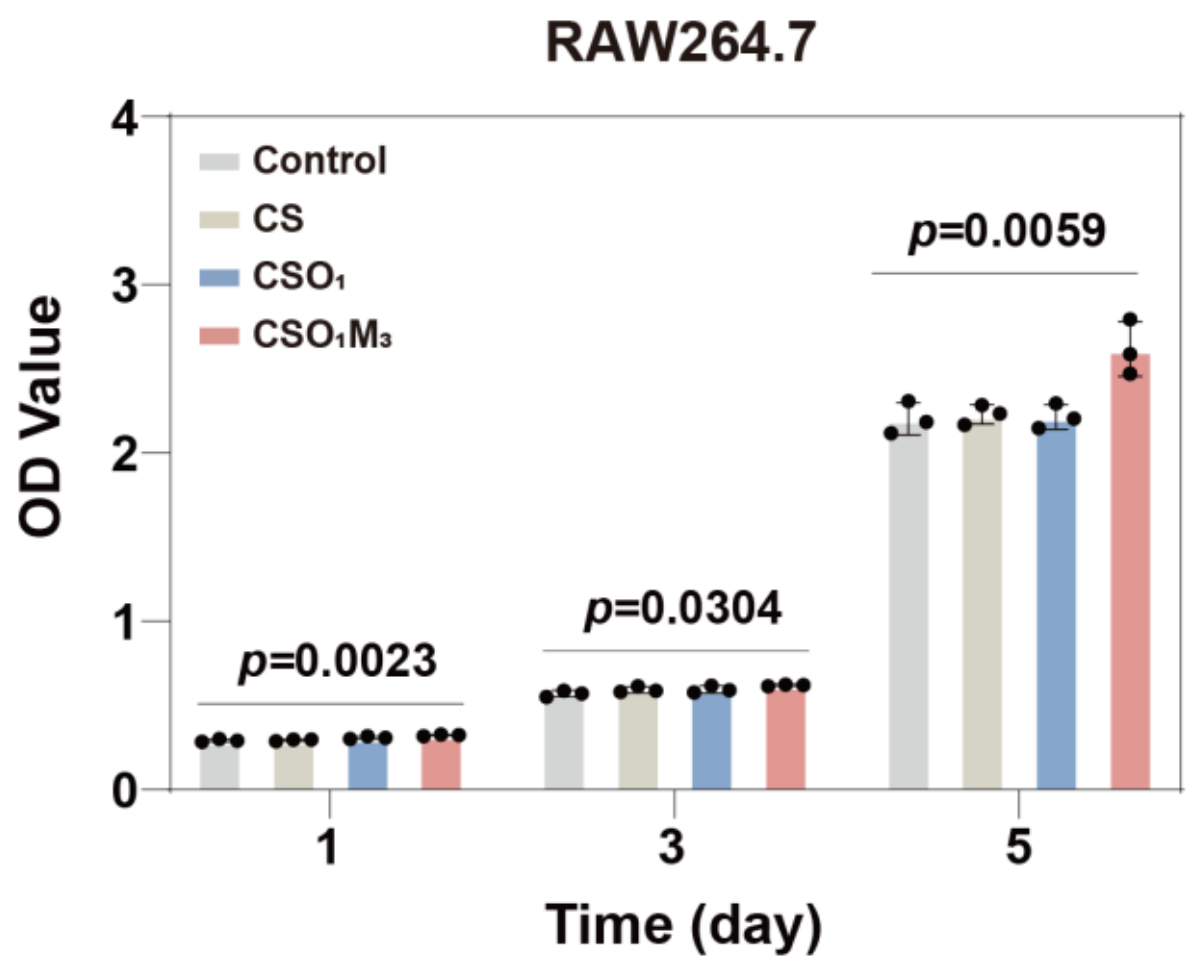

Supplementary Fig. 16. The cytocompatibility of RAW264.7 macrophages co-cultured with different membranes for 1, 3, and 5 days. Values represent the mean and standard deviation ($n = 3$ independent samples per group). Statistical analyses were performed using one-way ANOVA followed by Tukey's post hoc test. $p < 0.05$ was considered statistically significant differences between the compared groups.

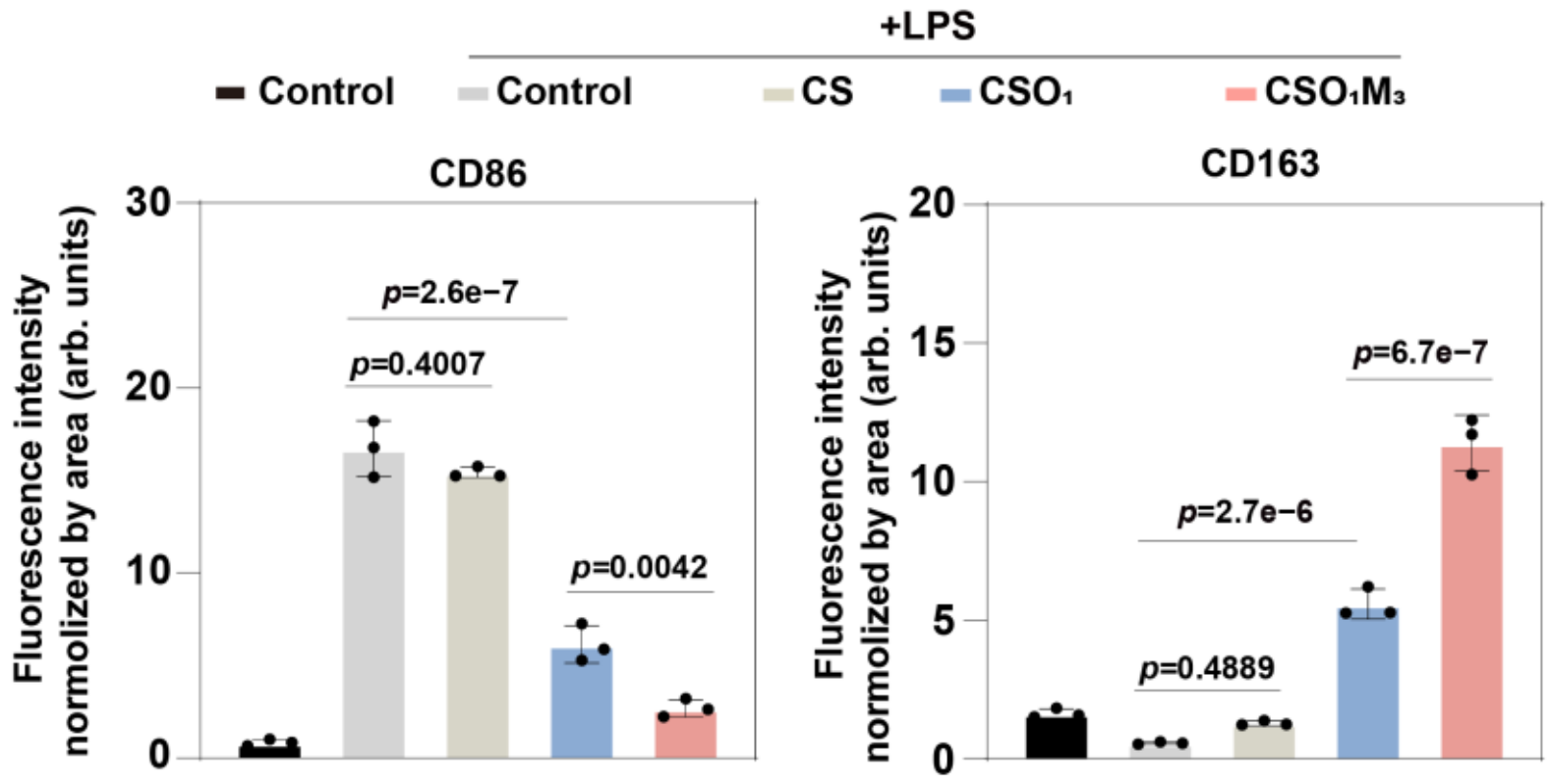

Supplementary Fig. 17. Quantitative analysis of the immunofluorescence (IF) staining images for CD86 (M1 marker) and CD163 (M2 marker) expression after 48 hours of co-culture with different composite membranes. Values represent the mean and standard deviation ($n = 3$ independent samples per group). Statistical analyses were performed using one-way ANOVA followed by Tukey's post hoc test. $p < 0.05$ was considered statistically significant differences between the compared groups.

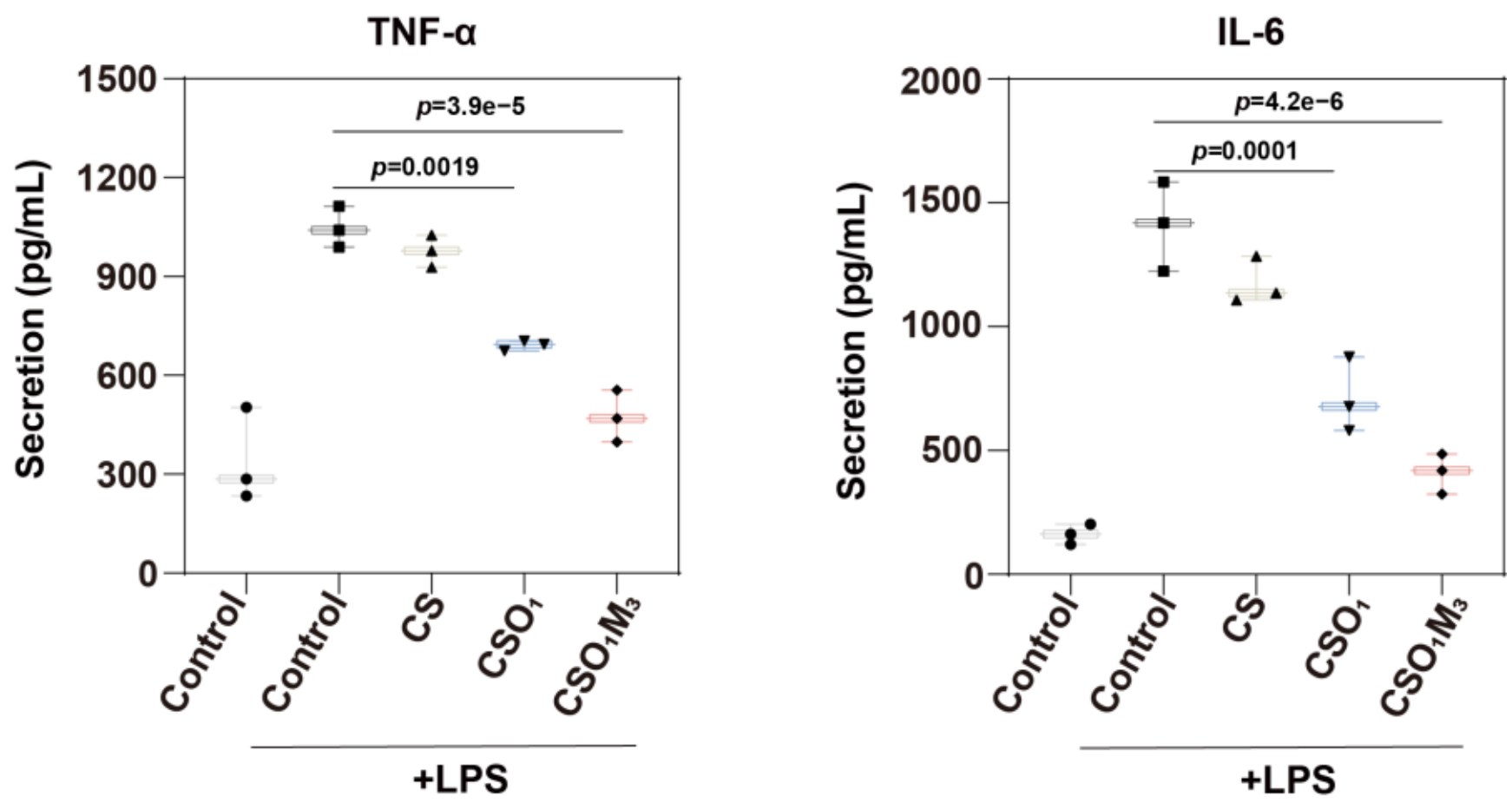

Supplementary Fig. 18. Expression of pro-inflammatory cytokines IL-6 and TNF-α in various groups. The macrophages were co-cultured with different composite membranes under LPS conditions for 48 hours, followed by measuring the secretion of TNF-α and IL-6. Values represent the mean and standard deviation ($n = 3$ independent samples per group). Statistical analyses were performed using one-way ANOVA followed by Tukey's post hoc test. $p < 0.05$ was considered statistically significant differences between the compared groups.

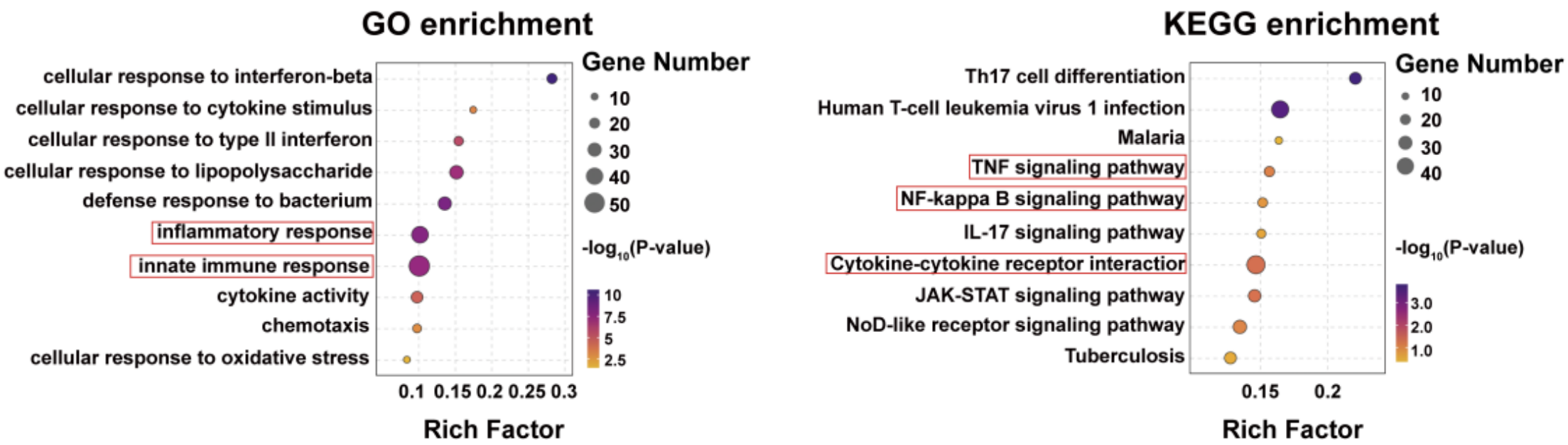

Supplementary Fig. 19. Downregulated of Gene Ontology (GO) terms and Kyoto Encyclopedia of Genes and Genomes (KEGG) pathways in LPS-stimulated macrophages co-cultured with the CSO_1M_3 membranes compared to LPS control group.

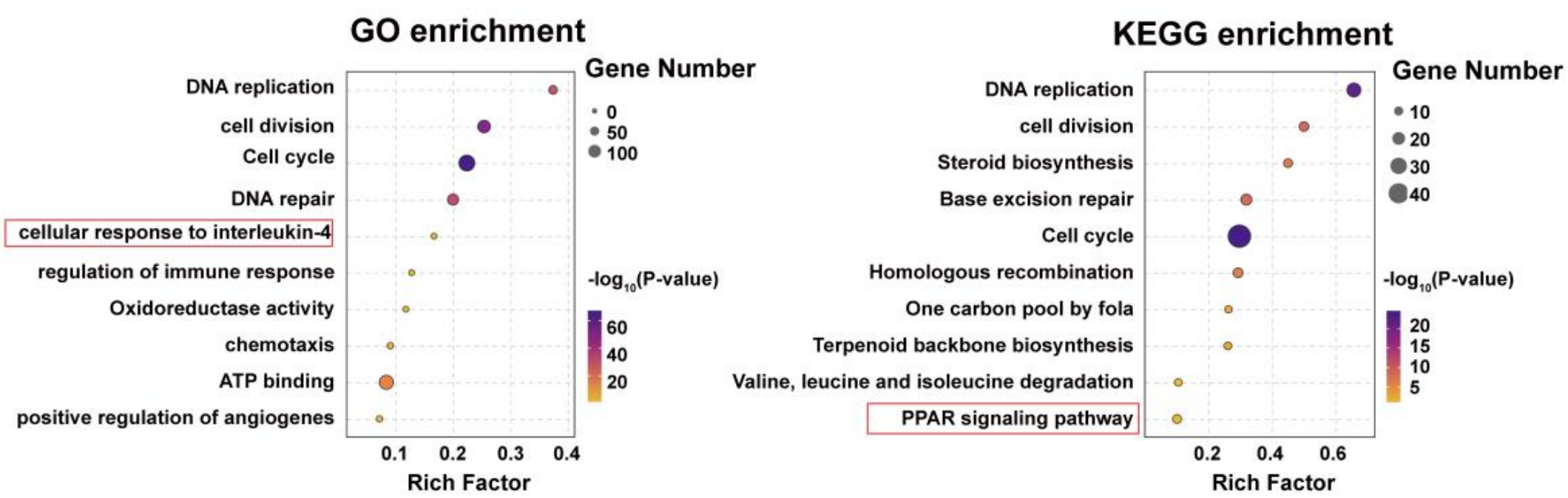

Supplementary Fig. 20. Upregulated GO terms and KEGG pathways in LPS-stimulated macrophages co-cultured with CSO_1M_3 compared to LPS-only controls.

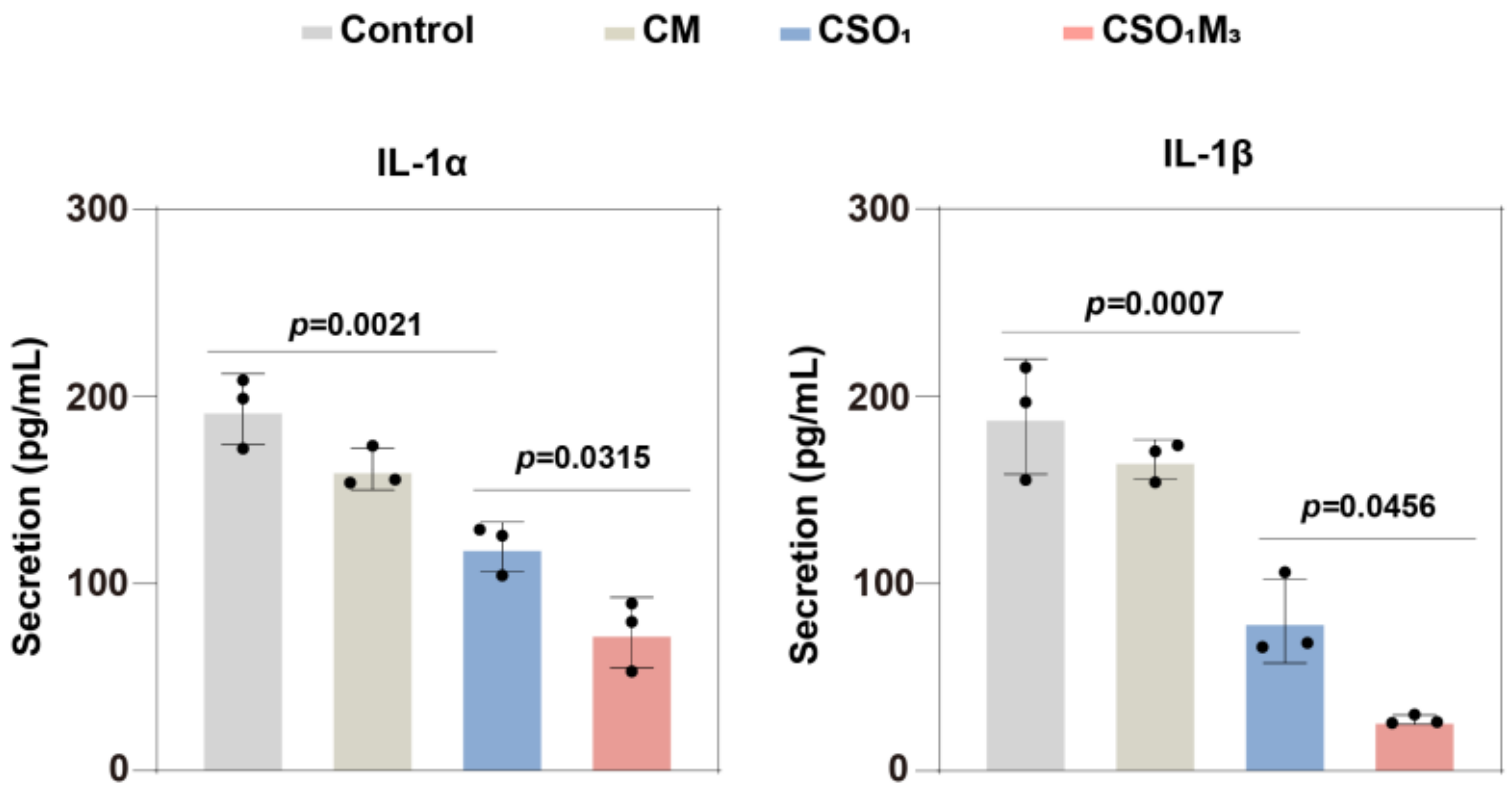

Supplementary Fig. 21. The levels of IL-1α and IL-1β cytokines in the serum of the *rat* model measured by the Luminex assay after implantation for 1 week. Values represent the mean and standard deviation ($n = 3$ independent samples per group). Statistical analyses were performed using one-way ANOVA followed by Tukey's post hoc test. $p < 0.05$ was considered statistically significant differences between the compared groups.

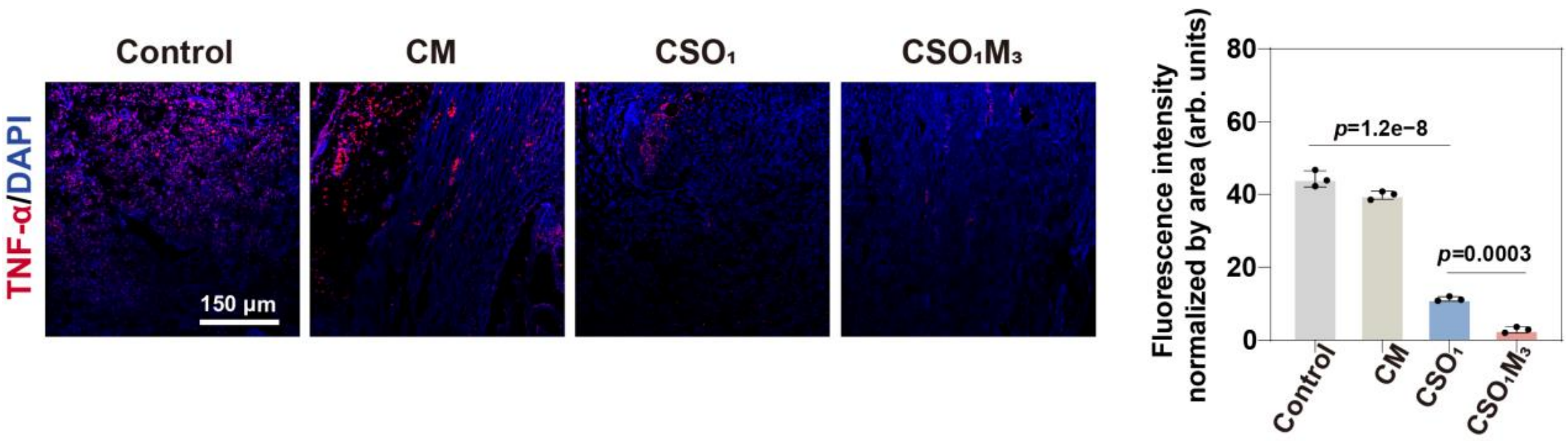

Supplementary Fig. 22. IF staining of TNF-α (red) images of tissues from the defective areas and corresponding quantitative analysis after implantation for 1 week. Values represent the mean and standard deviation ($n = 3$ independent samples per group). Statistical analyses were performed using one-way ANOVA followed by Tukey's post hoc test. $p < 0.05$ was considered statistically significant differences between the compared groups.

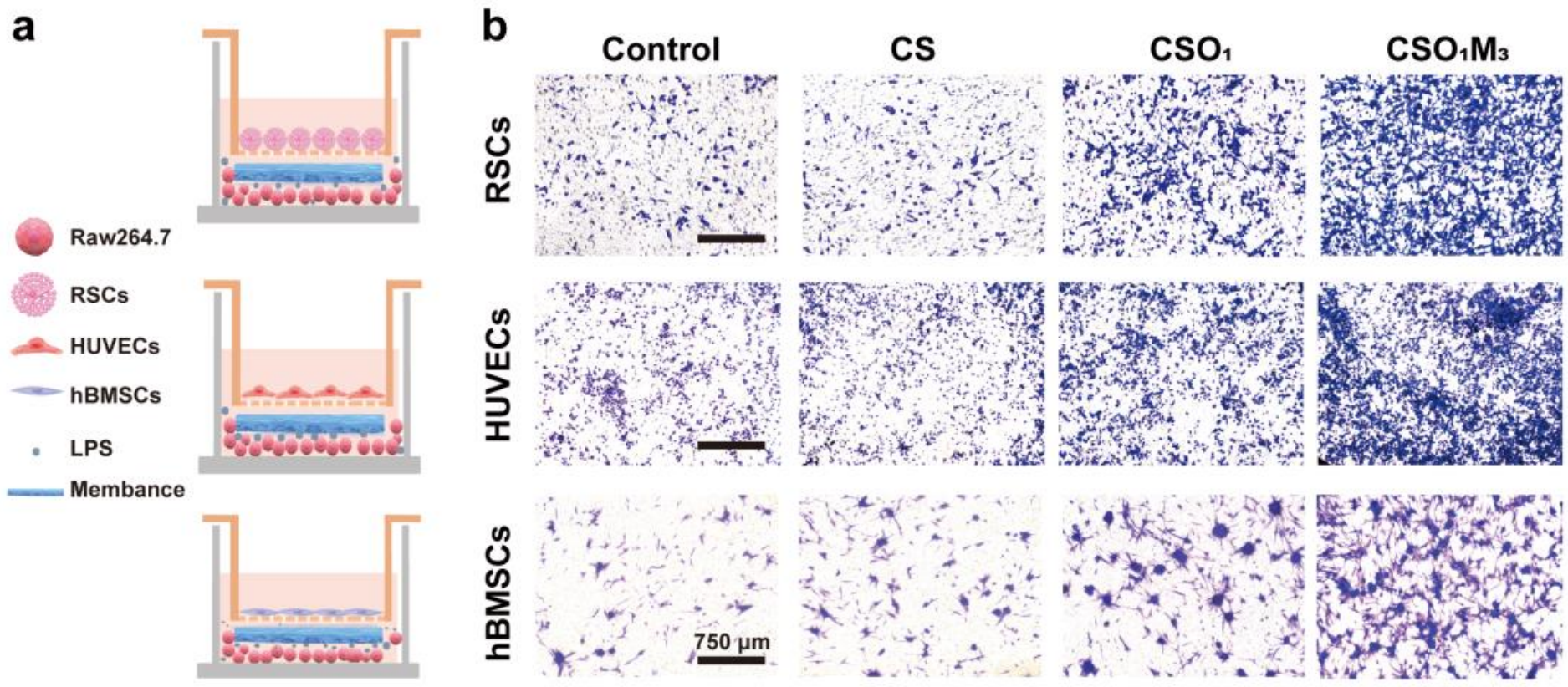

Supplementary Fig. 23. Macrophages-mediated cells recruitment experiments. **a** Schematic illustration of the Transwell co-culture assay. **b** Representative crystal violet-stained images to indicate the migrated RSCs, HUVECs, and hBMSCs using different membranes treated conditional medium in the Transwell assay.

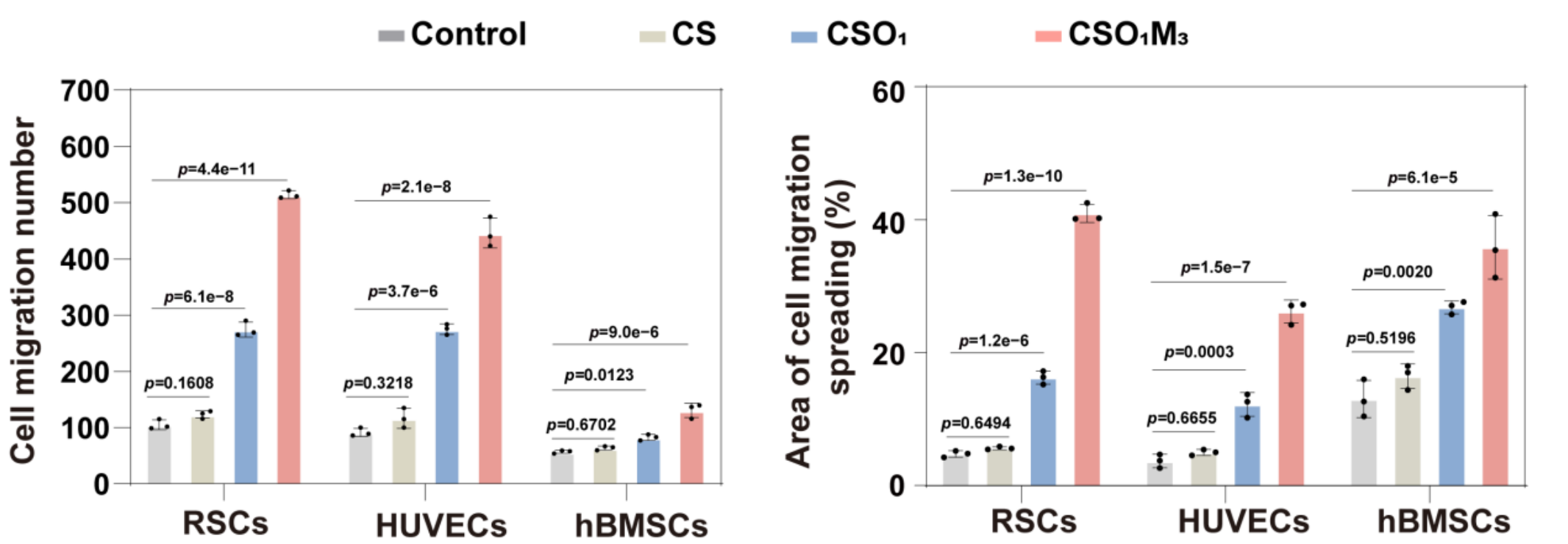

Supplementary Fig. 24. Quantitative analysis of the cells recruitment through the Transwell assay. Migrated cells number and spreading areas were measured based on crystal violet-stained images in Supplementary Fig. 23b. Values represent the mean and standard deviation ($n = 3$ samples per group). Statistical analyses were performed using one-way ANOVA followed by Tukey's post hoc test. $p < 0.05$ was considered statistically significant differences between the compared groups.

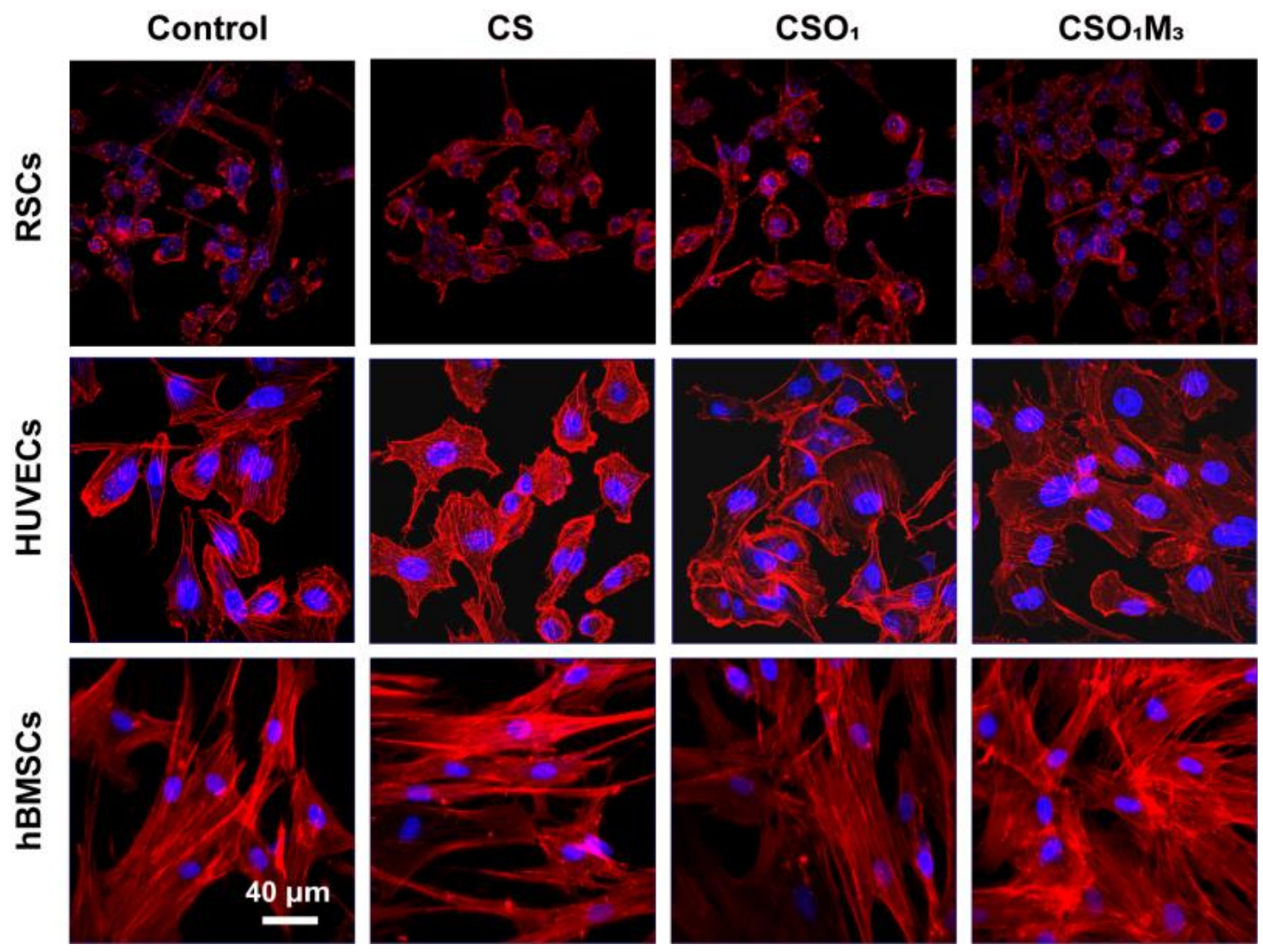

Supplementary Fig. 25. Cytoskeleton staining images of RSCs, HUVECs, and hBMSCs cultured on the blank dishes (control group) and the porous layers of various membranes (CS, CSO_1, and CSO_1M_3 groups). All membranes treated groups exhibited well-spread morphologies, indicating favorable adhesion and extension on the porous microstructures of the membranes.

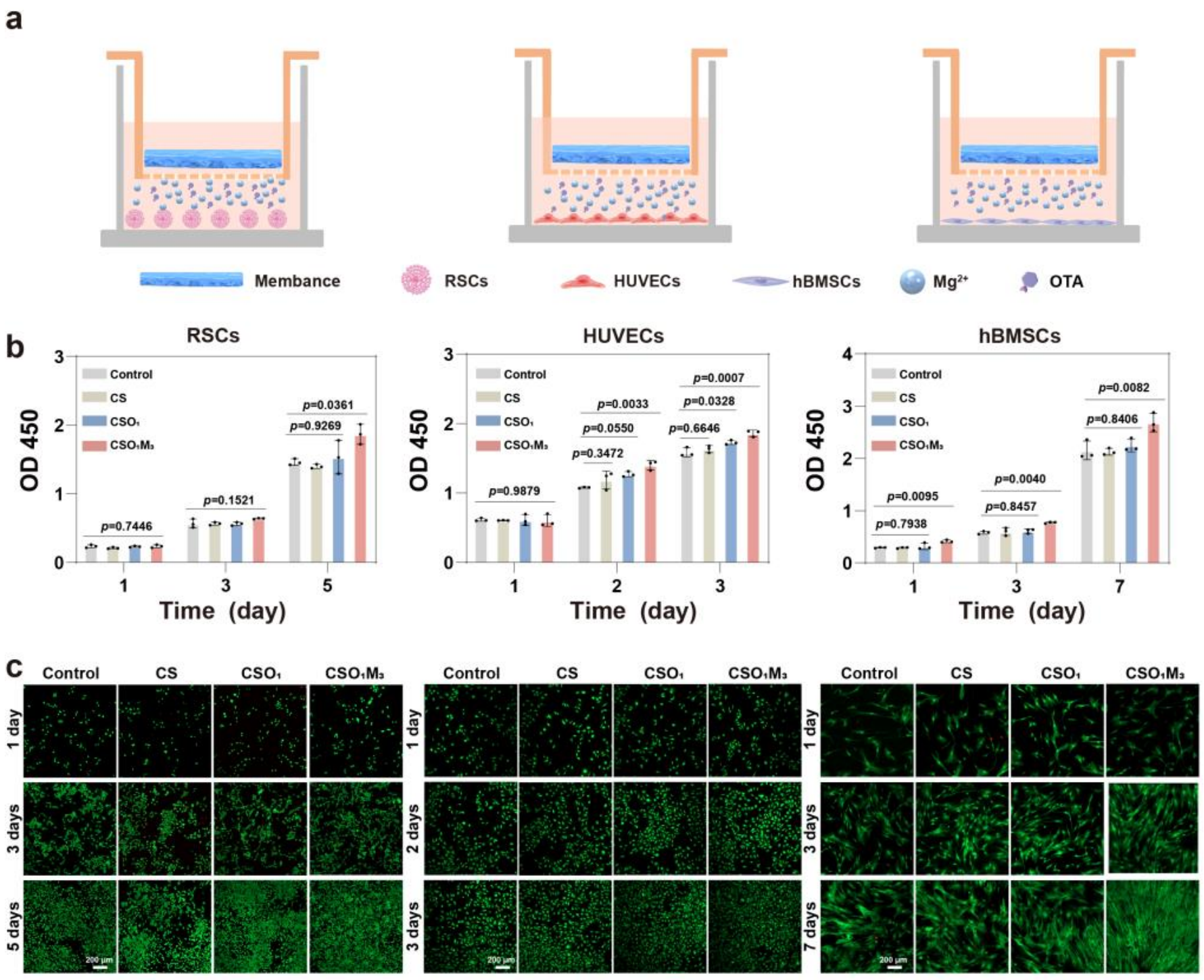

Supplementary Fig. 26. Cell viability and proliferation of RSCs, HUVECs, and hBMSCs co-cultured with different composite membranes at the predetermined time. a Schematic illustration of the co-culture of different cell types with the composite membrane. **b** CCK-8 assay results. **c** Live/dead staining images. Control group: without any membranes. Values represent the mean and standard deviation ($n = 3$ independent samples per group). Statistical analyses were performed using one-way ANOVA followed by Tukey's post hoc test. $p < 0.05$ was considered statistically significant differences between the compared groups.

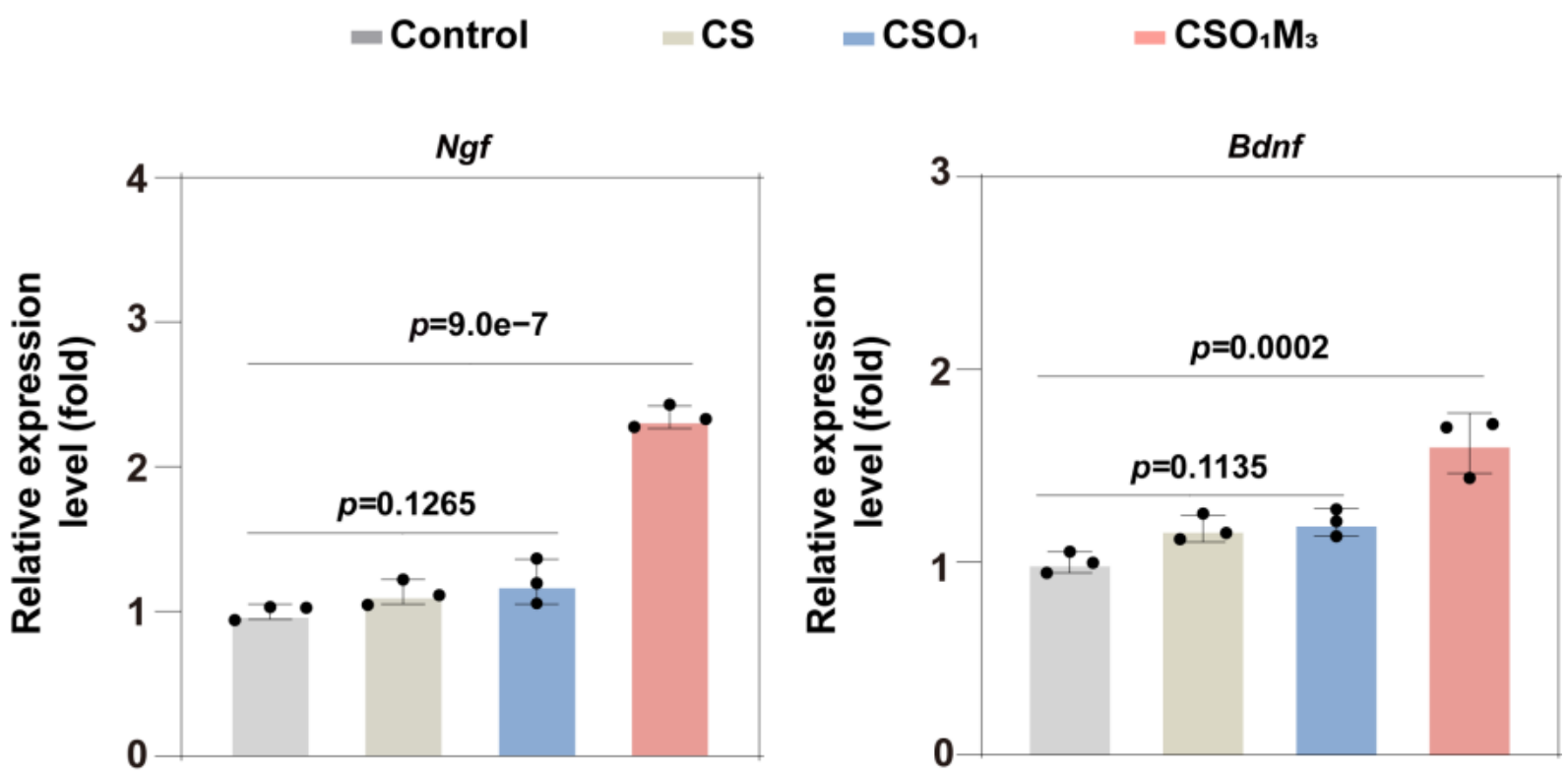

Supplementary Fig. 27. Relative mRNA expression levels of nerve growth factor (*Ngf*) and brain-derived neurotrophic factor (*Bdnf*) of the RSCs after 3 days of co-culture with the composite membranes. Values represent the mean and standard deviation ($n = 3$ independent samples per group). Statistical analyses were performed using one-way ANOVA followed by Tukey's post hoc test. $p < 0.05$ was considered statistically significant differences between the compared groups.

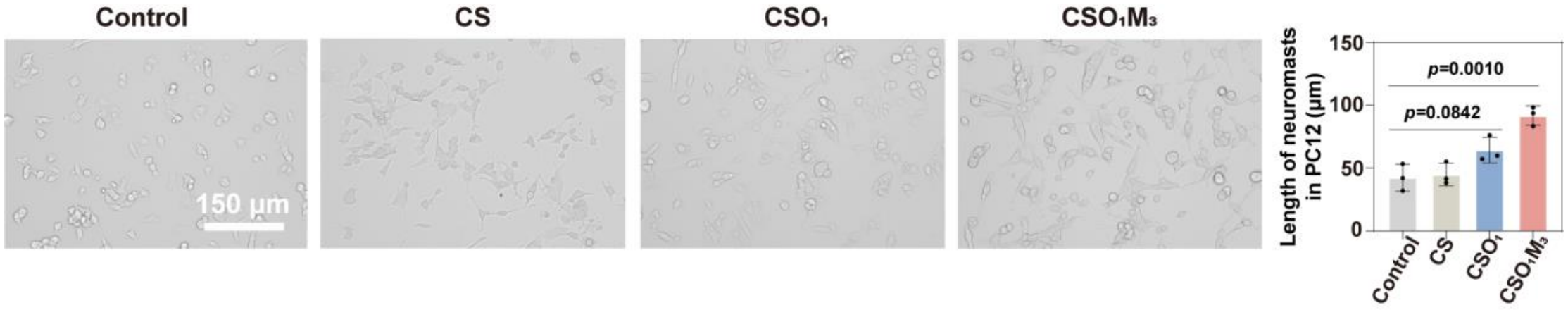

Supplementary Fig. 28. Representative images and corresponding quantitative analysis of the PC12 cells after 1 day of co-culture with various composite membranes. Values represent the mean and standard deviation ($n = 3$ independent samples per group). Statistical analyses were performed using one-way ANOVA followed by Tukey's post hoc test. $p < 0.05$ was considered statistically significant differences between the compared groups.

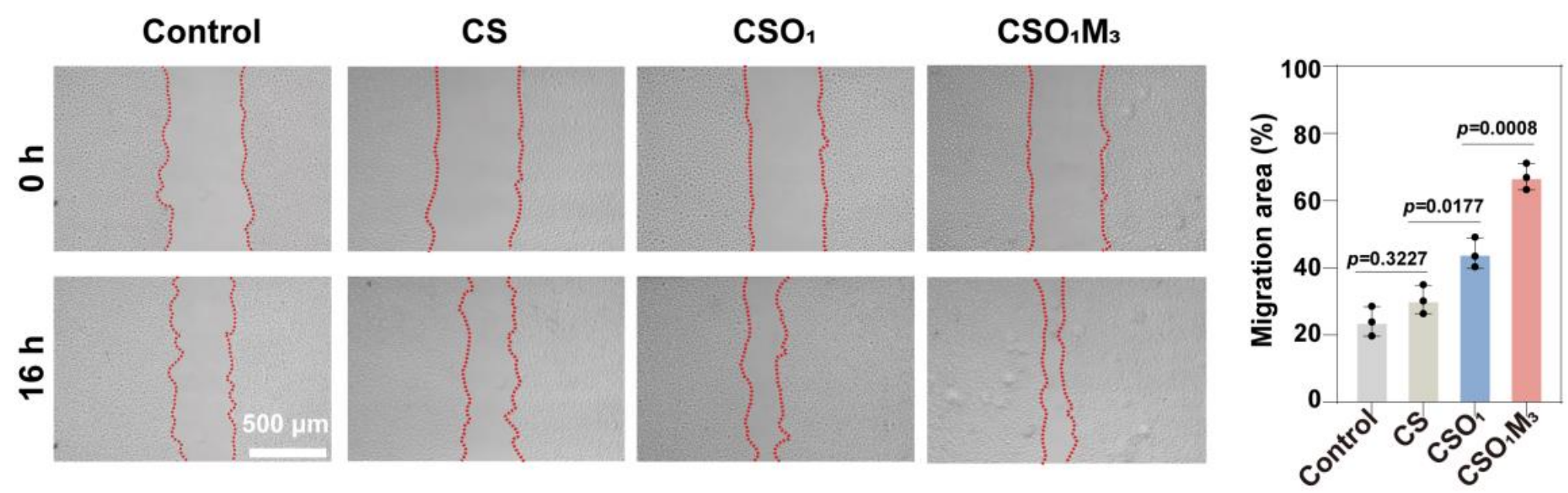

Supplementary Fig. 29. Scratch assay images at 0 h and 16 h and corresponding migration area ratio at 16 h of the HUVECs treated by the extracts from different composite membranes under serum-free conditions. The migration area ratio was calculated as the percentage of the wound area closed relative to the initial scratch area. Values represent the mean and standard deviation ($n = 3$ independent samples per group). Statistical analyses were performed using one-way ANOVA followed by Tukey's post hoc test. $p < 0.05$ was considered statistically significant differences between the compared groups.

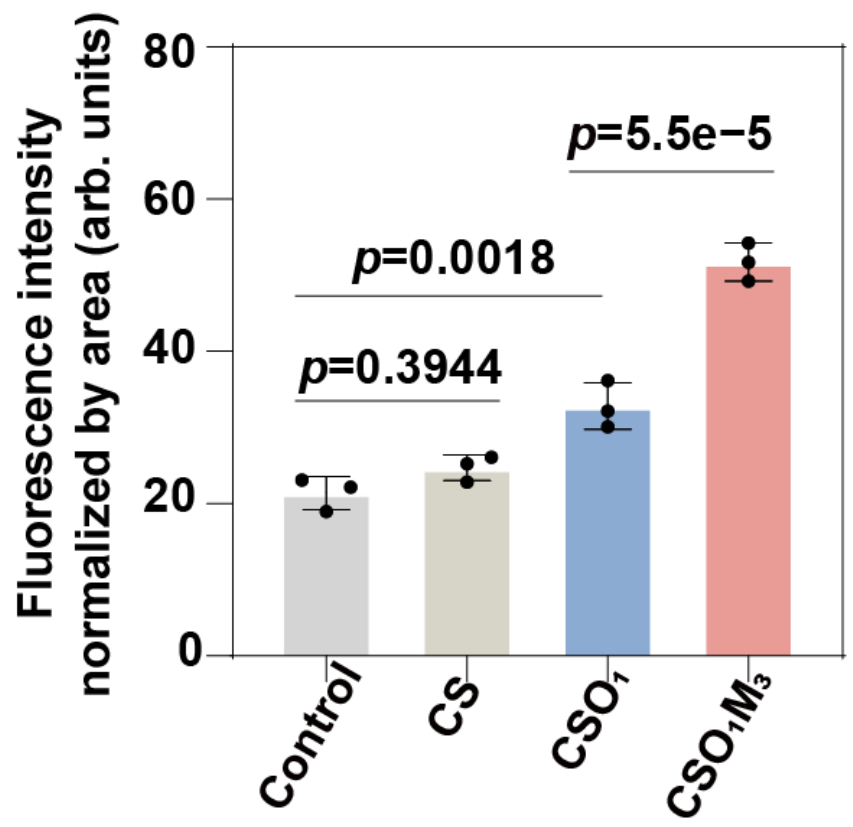

Supplementary Fig. 30. Quantitative analysis of VEGF expression of the HUVECs co-cultured with various composite membranes based on the IF staining images in Fig. 5h. Values represent the mean and standard deviation ($n = 3$ independent samples per group). Statistical analyses were performed using one-way ANOVA followed by Tukey's post hoc test. $p < 0.05$ was considered statistically significant differences between the compared groups.

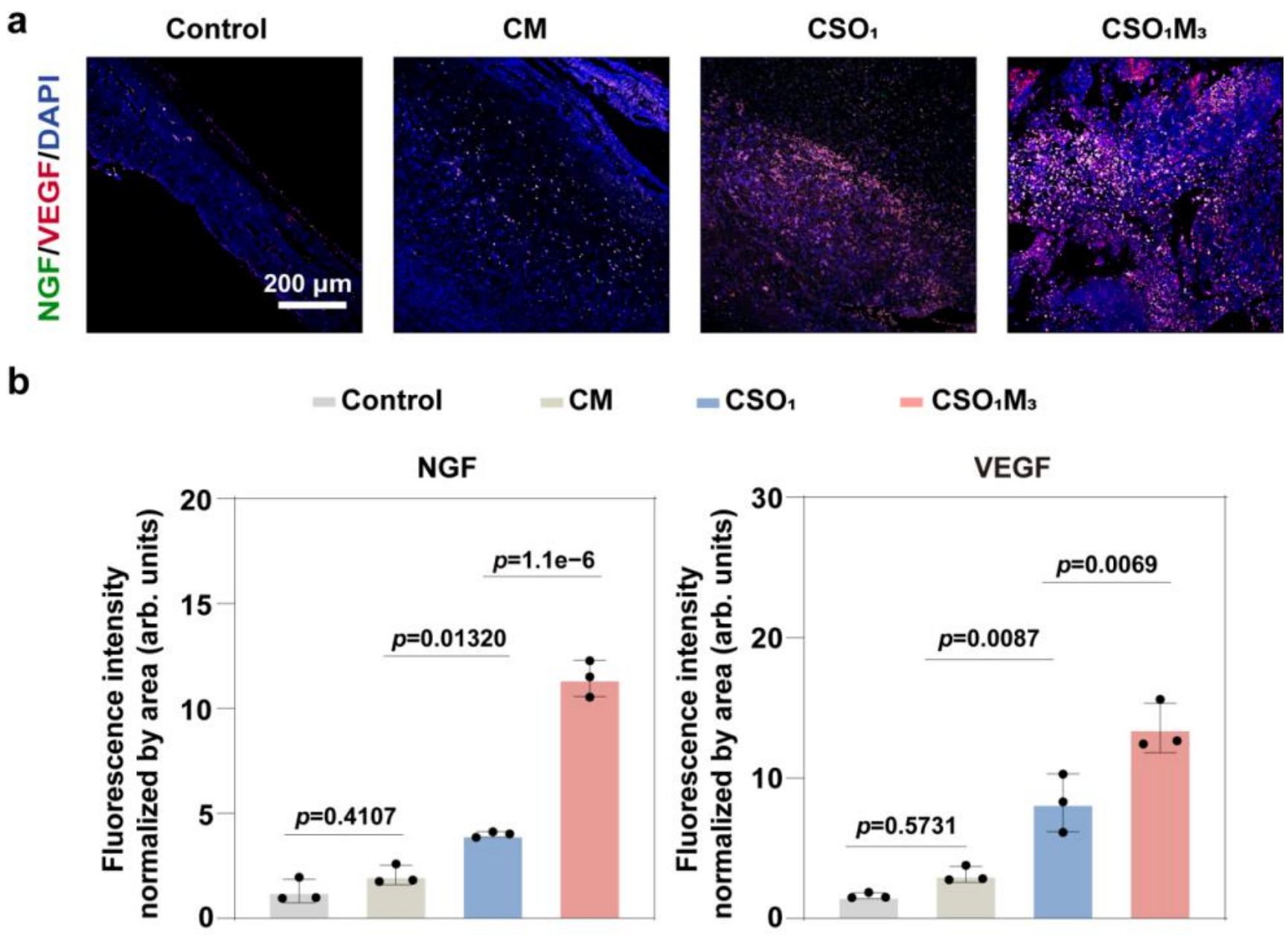

Supplementary Fig. 31. Quantitative and qualitative co-localized expressions of NGF and VEGF at the cranial defective sites after implantation for 1 week. a Representative IF staining images. **b** Quantitative fluorescence intensities based on images in **(a)**. Values in (b) represent the mean and standard deviation ($n = 3$ independent samples per group). Statistical analyses were performed using one-way ANOVA followed by Tukey's post hoc test. $p < 0.05$ was considered statistically significant differences between the compared groups.

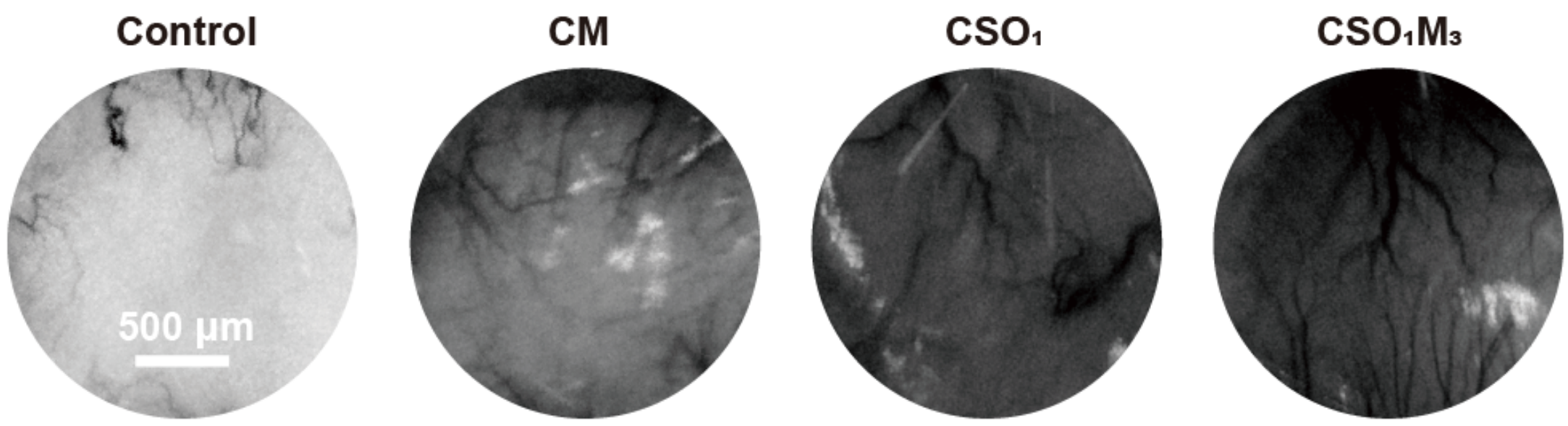

Supplementary Fig. 32. Representative grayscale images through the Laser speckle contrast imaging (LSCI) method at the cranial defective sites after implantation for 2 weeks.

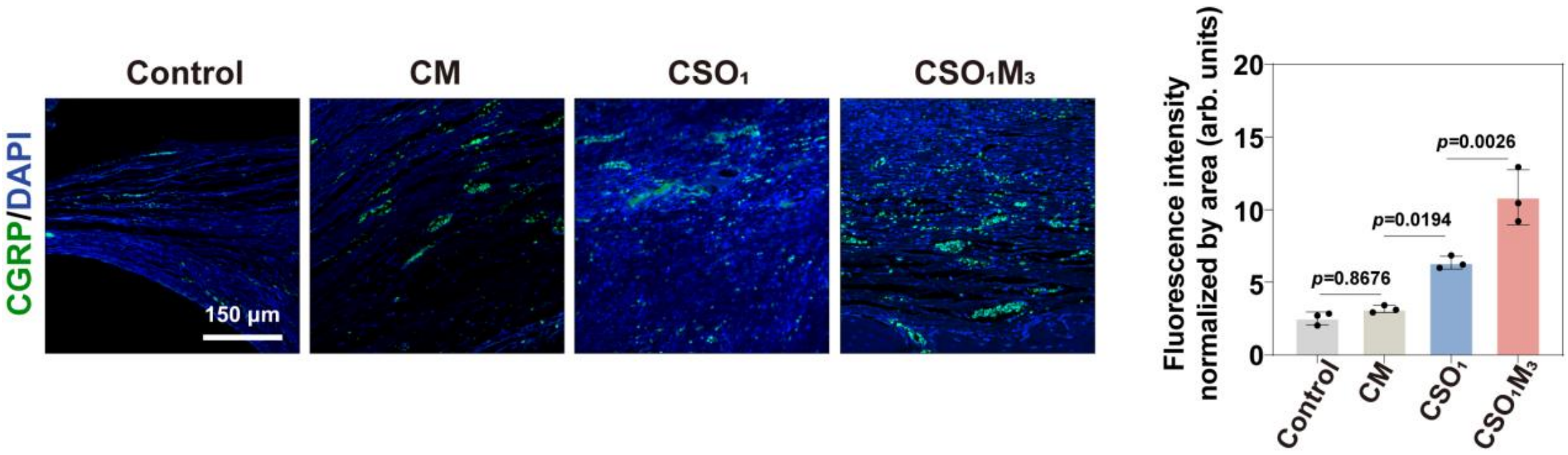

Supplementary Fig. 33. Representative IF staining images and corresponding quantitative analysis of the CGRP at the bone defective sites after implantation for 4 weeks. Values represent the mean and standard deviation ($n = 3$ independent samples per group). Statistical analyses were performed using one-way ANOVA followed by Tukey's post hoc test. $p < 0.05$ was considered statistically significant differences between the compared groups.

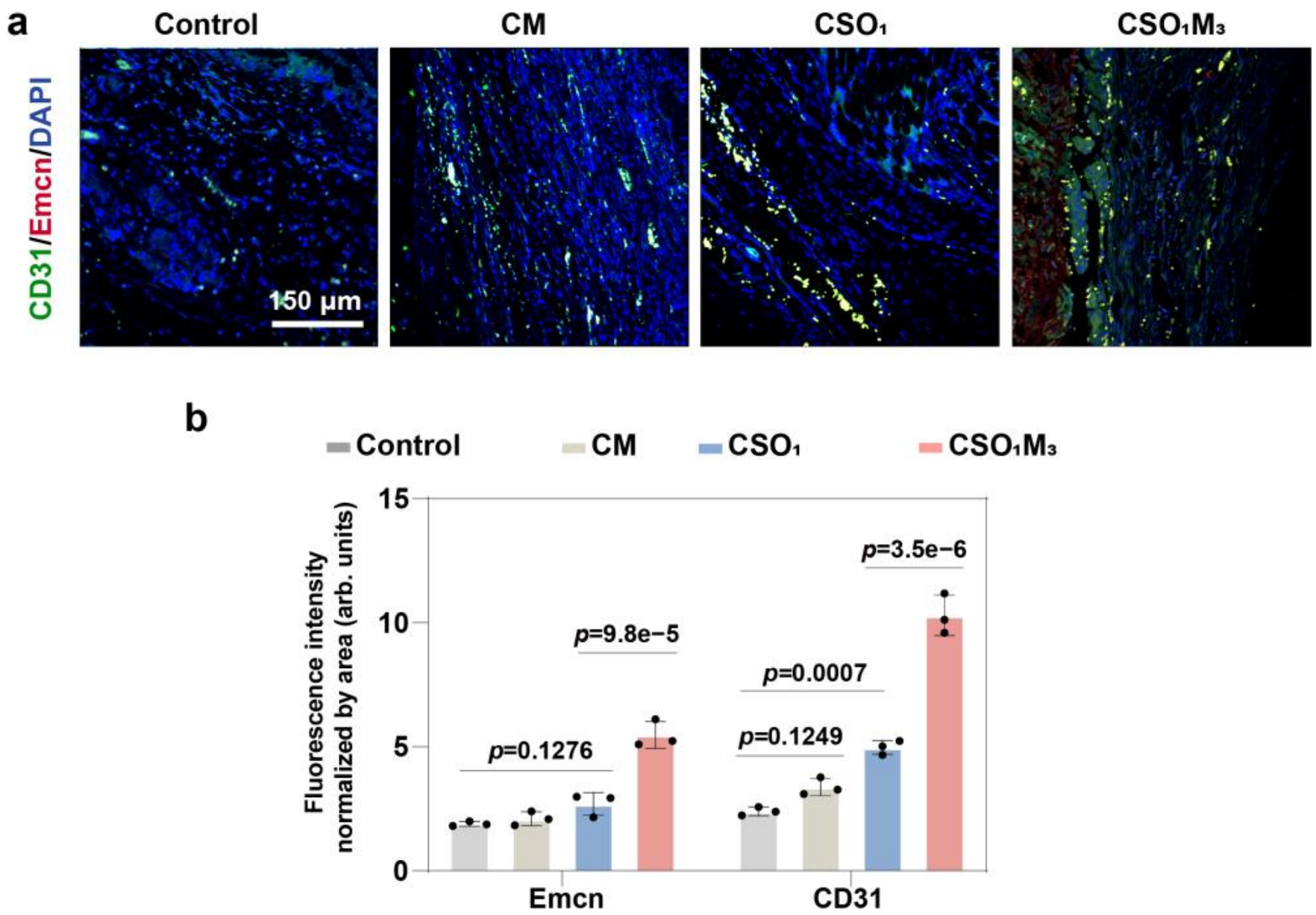

Supplementary Fig. 34. Characterization of H-type vessels at the bone defective sites after implantation for 4 weeks. a Representative IF staining of CD31 (green) and Emcn (red) images of tissues from the defective sites. **b** Quantitative fluorescence intensities based on the co-localized images in **(a)**. Values in (b) represent the mean and standard deviation ($n = 3$ independent samples per group). Statistical analyses were performed using one-way ANOVA followed by Tukey's post hoc test. $p < 0.05$ was considered statistically significant differences between the compared groups.

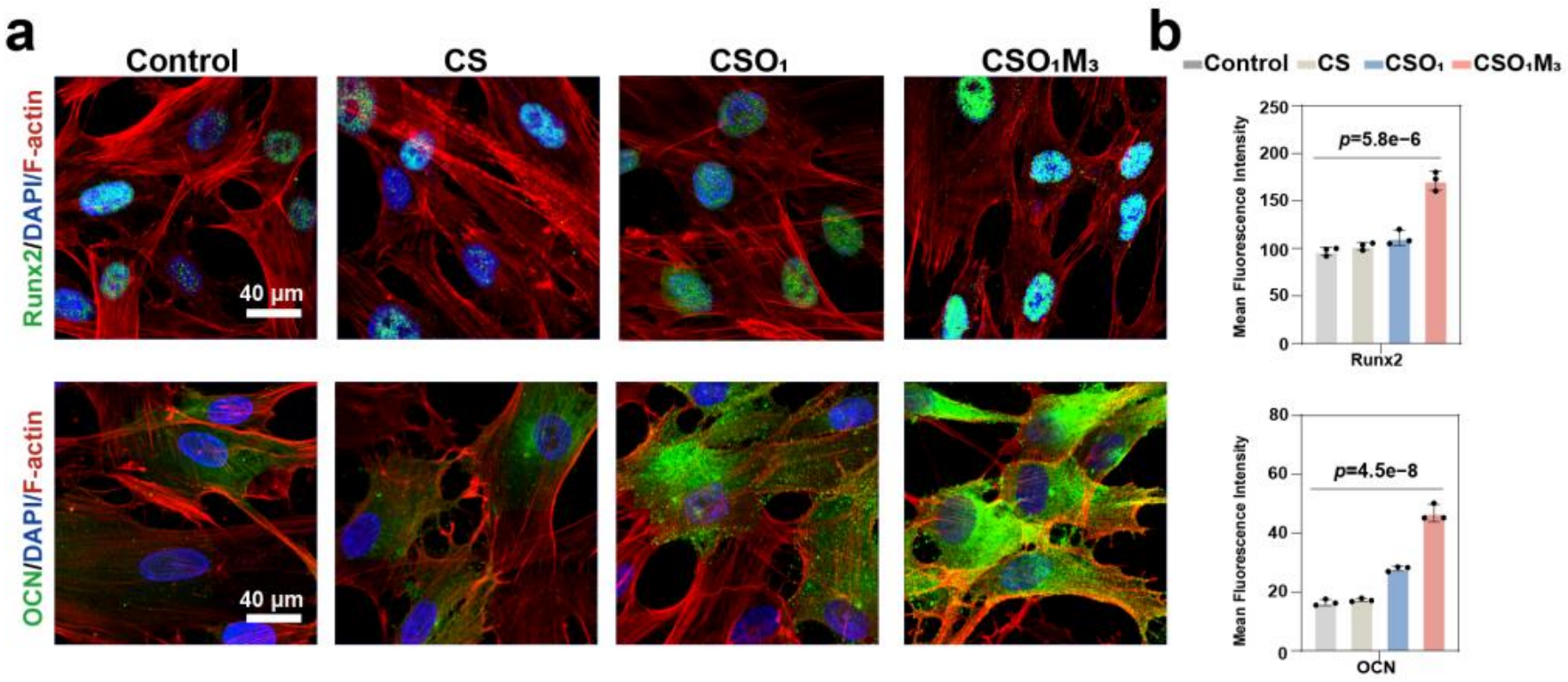

Supplementary Fig. 35. Representative IF staining of Runx2 (a) and OCN (b) images of the hBMSCs co-cultured in different groups for 7 and 14 days, respectively. Values in (b) represent the mean and standard deviation ($n = 3$ independent samples per group). Statistical analyses were performed by one-way ANOVA followed by Tukey's post hoc test. $p < 0.05$ was considered statistically significant differences between the compared groups.

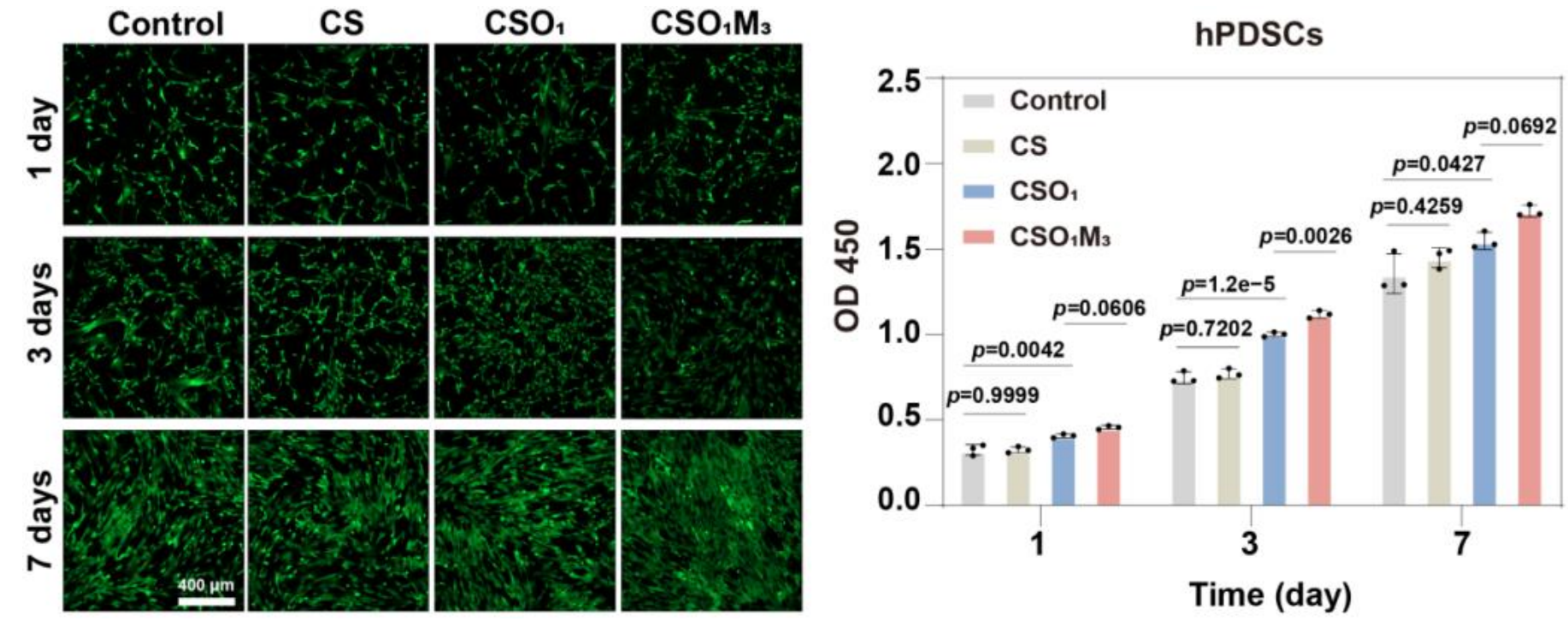

Supplementary Fig. 36. Live/dead staining images and CCK-8 assay results of *human* periosteal-derived stem cells (hPDSCs) co-cultured with different composite membranes at 1, 3, and 7 days. Values represent the mean and standard deviation ($n = 3$ independent samples per group). Statistical analyses were performed using one-way ANOVA followed by Tukey's post hoc test. $p < 0.05$ was considered statistically significant differences between the compared groups.

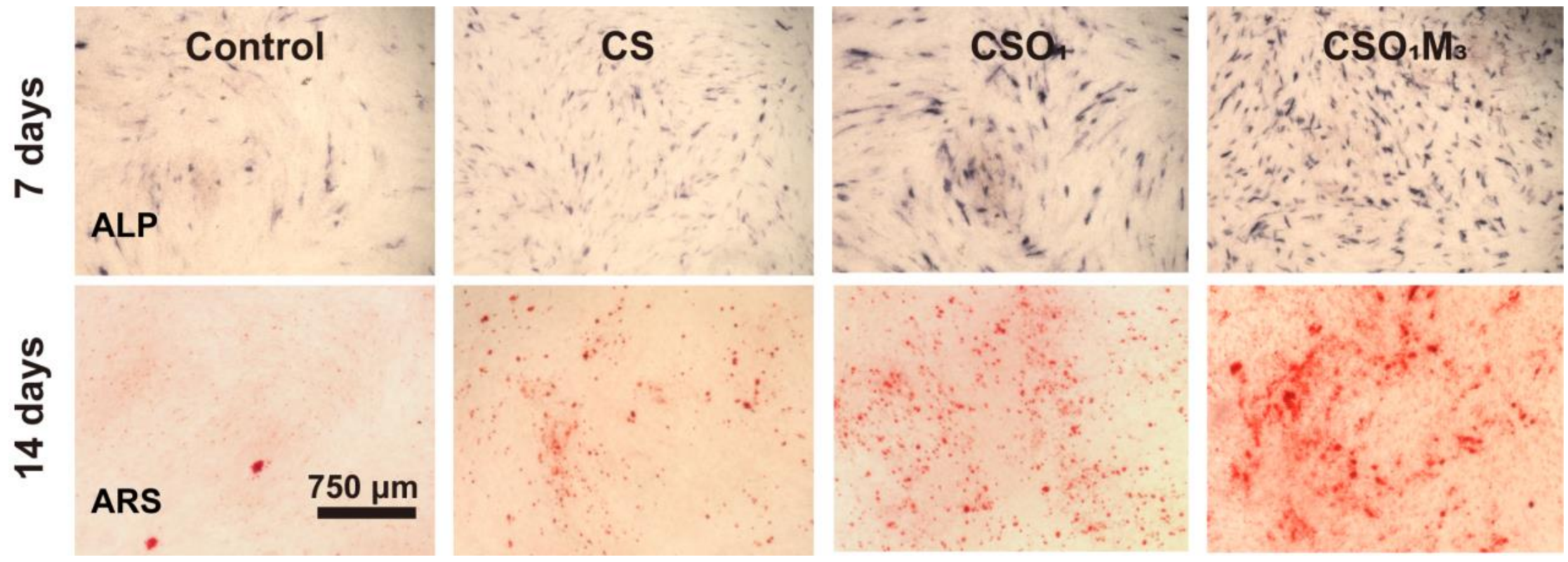

Supplementary Fig. 37. Osteogenic differentiation of hPDSCs co-cultured with different composite membranes through Alkaline phosphatase (ALP) staining at 7 days and Alizarin Red S (ARS) staining at 14 days.

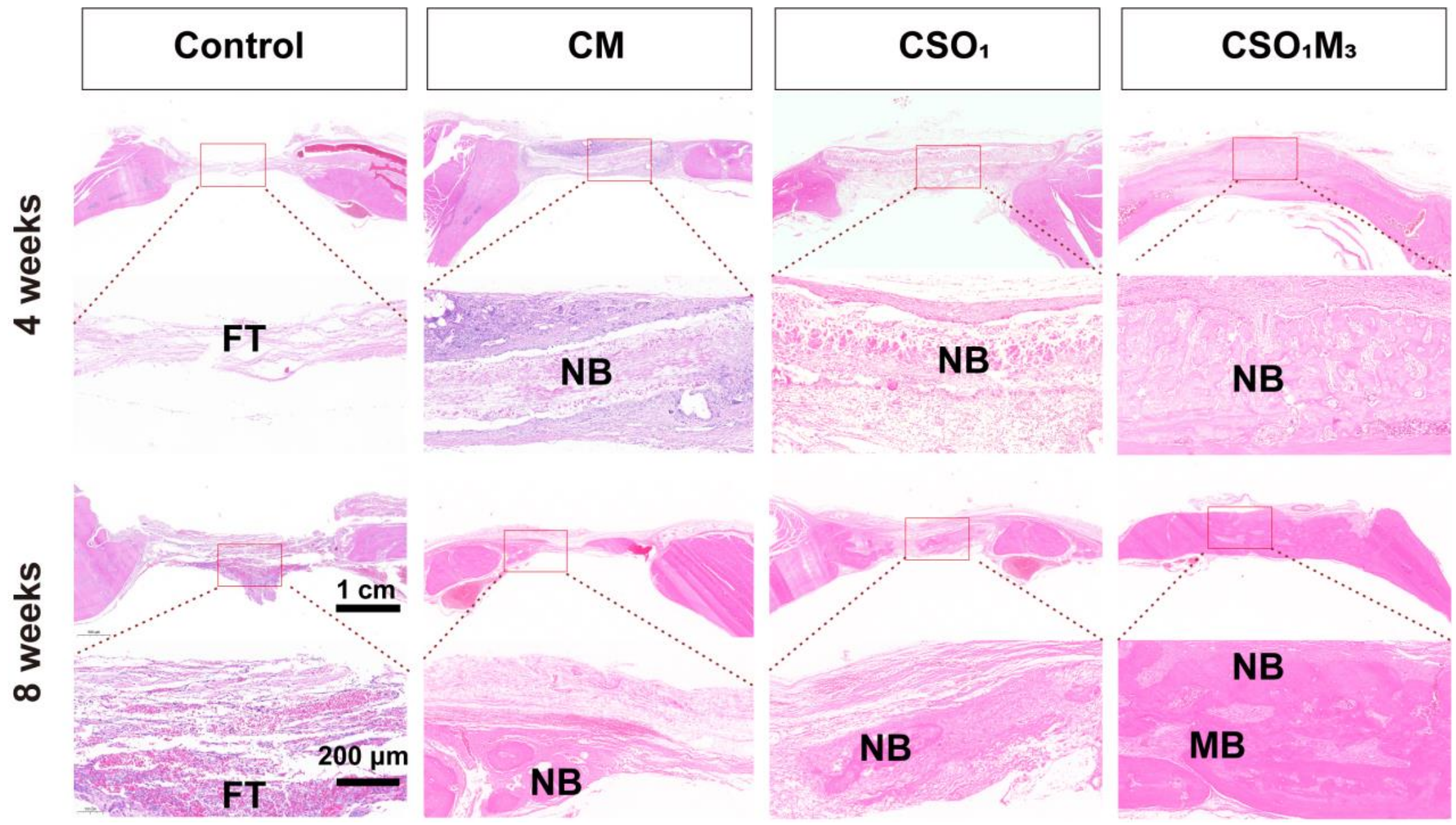

Supplementary Fig. 38. Representative H&E staining images of tissues surrounding bone defects. FT: fibrous tissue. NB: newly formed bone. MB: mature bone. The images were repeated at least three times with consistent results.

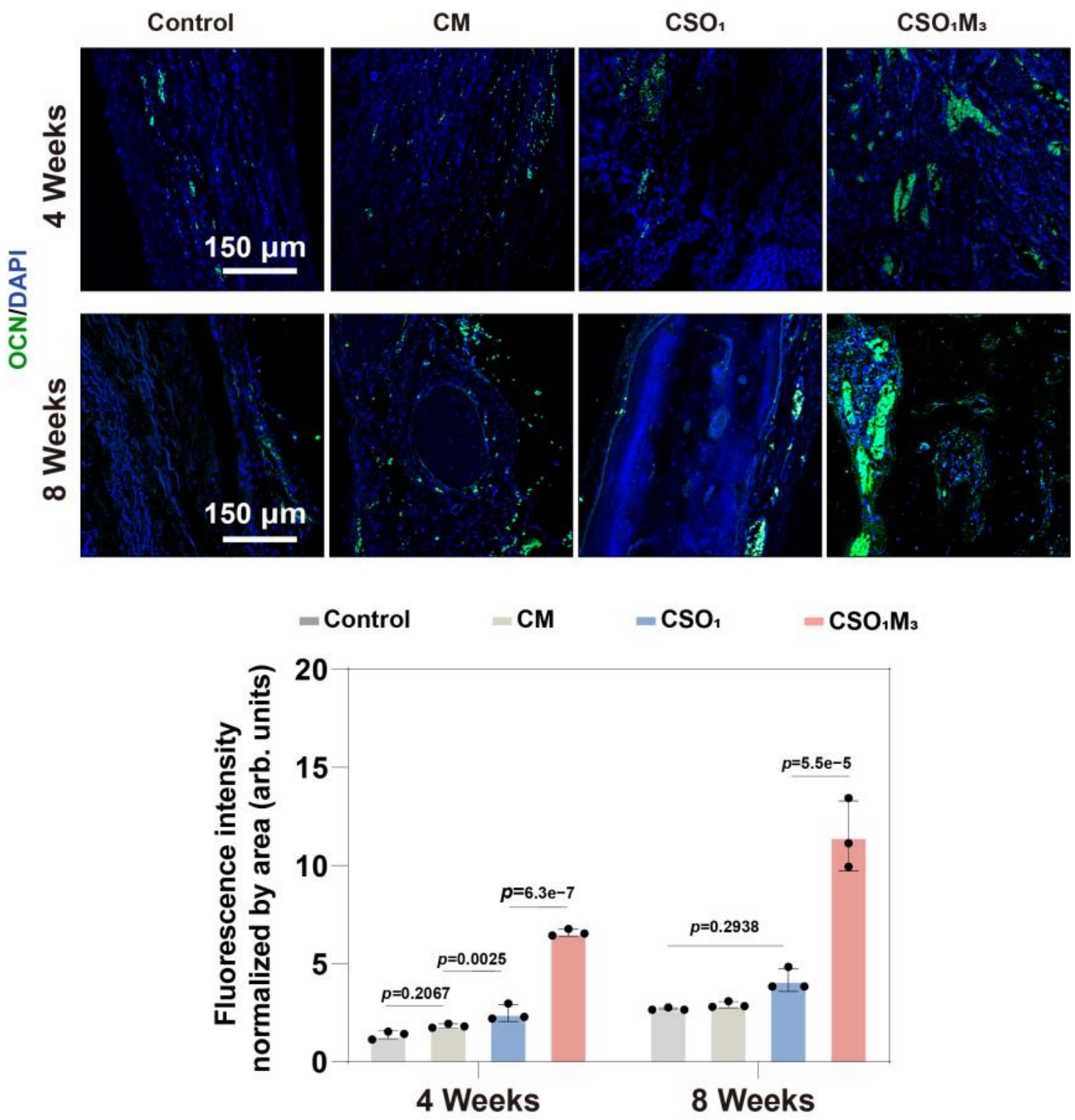

Supplementary Fig. 39. Representative IF staining of osteocalcin (OCN) images of the tissues from defective sites at 4 and 8 weeks post implantation and corresponding quantitative fluorescence intensities. Values represent the mean and standard deviation ($n = 3$ independent samples per group). Statistical analyses were performed using one-way ANOVA followed by Tukey's post hoc test. $p < 0.05$ was considered statistically significant differences between the compared groups.

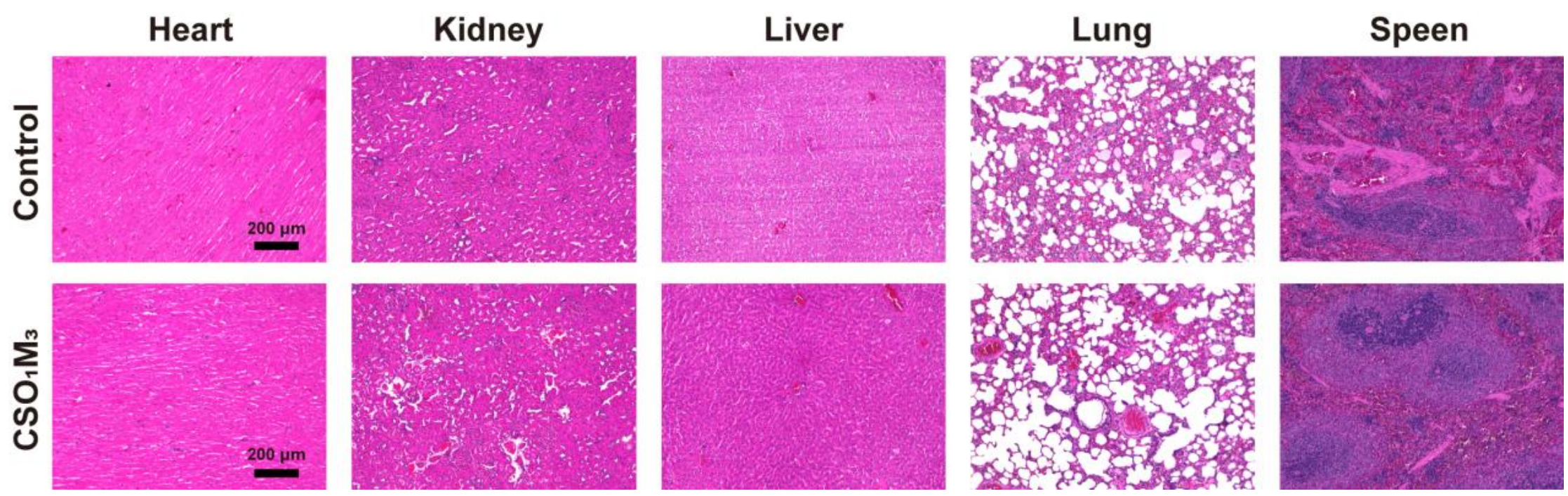

Supplementary Fig. 40. Representative H&E staining images of harvested heart, liver, spleen, lung, and kidney tissues from the blank control group and the CSO_1M_3 group at 8 weeks post-implantation. The images were repeated at least three times with consistent results.

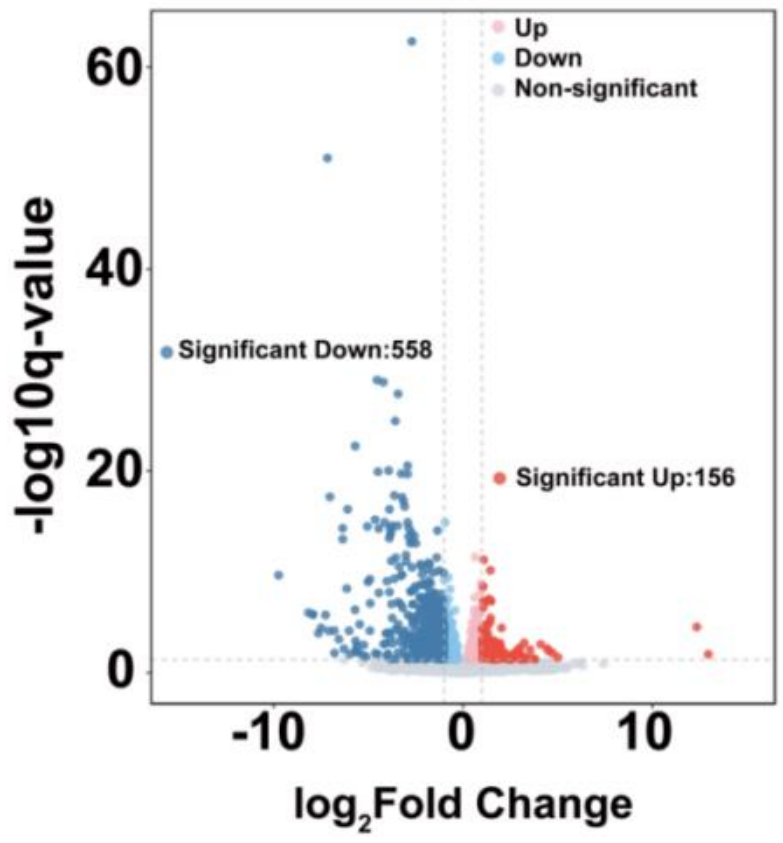

Supplementary Fig. 41. Volcano plot of differentially expressed genes obtained from tissues at bone defective sites with or without the CSO_1M_3 membranes implantation for 4 weeks.

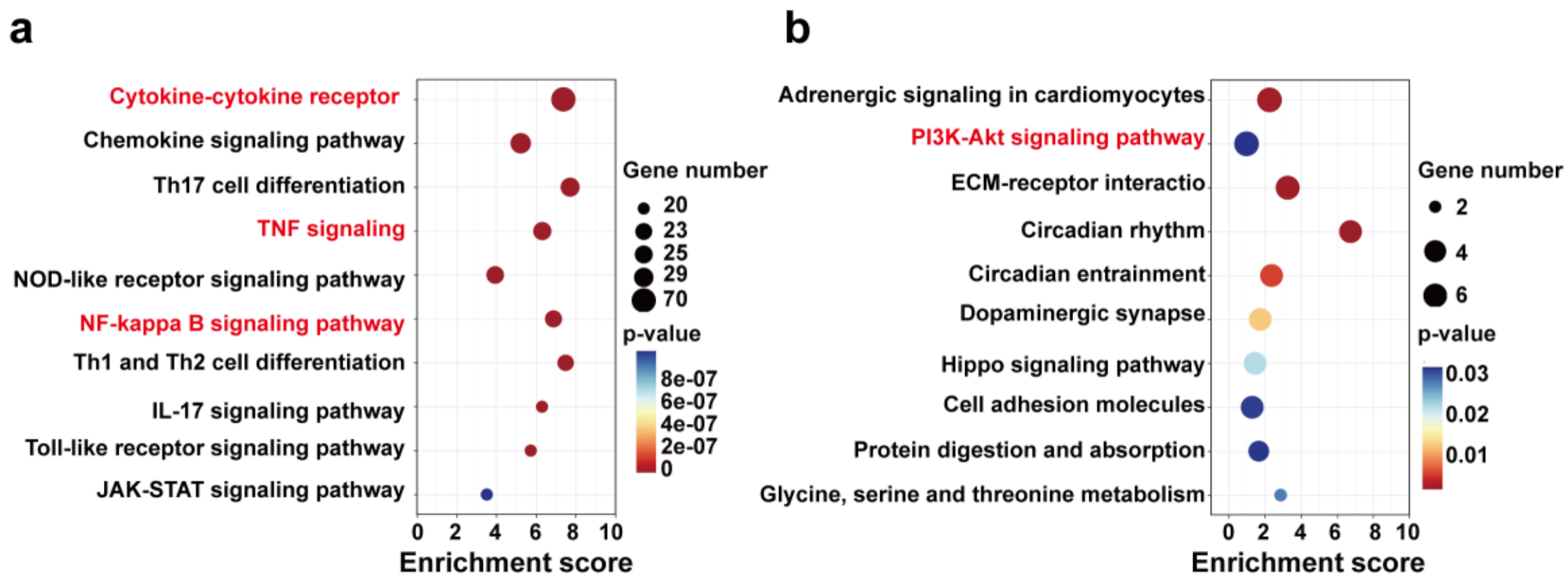

Supplementary Fig. 42. KEGG pathway enrichment analysis of differentially expressed genes in the CSO_1M_3 group at 4 weeks post implantation. a Downregulated pathways. **b** Upregulated pathways.

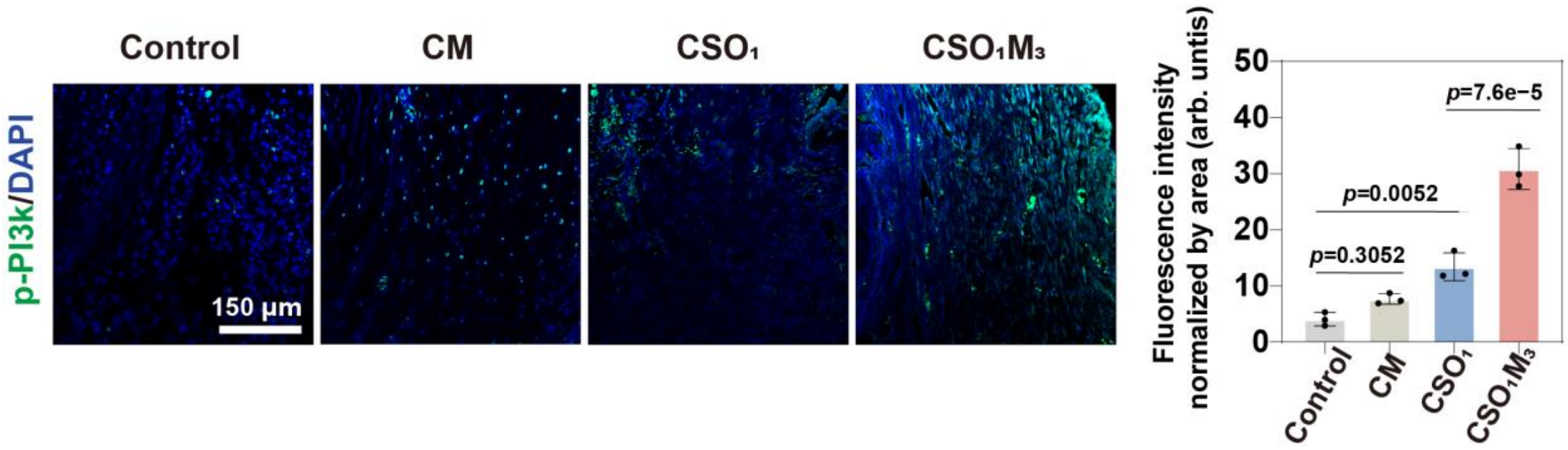

Supplementary Fig. 43. Representative IF staining of phosphorylated PI3K (p-PI3K) images of the tissues from the defective sites at 4 weeks post implantation and corresponding quantitative fluorescence intensities. Values represent the mean and standard deviation ($n = 3$ independent samples per group). Statistical analyses were performed using one-way ANOVA followed by Tukey's post hoc test. $p < 0.05$ was considered statistically significant differences between the compared groups.

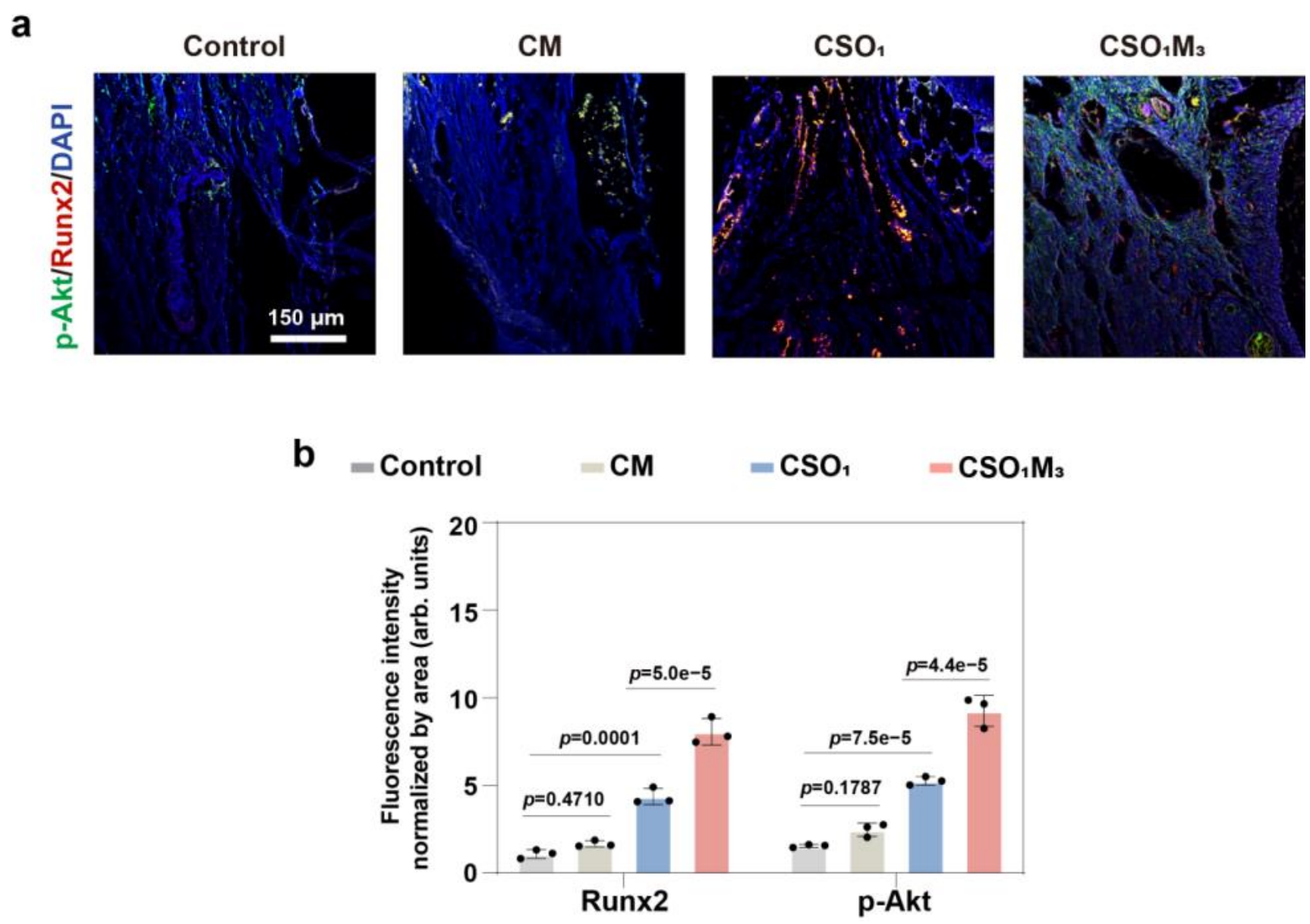

Supplementary Fig. 44. Representative IF staining of phosphorylated Akt (p-Akt) (green) and Runx2 (red) images (a) of the tissues from the defective sites at 4 weeks post implantation and corresponding quantitative fluorescence intensities (b). Values in (b) represent the mean and standard deviation ($n = 3$ independent samples per group). Statistical analyses were performed using one-way ANOVA followed by Tukey's post hoc test. $p < 0.05$ was considered statistically significant differences between the compared groups.

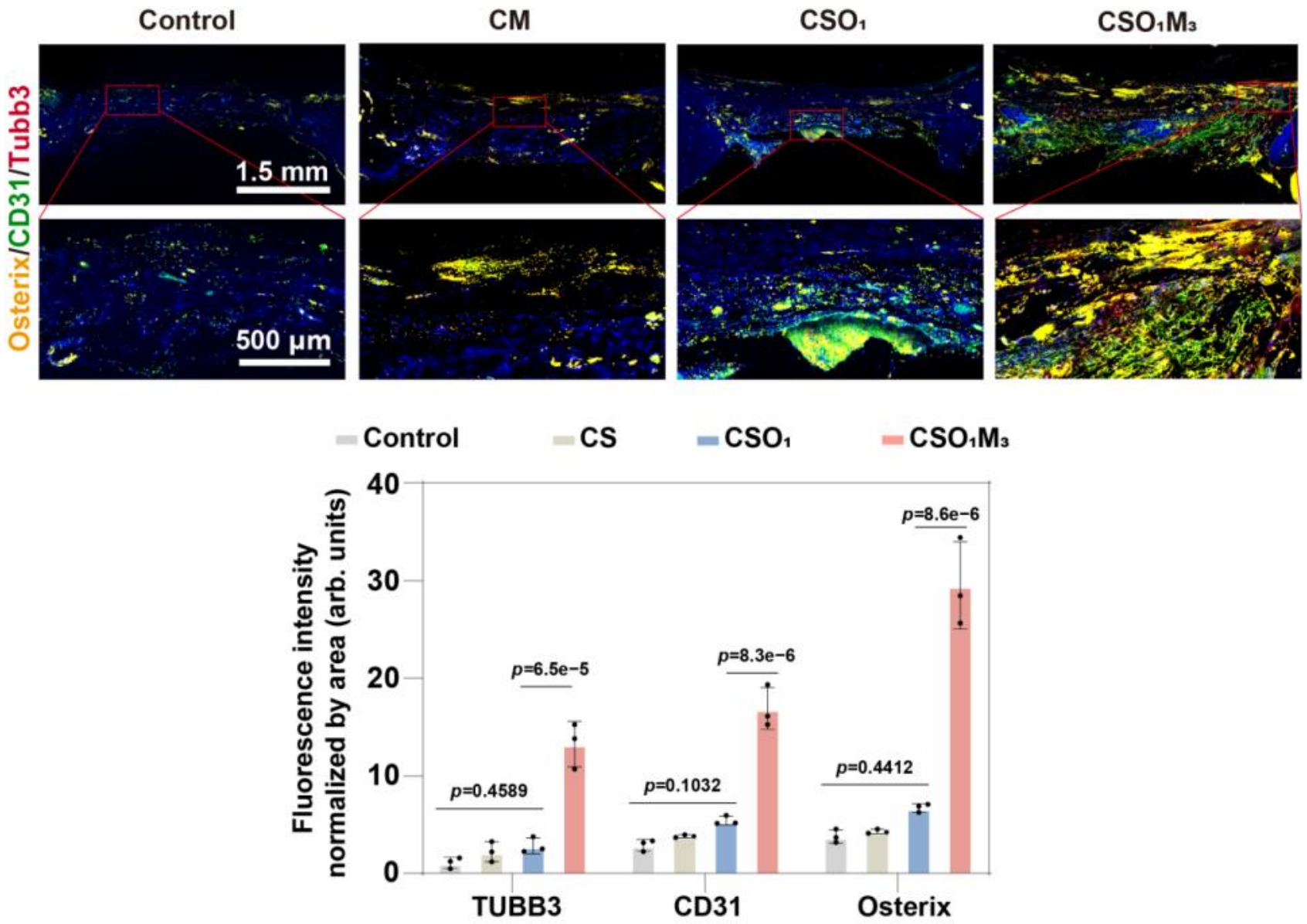

Supplementary Fig. 45. Representative IF staining of TUBB3 (red, neural), CD31 (green, vascular) and Osterix (orange, osteogenic) images of the tissues from defective sites at 4 weeks post implantation and corresponding quantitative fluorescence intensities. Values represent the mean and standard deviation ($n = 3$ independent samples per group). Statistical analyses were performed using one-way ANOVA followed by Tukey's post hoc test. $p < 0.05$ was considered statistically significant differences between the compared groups.

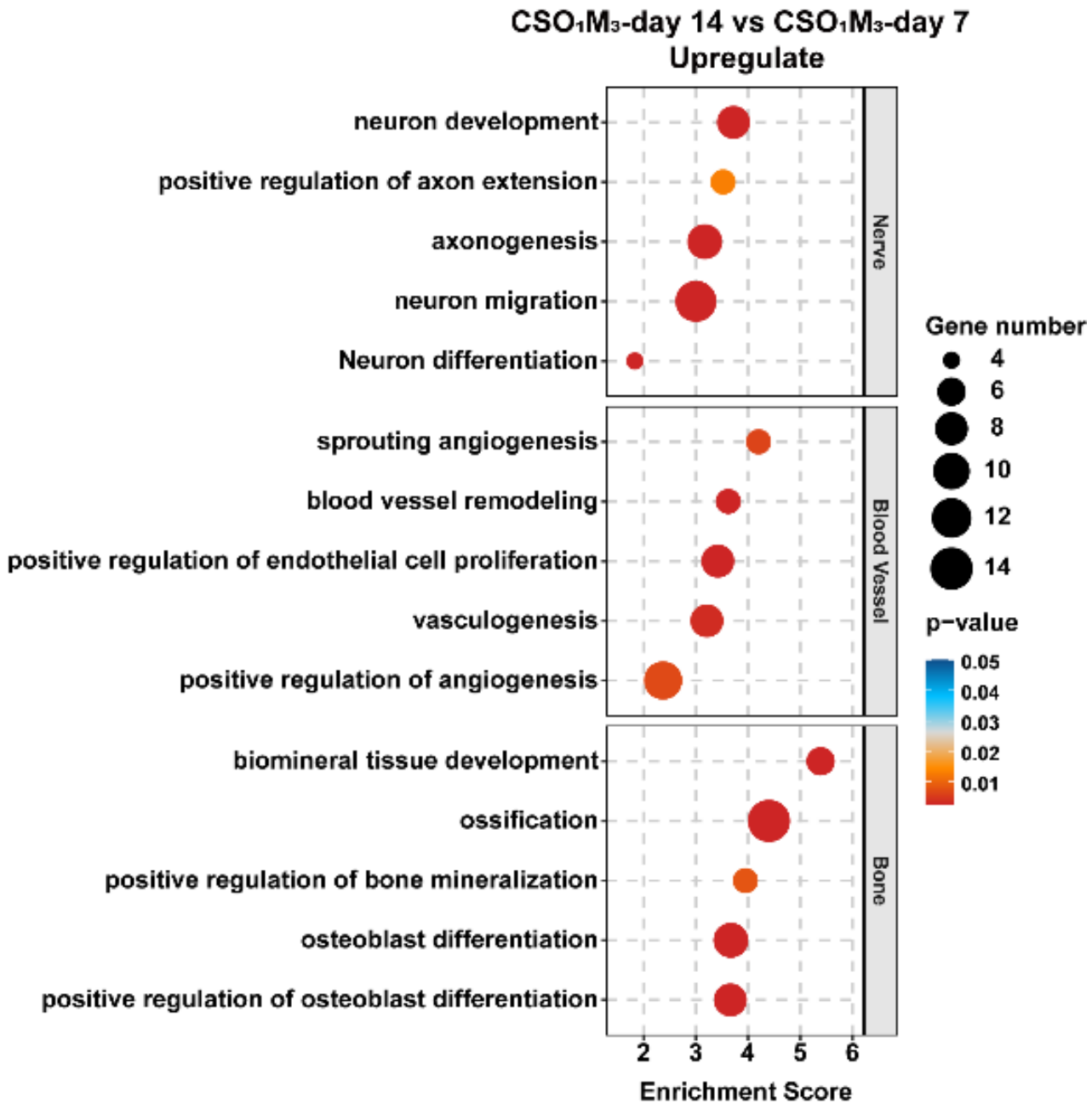

Supplementary Fig. 46. Single-cell transcriptomic comparison between the CSO_1M_3 groups at 1 week and 2 weeks post implantation. GO enrichment analysis of upregulated genes related to nerve, blood vessel and bone tissues regeneration.

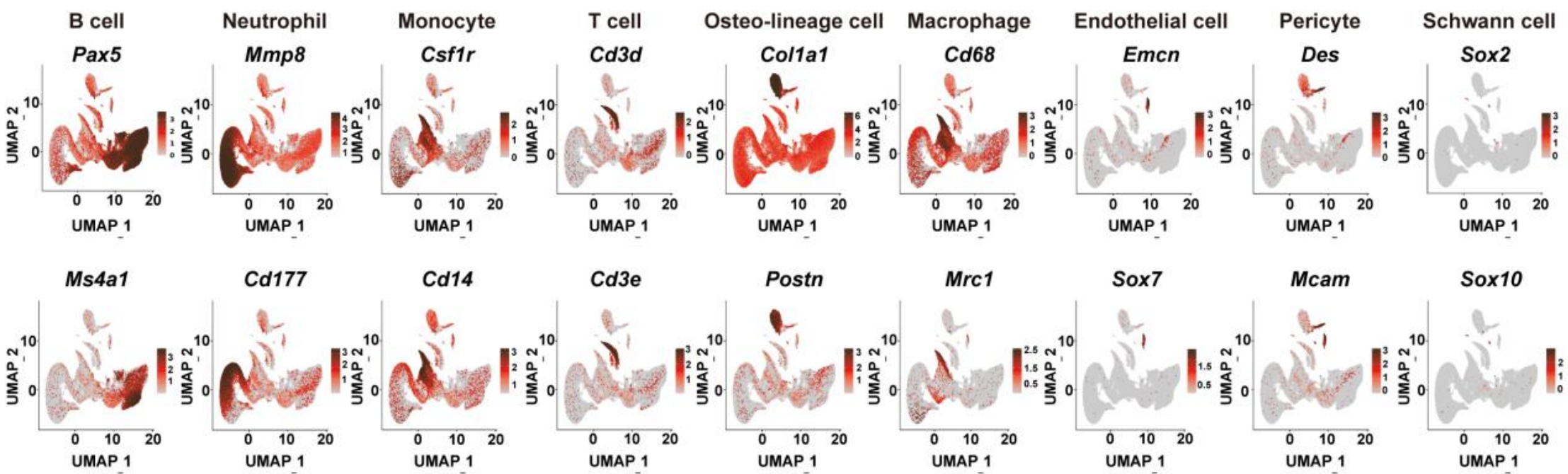

Supplementary Fig. 47. UMAP visualization showing the expression of cell-specific marker genes used to identify 9 distinct cell clusters: B cell (*Pax5, Ms4a1*), Neutrophil (*Mmp8, Cd177*), T cell (*Cd3d, Cd3e*), Monocyte (*Csf1r, Cd14*), Macrophage (*Cd68, Mrc1*), Osteo-lineage cell (*Col1a1, Postn*), Endothelial cell (*Emcn, Sox7*), Pericyte (*Des, Mcam*), and Schwann cell (*Sox2, Sox10*).

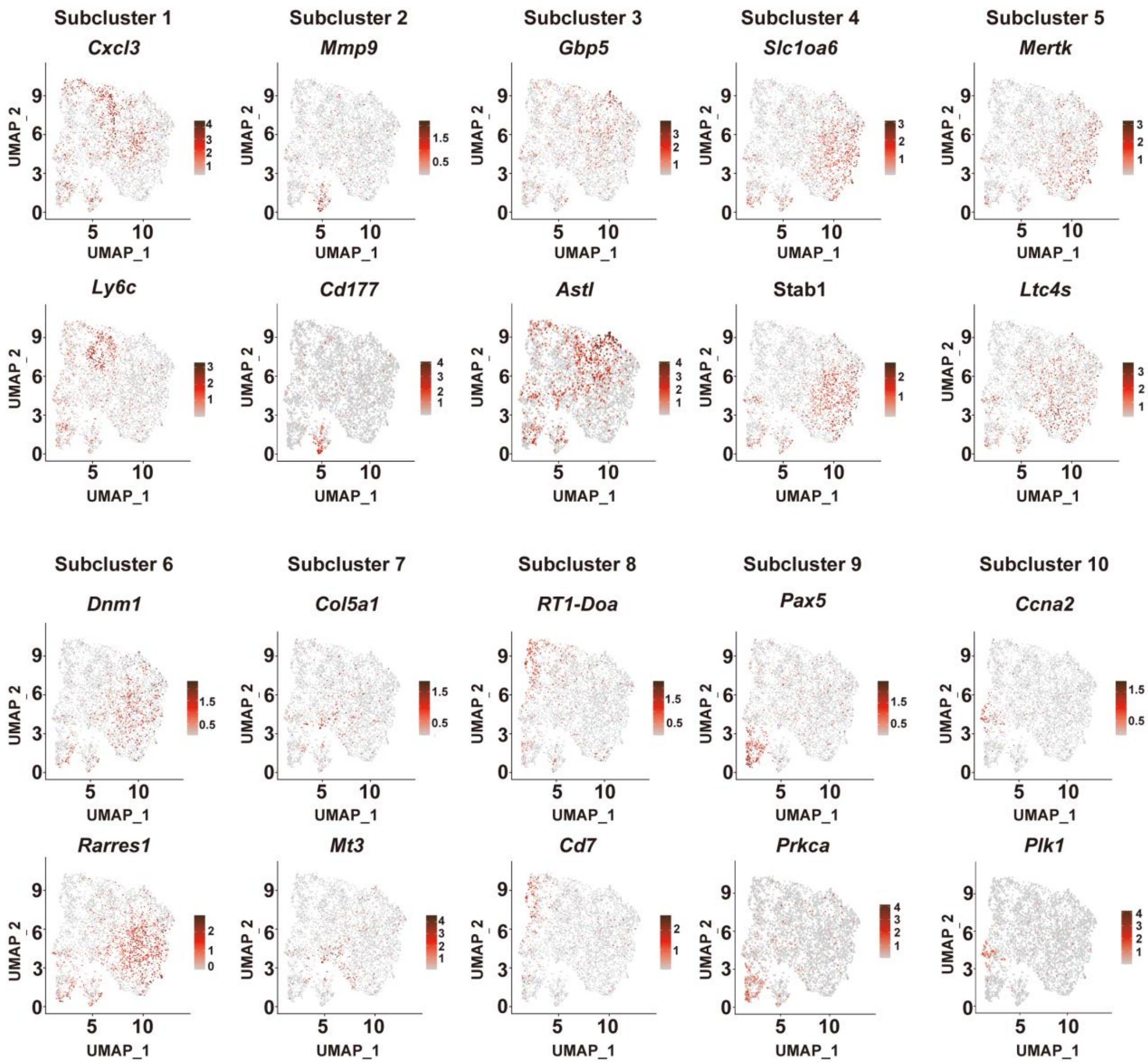

Supplementary Fig. 48. UMAP visualization showing the expression of cell-specific marker genes used to identify 10 macrophage subclusters: Subcluster 1 (*Cxcl3, Ly6c*), Subcluster 2 (*Mmp9, Cd177*), Subcluster 3 (*Gbp5, Astl*), Subcluster 4 (*Slc10a6, Stab1*), Subcluster 5 (*Mertk, Ltc4s*), Subcluster 6 (*Dnm1, Rarres1*), Subcluster 7 (*Col5a1, Mt3*), Subcluster 8 (*RT1-Doa, Cd7*), Subcluster 9 (*Pax5, Prkca*), and Subcluster 10 (*Ccna2, Plk1*).

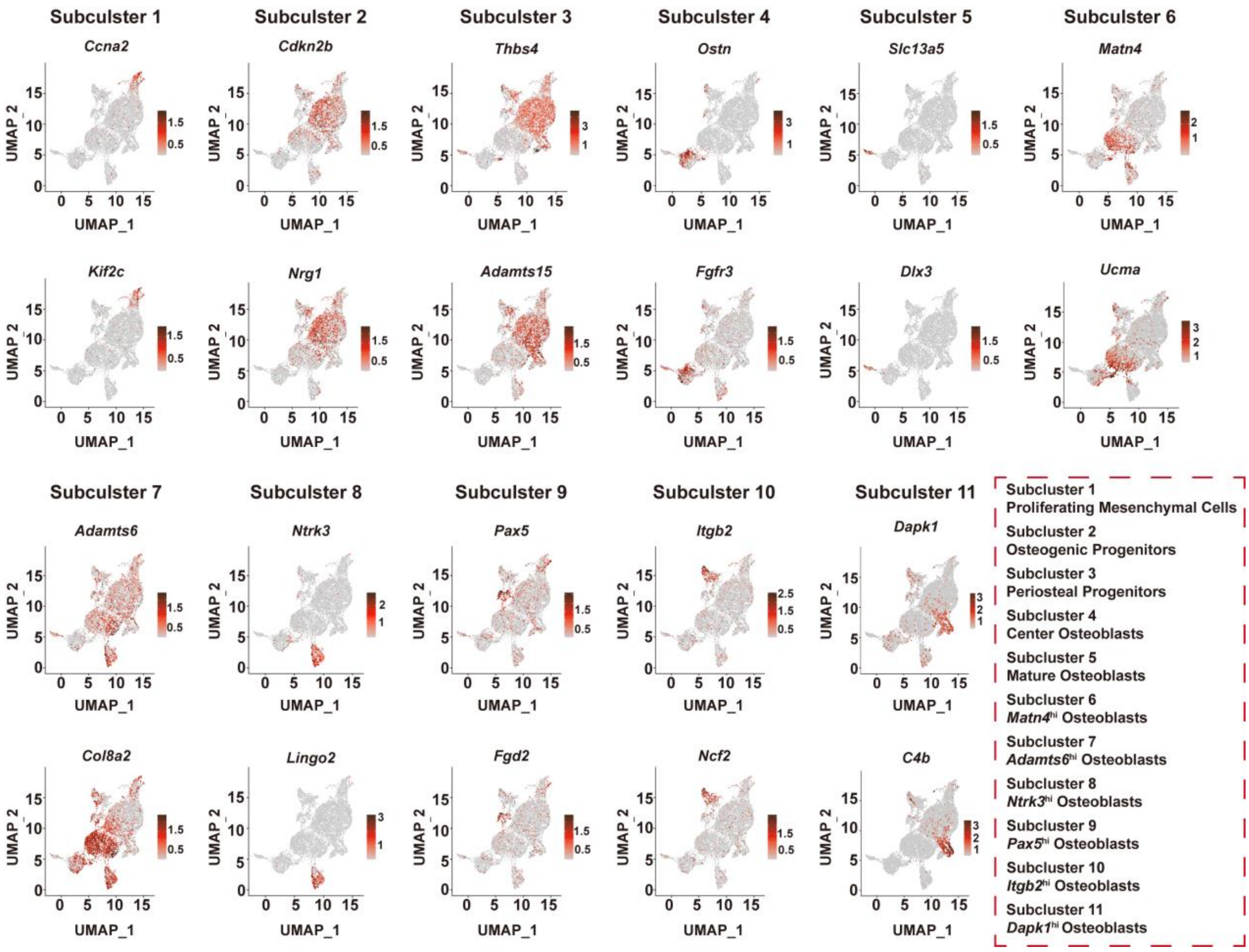

Supplementary Fig. 49. UMAP visualization showing the expression of cell-specific marker genes used to identify Osteo-lineage cells (OLCs) subclusters: Subcluster 1 (*Ccna2, Kif2c*), Subcluster 2 (*Cdkn2b, Nrg1*), Subcluster 3 (*Thbs4, Admts15*), Subcluster 4 (*Ostn, Fgfr3*), Subcluster 5 (*Slc13a5, Dlx3*), Subcluster 6 (*Matn4, Ucma*), Subcluster 7 (*Adamts6, Col8a2*), Subcluster 8 (*Ntrk3, Lingo2*), Subcluster 9 (*Pax5, Fgd2*), Subcluster 10 (*Itgb2, Ncf2*), and Subcluster 11 (*Dapk1, C4b*).

Supplementary Table 1. Information of antibodies used in this study

Antibody	Company, Catalog	Dilution
CD86	Biorbyt, orb500820-cf594	1:200
CD163	Biorbyt, 1333718	1:200
CD163	Biorbyt, orb612214	1:100
CD68	Abcam, ab125212	1:200
VEGF	Invitrogen, JH121	1:300
GAP43	Beyotime, AG1998	1:400
NGF	Invitrogen, SI79-01	1:200
S100-β	Proteintech, 15146-1-AP	1:100
CD31	Thermo Fisher, 14-0311-82	1:100
Emcn	Bioss, bs-5884R-PE	1:500
CGRP	Beyotime, AF6495	1:200
p-PI3k	Thermo Fisher, PA5-104853	1:300
p-Akt	Sigma-Aldrich, WH0000207M3	1:100
Runx2	Thermo Fisher, H00000860-M01	1:200
OCN	Proteintech, 23418-1-AP	1:200
Tubb3	Proteintech, 66375-1-lg	1:100
Osterix	Invitrogen, PSH07-29	1:100
NF 200	Thermo Fisher, 711025	1:500
Goat Anti-*Rabbit* IgG (Alexa Fluor 488)	Abcam, ab150077	1:500
Donkey Anti-*Rat* IgG (Alexa Fluor 488)	Thermo Fisher, A21208	1:500
Goat Anti-*Mouse* IgG (Alexa Fluor 488)	Thermo Fisher, A11001	1:500
Goat Anti-*Rabbit* IgG (Alexa Fluor 568)	Abcam, ab175471	1:500
Donkey Anti-*Rat* IgG (Alexa Fluor 647)	Abcam, ab150155	1:500
Goat Anti-*Rabbit* IgG (Alexa Fluor 647)	Abcam, ab150083	1:500
Goat Anti-*Mouse* IgG (Alexa Fluor 647)	Abcam, ab150115	1:500

Supplementary Table 2. Primer sequences for qPCR

Primer	Sequence (5' → 3')	
	**Forward**	**Reverse**
H-OCN	TCTACCAGTTGCAGCCTGAC	GTTCCCTTCCTCCTTGATTT
H-RUNX2	GACTGTGGTTACCGTCATGGC	ACTTGGTTTTTCATAACAGCGGA
H-OPN	CCAAGTAAGTCCAACGAAAG	ATGTCTGCTCCTGTAGTGG
H-COL1A1	TTCTATTGGTCCCGTCGGT	GCTGAGTCTCAGGTCGCG
H-GAPDH	GCCTGGTCACCAGGGCTGC	TGCTAAGCAGTTGGTGGTGCA
M-Tnf	GACGTGGAACTGGCAGAAGAG	TTGGTGGTTTGTGAGTGTGAG
M-Ccl2	TTAAAAACCTGGATCGGAACCAA	GCATTAGCTTCAGATTTACGGGT
M-Il6	TAGTCCTTCCTACCCCAATTTCC	TTGGTCCTTAGCCACTCCTTC
M-Arg1	CTGGCAGTTGGAAGCATCTCT	GTGAGCATCCACCCAAATGAC
M-Mrc1	CTCTGTTCAGCTATTGGACGC	TGGCACTCCCAAACATAATTTGA
M-Il10	CTTACTGACTGGCATGAGGATCA	GCAGCTCTAGGAGCATGTGG
M-Gapdh	ACCCAGAAGACTGTGGATGG	TTCAGCTCAGGGATGACCTT
H-VEGF	CTACCTCCACCATGCCAAGT	CACACAGGATGGCTTGAAGA
H-HIF-1α	CTACAAGAAACCGCCTATGACG	GGCTCATAACCCATCAACTCAG
R-Ngf	TGCATAGCGTAATGTCCATGTTG	TGTGTCAAGGGAATGCTGAA
R-Bdnf	CGTGGGGAGCTGAGCGTGTG	GCCCCTGCAGCCTTCCTTC
R- Gapdh	ATGACTCTACCCACGGCAAG	CATACTCAGCACCAGCATCAC

References

1. Yuan, S. *et al.* Sword and Board in One: A Bioinspired Nanocomposite Membrane for Guided Bone Regeneration. *Advanced Materials*, 2504577 (2025).
2. Chen, W. *et al.* Harmonizing thickness and permeability in bone tissue engineering: a novel silk fibroin membrane inspired by spider silk dynamics. *Advanced Materials* **36**, 2310697 (2024).
3. Yang, D. *et al.* Immunomodulatory multifunctional Janus collagen-based membrane for advanced bone regeneration. *Nature Communications* **16**, 4264 (2025).
4. Feng, Y. *et al.* Biomimetic lamellar chitosan scaffold for soft gingival tissue regeneration. *Advanced Functional Materials* **31**, 2105348 (2021).
5. Zhao, Y. *et al.* Janus membrane with intrafibrillarly strontium-apatite-mineralized collagen for guided bone regeneration. *ACS nano* **18**, 7204-7222 (2024).
6. Wu, S. *et al.* All-in-one porous membrane enables full protection in guided bone regeneration. *Nature Communications* **15**, 119 (2024).
7. Zhang, K.-R. *et al.* Multifunctional bilayer nanocomposite guided bone regeneration membrane. *Matter* **1**, 770-781 (2019).
8. Wang, Q. *et al.* A hierarchical Janus nanofibrous membrane combining direct osteogenesis and osteoimmunomodulatory functions for advanced bone regeneration. *Advanced Functional Materials* **31**, 2008906 (2021).
