# Supplementary material for: Unified bilayer membranes with mechanically reinforced interface for stage-adaptive bone regeneration
Source: Nat Commun. 2026 Jun 30;17:8193. doi: 10.1038/s41467-026-74932-x (PMC13462358; doi:10.1038/s41467-026-74932-x)
Supplement: Supplementary file 2 [file 41467_2026_74932_MOESM2_ESM.pdf]

Description of Additional Supplementary file

Supplementary Movie 1. Lifting tests of the CSO1M3 membrane.
