# Supplementary material for: Unified bilayer membranes with mechanically reinforced interface for stage-adaptive bone regeneration
Source: Nat Commun. 2026 Jun 30;17:8193. doi: 10.1038/s41467-026-74932-x (PMC13462358; doi:10.1038/s41467-026-74932-x)
Supplement: Supplementary file 3 [file 41467_2026_74932_MOESM4_ESM.pdf]

nature portfolio

Corresponding author(s): Yuanchi Zhang, Yuxiao Lai

Last updated by author(s): Jun 11, 2026

# Reporting Summary

Nature Portfolio wishes to improve the reproducibility of the work that we publish. This form provides structure for consistency and transparency in reporting. For further information on Nature Portfolio policies, see our Editorial Policies and the Editorial Policy Checklist.

## Statistics

For all statistical analyses, confirm that the following items are present in the figure legend, table legend, main text, or Methods section.

| n/a | Confirmed | |
|---|---|---|
| ☐ | ☒ | The exact sample size ($n$) for each experimental group/condition, given as a discrete number and unit of measurement |
| ☐ | ☒ | A statement on whether measurements were taken from distinct samples or whether the same sample was measured repeatedly |
| ☐ | ☒ | The statistical test(s) used AND whether they are one- or two-sided<br>*Only common tests should be described solely by name; describe more complex techniques in the Methods section.* |
| ☒ | ☐ | A description of all covariates tested |
| ☐ | ☒ | A description of any assumptions or corrections, such as tests of normality and adjustment for multiple comparisons |
| ☐ | ☒ | A full description of the statistical parameters including central tendency (e.g. means) or other basic estimates (e.g. regression coefficient) AND variation (e.g. standard deviation) or associated estimates of uncertainty (e.g. confidence intervals) |
| ☐ | ☒ | For null hypothesis testing, the test statistic (e.g. $F$, $t$, $r$) with confidence intervals, effect sizes, degrees of freedom and $P$ value noted<br>*Give P values as exact values whenever suitable.* |
| ☒ | ☐ | For Bayesian analysis, information on the choice of priors and Markov chain Monte Carlo settings |
| ☒ | ☐ | For hierarchical and complex designs, identification of the appropriate level for tests and full reporting of outcomes |
| ☒ | ☐ | Estimates of effect sizes (e.g. Cohen's $d$, Pearson's $r$), indicating how they were calculated |

*Our web collection on statistics for biologists contains articles on many of the points above.*

## Software and code

Policy information about availability of computer code

| | |
|---|---|
| Data collection | Data were collected with various laboratory instruments and equipments. Such as: a FTIR spectrometer (Thermo Fisher, Nicolet 6700, USA), a XPS spectrometer (Thermo Fisher Scientific, USA), a LabRAM ARAMIS system (Horiba-Jobin Yvon, France), a SEM (Sigma 300, Carl Zeiss, Germany), an EDS spectrometer (XFlash 6130, Bruker, Germany), a 3D optical profilometry (Bruker, ContourGT-K, Germany), a static contact angle meter (Dataphysics, OCA, 15EC, Germany), an electronic universal testing machine (TSE503C, WANCE, China), a pH meter (S220 SevenCompact™, Mettler Toledo, Switzerland), a UV-2600 spectrophotometer (Shimadzu, Japan), inductively coupled plasma optical emission spectroscopy (ICP-OES, 700 Series, Agilent Technologies, USA), an iCAP 7000 Series instrument (Thermo Fisher Scientific, USA), an automatic microplate reader (Epoch 2, BioTek, USA), a confocal laser scanning microscope (LSM 900, Zeiss, Germany), an Agilent 2100 Bioanalyzer (Agilent Technologies, USA), a QuantStudio 6 Real-Time PCR System (Thermo Fisher Scientific, USA), an HiSeq X Ten platform (Illumina, USA), a phase-contrast inverted microscope (CKX53, Olympus, Japan), a light microscopy (Olympus IX71, Japan), an NovaSeq 6000 (Illumina, San Diego, CA, USA), an RFLSI Laser Speckle Imaging System (RFLSI-ZW, Shenzhen Ruiward Life Technology, China), a micro-computed tomography scanner (Skyscan 1176, Bruker, Belgium), a Luna™ Automated Cell Counter (Logos Biosystems, Anyang, South Korea), a BGI DNBSEQ-T7 PE100 (MGI Tech Co., Ltd., Shenzhen, China). |
| Data analysis | ImageJ software (v.1.6.0), GraphPad Prism 8, Microsoft Excel 2013, OriginPro 2022, CTAnalyser (v.1.18.4.0), CTvox (v.3.3.1), Cell Ranger (v9.0.0), Seurat (v5.0), clusterProfiler (v.4.0), CytoTRACE (v0.3.3), Monocle 3 (v1.3.1), CellChat (v1.6.1), Blender 4.1, Adobe Illustrator 2019. |

For manuscripts utilizing custom algorithms or software that are central to the research but not yet described in published literature, software must be made available to editors and reviewers. We strongly encourage code deposition in a community repository (e.g. GitHub). See the Nature Portfolio guidelines for submitting code & software for further information.

## Data

Policy information about availability of data

All manuscripts must include a data availability statement. This statement should provide the following information, where applicable:

- Accession codes, unique identifiers, or web links for publicly available datasets
- A description of any restrictions on data availability
- For clinical datasets or third party data, please ensure that the statement adheres to our policy

All data generated in this study are provided in the main text and Supplementary Information. The RNA-seq and scRNA-seq raw data have been deposited respectively in the Genome Sequence Archive in National Genomics Data Center, China National Center for Bioinformation/Beijing Institute of Genomics, Chinese Academy of Sciences under the accession code "CRA035122 [https://ngdc.cncb.ac.cn/gsa/browse/CRA035122]" and under the accession code "CRA040859 [https://ngdc.cncb.ac.cn/gsa/browse/CRA040859]". Any additional requests for information can be directed to, and will be fulfilled by, the corresponding authors. Source data are provided with this paper.

## Research involving human participants, their data, or biological material

Policy information about studies with human participants or human data. See also policy information about sex, gender (identity/presentation), and sexual orientation and race, ethnicity and racism.

| | |
|---|---|
| Reporting on sex and gender | N/A |
| Reporting on race, ethnicity, or other socially relevant groupings | N/A |
| Population characteristics | N/A |
| Recruitment | N/A |
| Ethics oversight | N/A |

Note that full information on the approval of the study protocol must also be provided in the manuscript.

# Field-specific reporting

Please select the one below that is the best fit for your research. If you are not sure, read the appropriate sections before making your selection.

☒ Life sciences ☐ Behavioural & social sciences ☐ Ecological, evolutionary & environmental sciences

For a reference copy of the document with all sections, see nature.com/documents/nr-reporting-summary-flat.pdf

# Life sciences study design

All studies must disclose on these points even when the disclosure is negative.

| | |
|---|---|
| Sample size | Sample sizes were chosen on the basis of published literature on similar evaluations (for example, Nat Commun 15, 119 (2024)). For each experiment, we have adopted at least n=3 biological replicates to calculate the statistical value of each analysis and the exact sample sizes and statistical data for each experiment are reported in the figure legends. |
| Data exclusions | No data were excluded from the analyses. |
| Replication | All experiments were repeated at least three times with reproducibility, and all attempts at replication were successful. |
| Randomization | All the tests were performed with randomly allocated experimental groups. |
| Blinding | All measurements were conducted in a blinded fashion. |

# Reporting for specific materials, systems and methods

We require information from authors about some types of materials, experimental systems and methods used in many studies. Here, indicate whether each material, system or method listed is relevant to your study. If you are not sure if a list item applies to your research, read the appropriate section before selecting a response.

## Materials & experimental systems

| n/a | Involved in the study |
| --- | --- |
| ☐ | ☒ Antibodies |
| ☐ | ☒ Eukaryotic cell lines |
| ☒ | ☐ Palaeontology and archaeology |
| ☐ | ☒ Animals and other organisms |
| ☒ | ☐ Clinical data |
| ☒ | ☐ Dual use research of concern |
| ☒ | ☐ Plants |

## Methods

| n/a | Involved in the study |
| --- | --- |
| ☒ | ☐ ChIP-seq |
| ☒ | ☐ Flow cytometry |
| ☒ | ☐ MRI-based neuroimaging |

# Antibodies

Antibodies used

The following is a list of the primary antibodies used:
CD86 (Biorbyt, cat# orb500820-cf594, dilution 1:200, RRID:AB_3740115);
CD163 (Biorbyt, cat#orb1333718, dilution 1:200, RRID:AB_3750780);
CD163 (Biorbyt,cat# orb612214,dilution 1:100, RRID:AB_3750781);
CD68 (Abcam, cat# ab125212, dilution 1:200,RRID: AB_10975465);
VEGF (Invitrogen, cat# JH121, dilution 1:300, RRID:AB_3750782);
NGF (Invitrogen, cat# SI79-01, dilution 1:200, RRID: AB_3750783);
S100-β (Proteintech, cat# 15146-1-AP, dilution 1:100, RRID:AB_2254244);
Emcn (Bioss, cat# bs-5884R-PE, dilution 1:500, RRID:AB_11100214);
CGRP (Beyotime, cat# AF6495, dilution 1:200, RRID: AB_3750784);
p-PI3K (Thermo Fisher, cat# PA5-104853, dilution 1:300, RRID: AB_2816326);
p-Akt (Sigma-Aldrich, cat# WH0000207M3, dilution 1:100, RRID: AB_1839508 );
RUNX2 (Thermo Fisher, cat# H00000860-M01, dilution 1:200, RRID: AB_3750786 );
OCN (Proteintech, cat# 23418-1-AP, dilution 1:200,RRID: AB_2879275);
Osterix (Invitrogen, cat# PSH07-29,dilution 1:100, RRID: AB_3750787);
CD31 (Invitrogen, cat# 14-0311-82,dilution 1:100, RRID: AB_3750788);
Tubb3 (Proteintech, cat# 66375-1-lg, 1:100, RRID:AB_2814998);
NF200 (Thermo Fisher, cat# 711025,1:500 ,RRID: AB_2609477);
GAP43 (Beyotime, AG1988, cat#711025, 1:500, RRID: AB_3750790);
The secondary antibodies used were:
Goat Anti-Rabbit IgG, (Alexa Fluor 488), (Abcam, cat# ab150077, dilution 1:500 ,RRID: AB_2630356);
Donkey Anti-Rat IgG, (Alexa Fluor 488), (Thermo Fisher, cat# A21208, dilution 1:500, RRID:AB_2535794);
Goat Anti-Mouse IgG, (Alexa Fluor 488), (Thermo Fisher, cat# A11001, dilution 1:500, RRID:AB_2534069);
Goat Anti-Rabbit IgG, (Alexa Fluor 568), (Abcam, cat# ab175471, dilution 1:500,RRID: AB_2576207);
Donkey Anti-Rat IgG, (Alexa Fluor 647), (Abcam, cat# ab150155, dilution 1:500, RRID:AB_2813835);
Goat Anti-Rabbit IgG, (Alexa Fluor 647), (Abcam, cat# ab150083, dilution 1:500, RRID:AB_2714032);
Goat Anti-Mouse IgG, (Alexa Fluor 647), (Abcam, cat# ab150115, dilution 1:500, RRID:AB_2687948).

Validation

These antibodies have been validated by the suppliers and are reported to be suitable for use in immunofluorescence. All antibodies used in this study are sourced from commercial suppliers, and validation data can be found on the manufacturer's website using their respective catalogue numbers. The manufacturers include Biorbyt, Abcam, Invitrogen, Proteintech, Bioss, Beyotime, Thermo Fisher Scientific, and Sigma-Aldrich.

# Eukaryotic cell lines

Policy information about [cell lines and Sex and Gender in Research]

Cell line source(s)

The hBMSCs (Human Bone Marrow Mesenchymal Stem Cells) were purchased from Cyagen (Suzhou, China). Catalog number: HUXMA-01001.
Quality control:
1.The cells passed sterility testing for bacteria, fungi, mycoplasma and endotoxin.
2.The cells passed post-thaw viability testing, with a recovery viability of >80%.
3.The cells passed cell growth assessment, with a population doubling time of <72 h.
4.Flow cytometry analysis showed that the cells were positive for CD29, CD44, CD73 and CD105 (>70%), and negative for CD34, CD45 and CD11b (<5%).
5.The cells exhibited good multilineage differentiation potential and could be induced to differentiate into osteoblasts, adipocytes and chondrocytes.
The RAW264.7 (Murine Macrophage Cell Line) was purchased from ATCC (Shanghai, China). Catalog number: TIB-71. RRID:CVCL_0493.
The PC12 (Rat Pheochromocytoma Cell Line) was purchased from ATCC (Shanghai, China). Catalog number: CRL-1721. RRID:CVCL_0481.
The L929 (Mouse Fibroblast Cell Line) was purchased from ATCC (Shanghai, China). Catalog number: CCL-1.RRID: CVCL_0462.
The HUVECs (Human Umbilical Vein Endothelial Cells) were purchased from ATCC (Shanghai, China). Catalog number: CRL-1730, RRID: CVCL_2959.
The RSCs (Rat Schwann Cells) were purchased from iCell (Shanghai, China). Catalog number: RAT-iCell-n008.
Quality control:
1.The tissue was derived from normal brain tissue of experimental animals.

| | |
|---|---|
| | 2.Cell identification: positive for GFAP by immunofluorescence staining.<br>3.The cell purity was confirmed to be >90%.The cells were negative for HIV-1, HBV, HCV, mycoplasma, bacteria, yeast and fungi.<br>4.Cell growth pattern: long spindle-shaped and irregular cells, cultured as adherent cells.<br>The hPDSCs (Human periosteum-derived stem cells) were purchased from iCells (Shanghai, China). Catalog number: HUM-iCell-s050.<br>Quality control:<br>1.The cells passed sterility testing for bacteria, fungi, mycoplasma and endotoxin.<br>2.The cells passed post-thaw viability testing, with a recovery viability of >80%.<br>3.The cells passed cell growth assessment, with a population doubling time of <72 h.<br>4.Flow cytometry analysis showed that the cells were positive for CD29, CD44, CD73 and CD105 (>70%), and negative for CD34, CD45 and CD11b (<5%).<br>5.The cells exhibited good multilineage differentiation potential and could be induced to differentiate into osteoblasts, adipocytes and chondrocytes. |
| Authentication | All cell line characterization was conducted by the respective companies to verify the authenticity of the cell lines. |
| Mycoplasma contamination | All cell lines were tested and not contaminated with mycoplasma. |
| Commonly misidentified lines<br>(See ICLAC register) | No commonly misidentified lines were used in this study. |

## Animals and other research organisms

Policy information about studies involving animals; ARRIVE guidelines recommended for reporting animal research, and Sex and Gender in Research

| | |
|---|---|
| Laboratory animals | A total of 76 male SD rats aged 7–8 weeks used for the cranial defect model, transcriptomic analysis and single-cell RNA sequencing were purchased from Guangdong Weitong LiHua Experimental Animal Technology Co., Ltd. (China). Additionaly, 6 Male SD rats (7-8 weeks old) for subcutaneous antimicrobial experiments were procured from Shanghai Southern Model Biological Co., Ltd. (China). |
| Wild animals | The study did not involve wild animals. |
| Reporting on sex | The findings of the experiments apply to both females and males. |
| Field-collected samples | This study did not involve samples collected from the field. |
| Ethics oversight | In vivo rat critical size cranial defect experimental procedures and protocols were approved by the Ethics Review Committee of the Shenzhen Institutes of Advanced Technology (IACUC Number: SIAT-IACUC-240326-YGS-ZYC-A2527). In vivo antibacterial experiments were carried out according to the institutional guidelines and approved by Animal Care and Use Committee (IACUC) in Shanghai Yishang Biotechnology Co., Ltd (IACUC-2025-1-037). |

Note that full information on the approval of the study protocol must also be provided in the manuscript.

## Plants

| | |
|---|---|
| Seed stocks | N/A |
| Novel plant genotypes | N/A |
| Authentication | N/A |
