# Supplementary material for: Unified bilayer membranes with mechanically reinforced interface for stage-adaptive bone regeneration
Source: Nat Commun. 2026 Jun 30;17:8193. doi: 10.1038/s41467-026-74932-x (PMC13462358; doi:10.1038/s41467-026-74932-x)
Supplement: Supplementary file 4 [file 41467_2026_74932_MOESM5_ESM.pdf]

nature portfolio

Peer Review File

Unified Bilayer Membranes with Mechanically Reinforced Interface for Stage-adaptive Bone Regeneration

Corresponding Author: Professor Yuxiao Lai

This file contains all reviewer reports in order by version, followed by all author rebuttals in order by version.

Version 0:

Reviewer comments:

Reviewer #1

(Remarks to the Author)
This manuscript presents a well-designed and comprehensive study on the development of a unified bilayer membrane (CSO_1M_3) for stage-adaptive bone regeneration. The authors engineered the composite membrane through a dual cross-linking strategy involving both Schiff-base covalent bonding and a phenolic coordination system, to enhance its mechanical performance and structural integrity. It is interesting that the authors develop a homogeneous isomeric in-situ fabrication approach to effectively unify the two disparate layers of the composite membrane. The work is innovative, combining mechanical reinforcement with sequential bioactive effects to address key challenges in guided bone regeneration. In addition, the findings are supported by a series of in vitro and in vivo experiments to suggest improvements at various stages. Therefore, this manuscript can be accepted for publication in Nature Communications after minor revisions to clarify certain points and enhance the presentation.

1. The proposed homogeneous isomeric in-situ forming strategy is important for preparing the unified structure of the composite membrane, please add a discussion about which materials are suitable for this method.
2. The authors presented the SEM image of the CSO_1M_3 membrane after two months of degradation to show its long-term structural sustainability (Supplementary Fig. 6). To clarify this, the authors were suggested to provide quantitative analysis after degradation to describe the mechanical stability of the composite membrane.
3. Given that in vivo studies have showed the immunofluorescence staining of Runx2 and OCN images, the corresponding immunofluorescence staining images using osteogenic-related cells (e.g. hBMSCs) in vitro were suggested to supplement for better overall understanding.
4. The study attributes M2 macrophage polarization to the synergistic effect of OTA and Mg^{2+}. Could the authors clarify the dominant factor in this synergy? Is there any potential competition between the ROS-scavenging activity of OTA and the signaling pathways modulated by Mg^{2+}? It would be helpful to discuss whether the anti-inflammatory efficacy is primarily driven by ROS scavenging or by direct signaling pathway modulation.
5. Some figures and figure captions should be modified to meet guidelines for Nature Communications, e.g. the P values in the figure, adjustments for multiple comparisons.
6. The language should be double checked throughout the text, e.g. “might be attribute” should be “might be attributed”.

Reviewer #2

(Remarks to the Author)
In this study, the authors reported a unified bilayer membranes (denoted CSO1M3) with a reinforced dual-crosslinking interface between barrier and porous layers, fabricated via a homogeneous isomeric in-situ strategy. The membrane’s superior mechanical strength (~157 MPa in dry state and ~8.6 MPa in wet state) ensures its long-term degradation stability and bioactivity in vivo. Through systematic in vitro and in vivo experiments, the authors further evaluated stage-adaptive regulation of the unified bilayer membranes across the bone-repairing cascade: antibacterial (Stage I), immunomodulation (Stage II), neovascularization (Stage III) and osteogenesis (Stage IV). Bulk RNA-seq analyses were performed at Stage I and Stage IV to compare the CSO1M3 and control groups to identify differentially expressed genes, which were enriched in key biological functions and pathways. While these bulk transcriptomic insights are valuable, it is important to note that bulk RNA-seq captures only the average transcriptional signal from a heterogeneous tissue mixture. This approach inevitably masks the distinct contributions and dynamic responses of individual cell subpopulations—such as specific macrophage

phenotypes, osteoprogenitor subsets, endothelial subtypes, and neural cells—that collectively drive the regenerative process. In addition, this study fails to capture the continuous, time-resolved transitions in cell states, and couldn't delineate the cellular trajectories that illustrate the progression of regeneration over time.

The authors provided a clear schematic illustrating the in vivo experimental timeline from 1 to 8 week across the bone-repairing cascades, which offers an excellent framework for deeper cellular investigation. To address the existing gaps in cellular heterogeneity, it is recommended to employ single-cell sequencing methodologies in subsequent validation. Performing single-cell RNA sequencing on cells isolated from the implantation site at the 8 time points would allow for an unbiased decoding the complex cellular landscape. This high-resolution approach would enable the authors to: 1) identify the specific immune cell subsets (e.g., M2a, M2c macrophages) recruited or modulated by the CSO1M3; 2) delineate the differentiation trajectories of osteoblasts, chondrocytes and other cell lineages using trajectory inference tools such as monocle3 or PAGA; and 3) reconstruct the cell-cell communication networks that are altered by the implant, utilizing tools like CellChat or CellPhoneDB to predict key ligand-receptor interaction within the regenerating niche. In addition, spatial transcriptomics (e.g., Visium) is advised to clarify the three-dimensional spatial relationships between nerves, vessels, and new bone, enabling direct analysis of co-localization patterns.

Reviewer #3

(Remarks to the Author)
In this study, the authors propose a novel periosteum-inspired composite membrane comprised of chitosan (CS), oxidized tannic acid (OTA) and magnesium oxide (MgO) nanoparticles, to mechanically protect and adaptively regulate the bone regeneration process. As natural periosteum's structure, the composite membrane was constructed with unified barrier layer and porous layer through a homogeneous isomeric in-situ fabrication strategy. This unified bilayer membrane was demonstrated to sequentially kill bacteria, regulate immune responses, improve neurovascular ingrowth and enhance osteogenesis through in vitro and in vivo studies. The authors also explored and discussed the regulatory mechanisms underlying stage-adaptive bone regeneration of the composite membrane. The manuscript is generally well-written, logically structured, and supported by extensive data. Therefore, the manuscript is recommended for publication after moderate revision. Some comments and suggestions are listed as below:

1. Bioactive implants usually work in a physiologically wet environment, so it is impressive that the proposed membrane has a good mechanical strength (~8.6 MPa) in wet state. The reader wonders what the strength of the membrane will be after biodegradation for 8 weeks (the period for bone regeneration in this work). And whether the strength is sufficient to maintain suturing with the tissues or not.
2. Since the dual-crosslinking network is important for the composite membrane, it would be helpful to provide a schematic figure to describe its preparation process.
3. The authors propose triple antibacterial mechanisms by the unified composite membrane. Please add a brief discussion on the relative contributions of these mechanisms, highlighting which might be the most critical.
4. Neurovascularization (Stage III) at the bone defect site seems to serve as a linking stage during the bone regeneration process. Please explain the effects of both nerve regeneration and angiogenesis on the ultimate bone repair.

Version 1:

Reviewer comments:

Reviewer #1

(Remarks to the Author)
The authors have well addressed my comments.

Reviewer #2

(Remarks to the Author)
The authors have largely addressed my concerns.

Reviewer #3

(Remarks to the Author)
I have no further question on this manuscript, it can be accepted for publication at its current status.

Open Access This Peer Review File is licensed under a Creative Commons Attribution 4.0 International License, which permits use, sharing, adaptation, distribution and reproduction in any medium or format, as long as you give appropriate credit to the original author(s) and the source, provide a link to the Creative Commons license, and indicate if changes were made.
In cases where reviewers are anonymous, credit should be given to 'Anonymous Referee' and the source.
The images or other third party material in this Peer Review File are included in the article's Creative Commons license, unless indicated otherwise in a credit line to the material. If material is not included in the article's Creative Commons license and your intended use is not permitted by statutory regulation or exceeds the permitted use, you will need to obtain permission directly from the copyright holder.
To view a copy of this license, visit https://creativecommons.org/licenses/by/4.0/

Response to Reviewers' Comments

Reviewer #1

General Comments:

This manuscript presents a well-designed and comprehensive study on the development of a unified bilayer membrane (CSO_1M_3) for stage-adaptive bone regeneration. The authors engineered the composite membrane through a dual cross-linking strategy involving both Schiff-base covalent bonding and a phenolic coordination system, to enhance its mechanical performance and structural integrity. It is interesting that the authors develop a homogeneous isomeric in-situ fabrication approach to effectively unify the two disparate layers of the composite membrane. The work is innovative, combining mechanical reinforcement with sequential bioactive effects to address key challenges in guided bone regeneration. In addition, the findings are supported by a series of in vitro and in vivo experiments to suggest improvements at various stages. Therefore, this manuscript can be accepted for publication in Nature Communications after minor revisions to clarify certain points and enhance the presentation.

Response:

We would like to thank the reviewer for giving positive comments and insightful advice to help us improve the manuscript.

Comments:

1. The proposed homogeneous isomeric in-situ forming strategy is important for preparing the unified structure of the composite membrane, please add a discussion about which materials are suitable for this method.

Response:

Thanks for the reviewer's comment. In this study, the bilayer composite membranes were fabricated using the CS, OTA and MgO nanoparticles. The homogeneous isomeric in-situ forming strategy was developed for eliminating interfacial discontinuities between layers of the membranes to enhance their structural uniformity and mechanical

reliability throughout the bone repair process. The discussion related other suitable materials has been added (Refer to Page 29) and highlighted in our revised manuscript.

Revision:

[Page 29, Paragraph 1]

"This strategy can also be extended to other material systems. Specifically, natural polysaccharides (e.g. chitosan, sodium alginate, hyaluronic acid and gelatin) are the primary choice for the precursor solution due to their good biocompatibility, tunable solubility, and ability to undergo in-situ crosslinking/phase transformation. Additionally, polyphenol derivatives (e.g. OTA and oxidized epigallocatechin gallate) act as dual-functional crosslinkers that strengthen the bilayer interface through covalent and non-covalent interactions. Besides, bioactive inorganic/organic nanoparticles (e.g. MgO, CaO and hydroxyapatite) can be incorporated as reinforcing fillers to enhance mechanical performance and enable sustained release of bioactive ions (e.g. Mg^{2+}, Ca^{2+}). By adjusting these material combinations, the strategy could also be used to fabricate unified bilayer membranes for repairing damaged skin, cartilage and other tissues."

2. The authors presented the SEM image of the CSO_1M_3 membrane after two months of degradation to show its long-term structural sustainability (Supplementary Fig. 6). To clarify this, the authors were suggested to provide quantitative analysis after degradation to describe the mechanical stability of the composite membrane.

Response:

We thank for the reviewer's comment. The qualitative and quantitative analysis of the structural stability of the composite membrane after degradation for 8 weeks have been supplemented as shown in Fig. R1 (Refer to Supplementary Fig. 7, Page 6 of Supplementary Information). The description has also been added and highlighted in our revised manuscript (Refer to Page 9 of Revision).

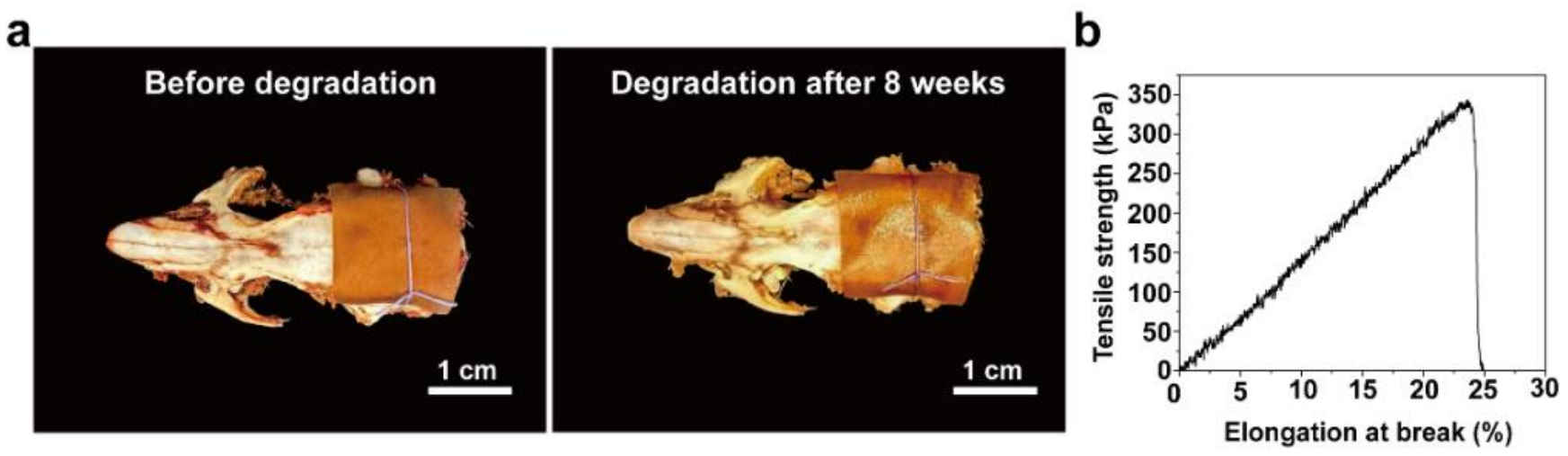

Fig. R1. The qualitative (a) and quantitative (b) analysis of the structural stability of the CSO_1M_3 membrane after degradation for 8 weeks. $n = 3$ samples per group.

Revision:

[Page 9, Paragraph 2]

"Macroscopic observations confirmed the shape stability of the CSO_1M_3 membranes on the rat skull after 8 weeks of degradation, with a corresponding residual tensile strength of ~300 kPa (Supplementary Fig. 7). This mechanical performance could ensure stable tissue suturing for structural support during bone repair process."

3. Given that in vivo studies have showed the immunofluorescence staining of Runx2 and OCN images, the corresponding immunofluorescence staining images using osteogenic-related cells (e.g. hBMSCs) in vitro were suggested to supplement for better overall understanding.

Response:

We appreciate the reviewer's helpful suggestion. The in vivo immunofluorescence staining of Runx2 and OCN images have been supplemented as shown in Fig. R2 (Refer to Supplementary Fig. 35, Page 21 of Supplementary Information). The discussion has also been added and highlighted in our revised manuscript (Refer to Page 24 of Revision).

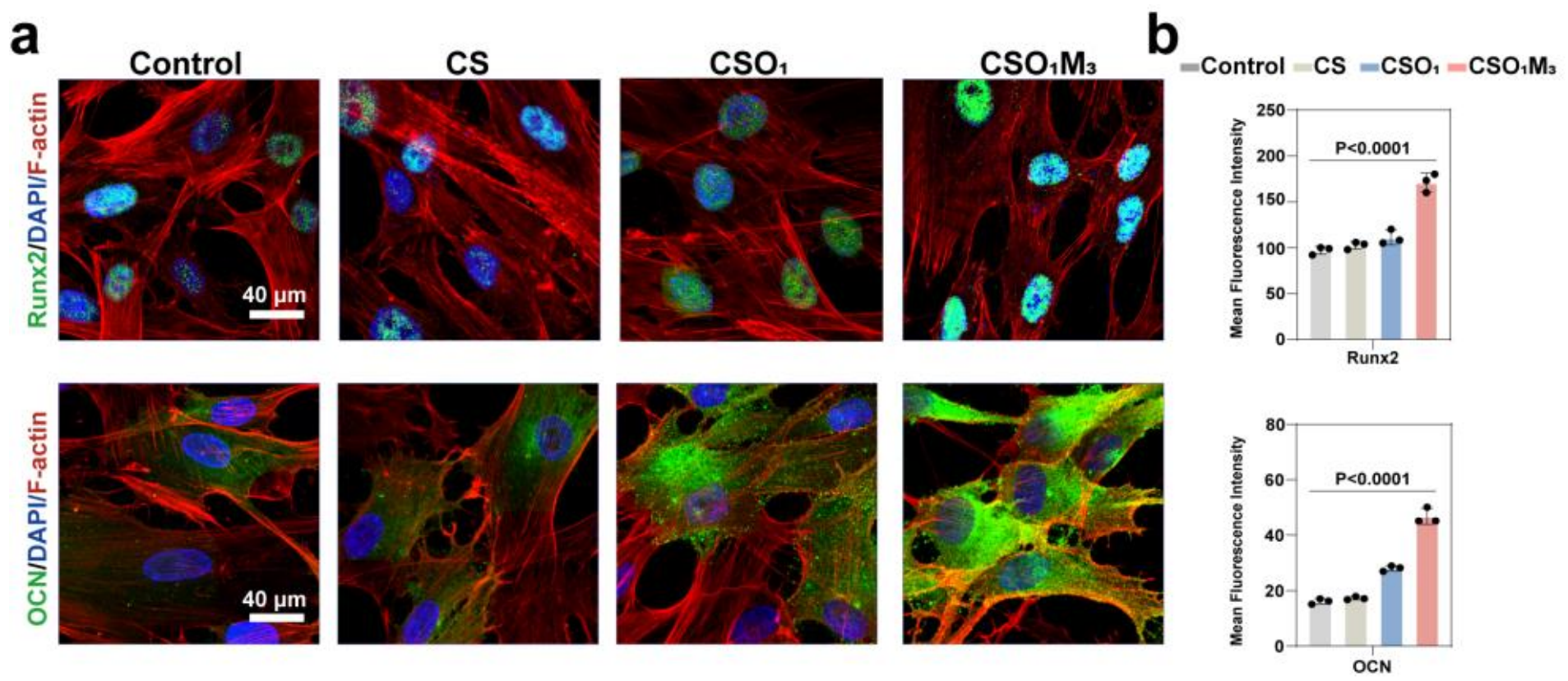

Fig. R2. Supplementary Fig. 35. Representative IF staining of Runx2 (a) and OCN (b) images of

the hBMSCs co-cultured in different groups for 7 and 14 days, respectively. Values in (b) represent the mean and standard deviation ($n = 3$ samples per group). Statistical analyses were performed by one-way ANOVA followed by Tukey's post hoc test. $p < 0.05$ was considered statistically significant differences between the compared groups.

Revision:

[Page 24, Paragraph 1]

"IF staining of Runx2 and OCN images presented significantly enhanced fluorescence intensities in the CSO_1M_3 group compared to other groups, confirming the upregulated expression of these osteogenic proteins (Supplementary Fig. 35)."

4. The study attributes M2 macrophage polarization to the synergistic effect of OTA and Mg^{2+}. Could the authors clarify the dominant factor in this synergy? Is there any potential competition between the ROS-scavenging activity of OTA and the signaling pathways modulated by Mg^{2+}? It would be helpful to discuss whether the anti-inflammatory efficacy is primarily driven by ROS scavenging or by direct signaling pathway modulation.

Response:

We thank for the reviewer's comment. Based on the results in Fig. R3 (Refer to Fig. 4c, Page 17 of Revision) and Fig. R4 (Refer to Supplementary Fig. 17, Page 11 of Supplementary Information), the effects between the ROS-scavenging activity of OTA and the signaling pathways modulated by Mg^{2+} are complementary rather than competitive. Compared to the control and CS groups with LPS, the CSO_1 and CSO_1M_3 groups significantly downregulated CD86 expression while upregulating CD163 expression, indicating high promotion of M2 macrophage polarization. Particularly, the CSO_1M_3 membranes could further reduce CD86 expression by ~56% and increasing CD163 expression by ~104% compared to those of the CSO_1 membranes. The ROS-scavenging activity of the polyphenol derivatives has been proved in previous publications (*Angewandte Chemie International Edition, 2026, 65, e13698*). In this work, OTA could create a favorable microenvironment by scavenging localized reactive oxygen species, which further improved the immunomodulatory efficiency of Mg^{2+}. Therefore, this enhanced M2 polarization was attributed to the synergistic immunomodulatory effects of OTA and Mg^{2+} released from the CSO_1M_3 matrix. The

discussion has also been added and highlighted in our revised manuscript (Refer to Page 15 of Revision).

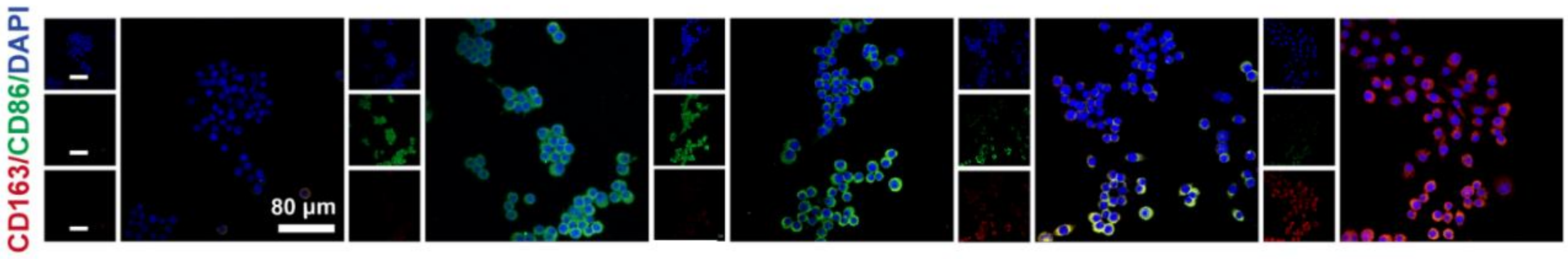

Fig. R3. Representative IF staining of CD86 (M1 marker, red) and CD163 (M2 marker, green) images of RAW 264.7 cells co-cultured with the membranes under no or LPS stimulation after 48 hours.

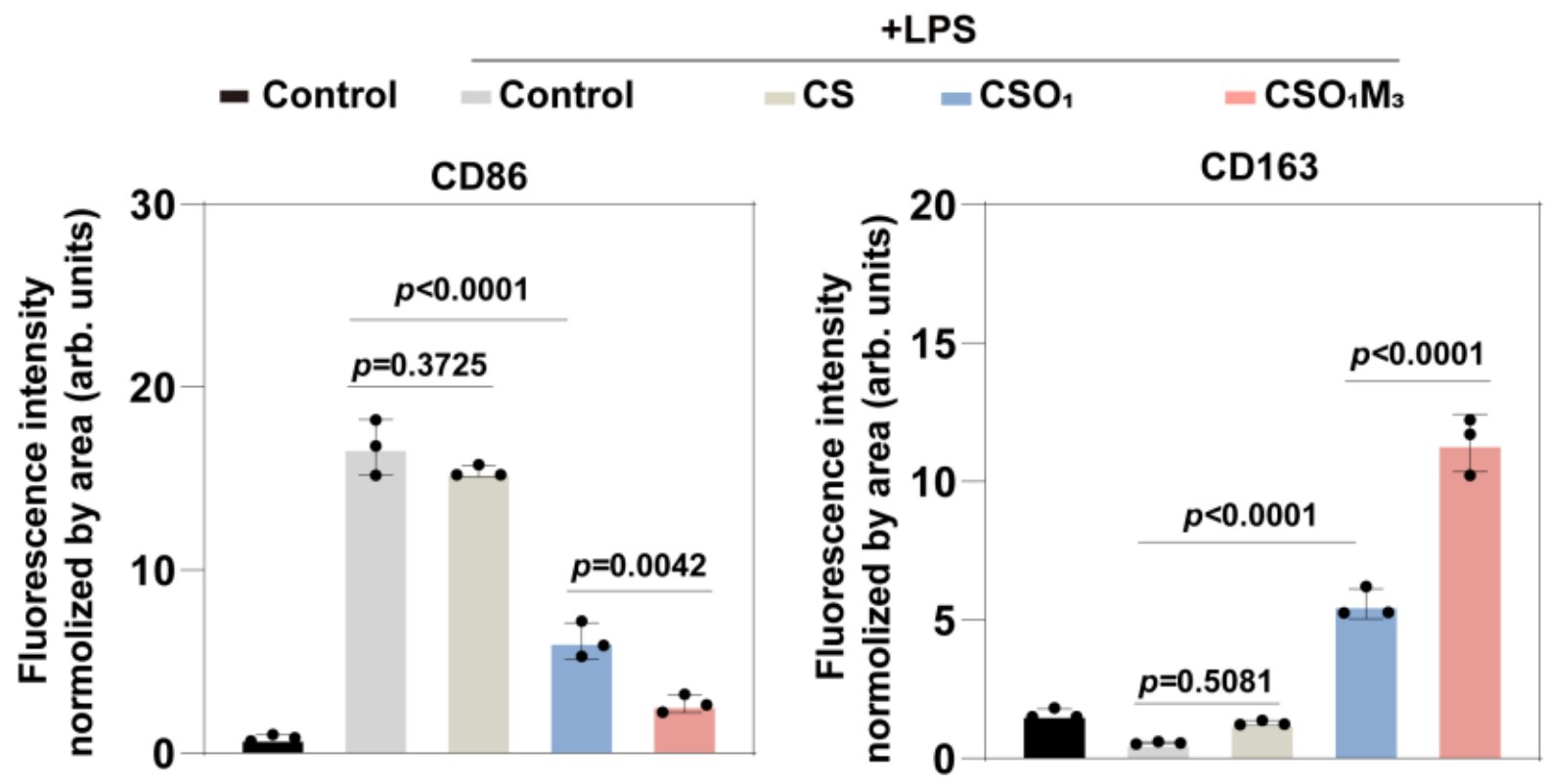

Fig. R4. Quantitative analysis of the immunofluorescence (IF) staining images for CD86 (M1 marker) and CD163 (M2 marker) expression after 48 hours of co-culture with different composite membranes. Values represent the mean and standard deviation ($n = 3$ samples per group). Statistical analyses were performed using one-way ANOVA followed by Tukey's post hoc test. No adjustments were made for multiple comparisons. $p < 0.05$ was considered statistically significant differences between the compared groups.

Revision:

[Page 15, Paragraph 1]

"OTA could create a favorable microenvironment by scavenging localized reactive oxygen species, which further improved the immunomodulatory efficiency of Mg^{2+}. Therefore, this enhanced M2 polarization was attributed to the synergistic immunomodulatory effects of OTA and Mg^{2+} released from the CSO_1M_3 matrix."

5. Some figures and figure captions should be modified to meet guidelines for Nature Communications, e.g. the P values in the figure, adjustments for multiple comparisons.

Response:

We really appreciate the reviewer for pointing out this. The format of the figures and figure captions in the revised manuscript has been supplemented and highlighted.

6. The language should be double checked throughout the text, e.g. "might be attribute" should be "might be attributed".

Response:

We thank for the reviewer's kind reminder. The language of the manuscript has been revised and polished in the revised version.

Reviewer #2

General Comments:

In this study, the authors reported a unified bilayer membranes (denoted CSO1M3) with a reinforced dual-crosslinking interface between barrier and porous layers, fabricated via a homogeneous isomeric in-situ strategy. The membrane's superior mechanical strength (~157 MPa in dry state and ~8.6 MPa in wet state) ensures its long-term degradation stability and bioactivity in vivo. Through systematic in vitro and in vivo experiments, the authors further evaluated stage-adaptive regulation of the unified bilayer membranes across the bone-repairing cascade: antibacterial (Stage I), immunomodulation (Stage II), neovascularization (Stage III) and osteogenesis (Stage IV). Bulk RNA-seq analyses were performed at Stage I and Stage IV to compare the CSO1M3 and control groups to identify differentially expressed genes, which were enriched in key biological functions and pathways. While these bulk transcriptomic insights are valuable, it is important to note that bulk RNA-seq captures only the average transcriptional signal from a heterogeneous tissue mixture. This approach inevitably masks the distinct contributions and dynamic responses of individual cell subpopulations—such as specific macrophage phenotypes, osteoprogenitor subsets, endothelial subtypes, and neural cells—that collectively drive the regenerative process. In addition, this study fails to capture the continuous, time-resolved transitions in cell states, and couldn't delineate the cellular trajectories that illustrate the progression of regeneration over time. The authors provided a clear schematic illustrating the in vivo experimental timeline from 1 to 8 week across the bone-repairing cascades, which offers an excellent framework for deeper cellular investigation. To address the existing gaps in cellular heterogeneity, it is recommended to employ single-cell sequencing methodologies in subsequent validation. Performing single-cell RNA sequencing on cells isolated from the implantation site at the 8 time points would allow for an unbiased decoding the complex cellular landscape.

Response:

We sincerely thank the reviewer for giving positive comments and insightful advice to help us improve the manuscript. We have supplemented the single-cell RNA sequencing (scRNA-seq) experiments and spatial analysis in revised manuscript according to the

reviewer's suggestion. We established a rat cranial critical-sized defect model and harvested newly formed tissues from the control and CSO_1M_3 groups. However, due to limited time for revise, we chose time points at 1 and 2 weeks post-implantation for scRNA-seq analysis. At first, the dot plots and Uniform Manifold Approximation and Projection (UMAP) were calculated to visualize cell heterogeneity as shown in Fig. R5 (Refer to Fig. 7a-d, Page 32 of Revision) and Fig. R6 (Refer to Supplementary Fig. 47, Page 27 of Supplementary Information). 9 distinct cell clusters were identified based on marker gene expression, including B cell (Pax5, Ms4a1), Neutrophil (Mmp8, Cd177), T cell (Cd3d, Cd3e), Monocyte (Csf1r, Cd14), Macrophage (Cd68, Mrc1), Osteo-lineage cell (Col1a1, Postn), Endothelial cell (Emcn, Sox7), Pericyte (Des, Mcam), and Schwann cell (Sox2, Sox10) (*Bone Research, 2024, 12:56, Science Advances, 2024, 10 (20): eadt6343*). These results provided a single-cell atlas of the regenerative bone tissues to reveal the cellular heterogeneity during repair. Notably, the CSO_1M_3 group exhibited a higher proportion of osteo-lineage cells than the control group during the first two weeks, with a sequential increase.

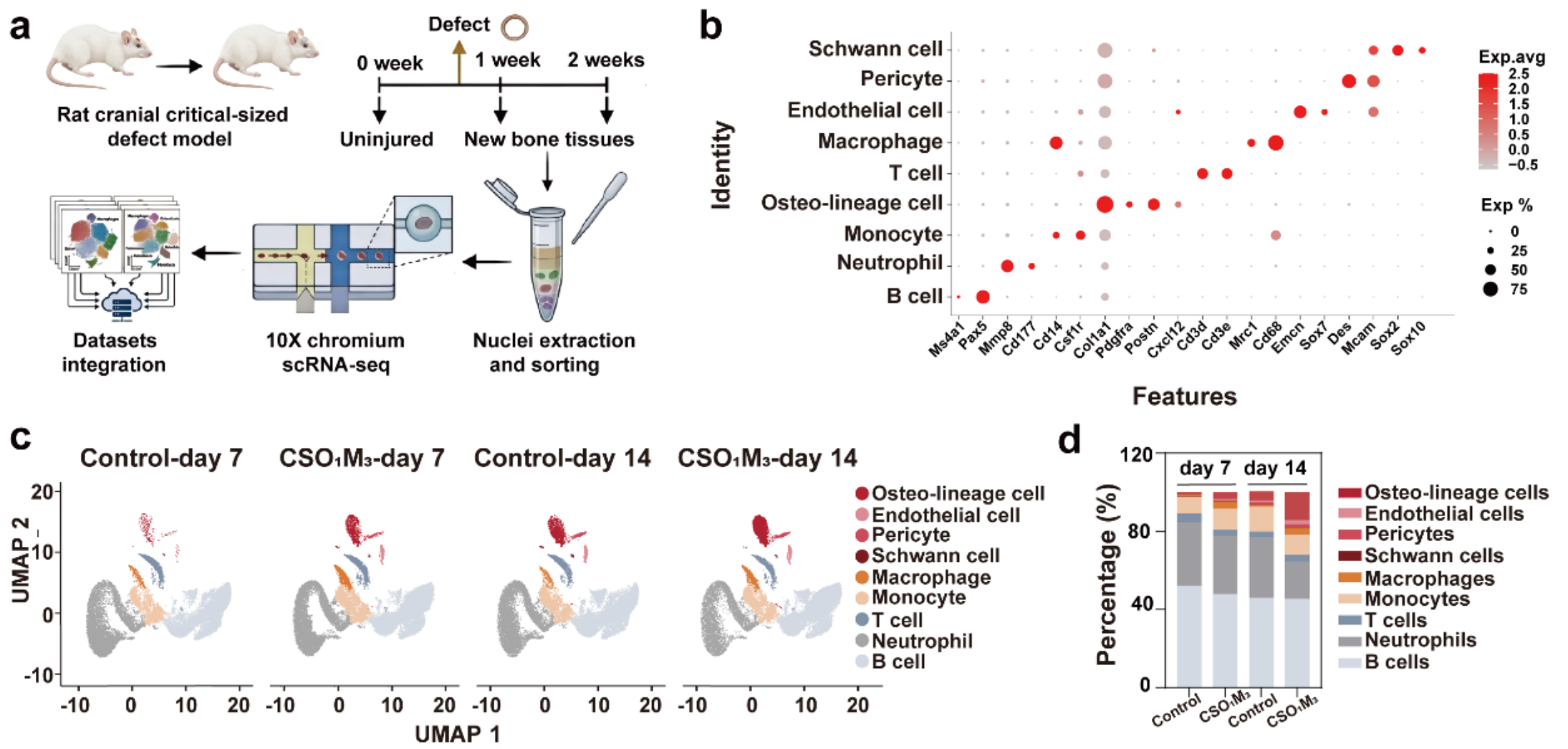

Fig. R5. scRNA-seq of the regenerative bone tissues from the control and CSO_1M_3 groups. a Flowchart of the single-cell transcriptome study on a rat cranial critical-sized defect model at different time points. **b** Dot plots of marker genes expression to define 9 distinct cell clusters, including B cell, Neutrophil, T cell, Monocyte, Macrophage, Osteo-lineage cell, Endothelial cell, Pericyte, and Schwann cell. Dot size represented the proportion of cells expressing a given gene in the cluster, and the color bar represented the gene expression levels. **c** UMAP visualization of the 9 cell clusters in the control and CSO_1M_3 groups after 1 and 2 weeks post-surgery, respectively. **d** Relative proportion of cell populations among the above four groups. Values represent the mean (*n* = 3 samples per group).

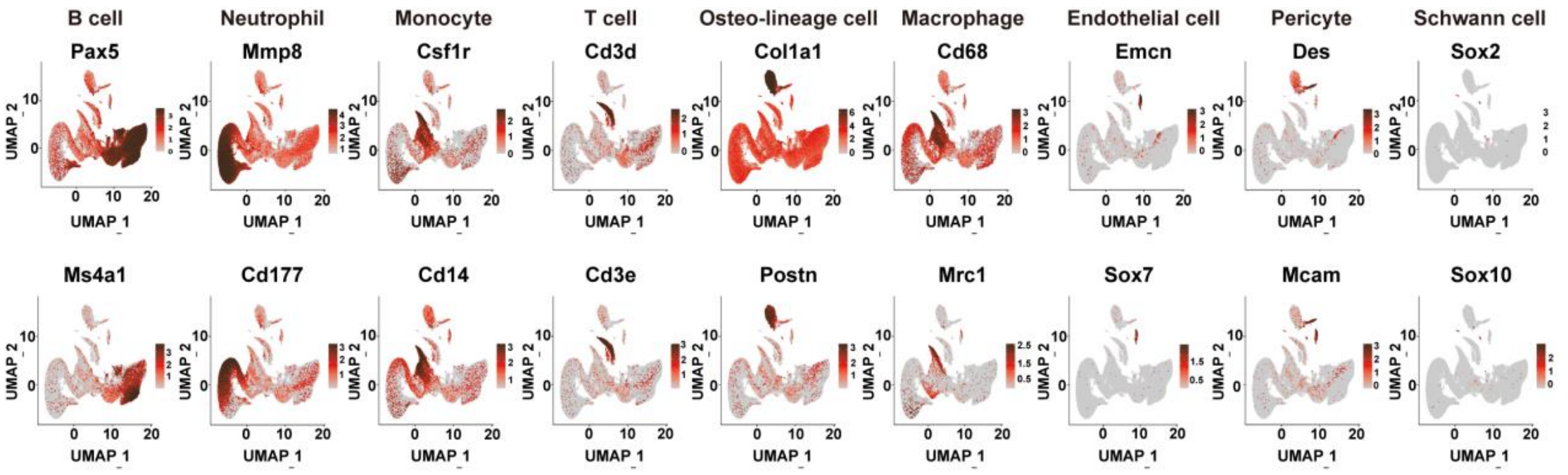

Fig. R6. UMAP visualization showing the expression of cell-specific marker genes used to identify 9 distinct cell clusters: B cell (Pax5, Ms4a1), Neutrophil (Mmp8, Cd177), T cell (Cd3d, Cd3e), Monocyte (Csf1r, Cd14), Macrophage (Cd68, Mrc1), Osteo-lineage cell (Col1a1, Postn), Endothelial cell (Emcn, Sox7), Pericyte (Des, Mcam), and Schwann cell (Sox2, Sox10).

The results and methods have been added and highlighted in the revised manuscript (Refer to Page 31 and 44 of Revision).

Revision:

[Page 31, Paragraph 2]

"To further explore the underlying mechanism of bone repair, the single-cell RNA sequencing (scRNA-seq) was performed on harvested newly formed tissues from the control and CSO_1M_3 groups (Fig. 7a). Dot plots and Uniform Manifold Approximation and Projection (UMAP) were calculated to visualize cell heterogeneity (Fig. 7b and Supplementary Fig. 47). 9 distinct cell clusters were identified based on marker gene expression, including B cell (Pax5, Ms4a1), Neutrophil (Mmp8, Cd177), T cell (Cd3d, Cd3e), Monocyte (Csf1r, Cd14), Macrophage (Cd68, Mrc1), Osteo-lineage cell (Col1a1, Postn), Endothelial cell (Emcn, Sox7), Pericyte (Des, Mcam), and Schwann cell (Sox2, Sox10) [59,60]. These results provided a single-cell atlas of the regenerative bone tissues to reveal the cellular heterogeneity during repair. Notably, the CSO_1M_3 group exhibited a higher proportion of osteo-lineage cells than the control group during the first two weeks, with a sequential increase (Fig. 7d)."

[Page 44, Paragraph 2]

"**Single-cell RNA sequencing experiments**

A SD rat cranial critical-sized defect model was established, and newly formed tissues from the control and CSO_1M_3 groups were harvested at 1 and 2 weeks post-surgery.

The surrounding tissues were meticulously removed to eliminate interference from non-target cell populations. The samples were mechanically dissociated into fragments and enzymatically digested in a mixture of 0.1% Type I collagenase (Gibco, 17100017) and 0.1% Type II collagenase (Gibco, 17101015) at 37°C for 40 min. The resulting cell suspension was filtered through a 70 μm nylon mesh and centrifuged. After removing erythrocytes using Red Blood Cell Lysis Solution (Miltenyi Biotec, 130-094-183), cell concentration and viability (> 85%) were determined using a Luna™ Automated Cell Counter (Logos Biosystems, Anyang, South Korea). The single-cell suspension was adjusted to 700 - 1200 cells μL^{-1}. Cell capture was performed according to the manufacturer's instructions for the 10× Genomics Chromium Next GEM Single Cell 3′ Reagent Kits v3.1 (USA, PN-1000268). The final libraries were sequenced on the BGI DNBSEQ-T7 PE100 (MGI Tech Co., Ltd., Shenzhen, China) in paired-end 150 bp mode.

The raw FASTQ files were processed using Cell Ranger (v9.0.0) for alignment against the rat reference genome (Rnor_6.0) and generation of the feature-barcode matrix. Quality control was conducted using the Seurat R package (v5.0); high-quality cells were retained based on the criteria of nFeature_RNA (200 - 5000) and mitochondrial gene percentage (< 10%). Principal Component Analysis (PCA) was applied for dimension reduction, and the Harmony algorithm was employed to integrate samples and remove batch effects. The first 30 principal components were visualized via t-SNE, and cell clustering was performed using the FindClusters function based on the SNN algorithm. For macrophage and osteoblast sub-clusters, coordinates were extracted for secondary dimension reduction and clustering. Sub-populations were identified based on differentially expressed genes (DEGs) and canonical markers. GO enrichment analysis was performed using clusterProfiler (v4.0) to investigate the dynamic evolution of biological processes (BP) across different time points and experimental groups. CytoTRACE (v0.3.3) was utilized to predict differentiation potential and the origin of the osteogenic lineage. Subsequently, Monocle 3 (v1.3.1) was used to construct pseudotime trajectories by learning the manifold structure, thereby revealing the dynamic differentiation landscape and lineage progression during the bone regeneration process. Finally, intercellular communication was inferred using CellChat (v1.6.1) based on the ligand-receptor interaction database."

Comments:

1. This high-resolution approach would enable the authors to: 1) identify the specific immune cell subsets (e.g., M2a, M2c macrophages) recruited or modulated by the CSO_1M_3; 2) delineate the differentiation trajectories of osteoblasts, chondrocytes and other cell lineages using trajectory inference tools such as monocle3 or PAGA; and 3) reconstruct the cell-cell communication networks that are altered by the implant, utilizing tools like CellChat or CellPhoneDB to predict key ligand-receptor interaction within the regenerating niche.

Response: We thank for the review's suggestion. The corresponding results and analysis were presented below.

1) To dissect cellular heterogeneity and classify the macrophages subsets, we merged macrophages obtained from the control group and CSO_1M_3 group at 1 week post-implantation for subclustering. Based on marker gene expression, 10 macrophage subclusters were identified, including Subcluster 1 (Cxcl3, Ly6c), Subcluster 2 (Mmp9, Cd177), Subcluster 3 (Gbp5, Astl), Subcluster 4 (Slc10a6, Stab1), Subcluster 5 (Mertk, Ltc4s), Subcluster 6 (Dnm1, Rarres1), Subcluster 7 (Col5a1, Mt3), Subcluster 8 (RT1-Doa, Cd7), Subcluster 9 (Pax5, Prkca), and Subcluster 10 (Ccna2, Plk1) (Fig. R7a-g and Fig. R8) (Refer to Fig. 7e-g, Page 32 of Revision and Supplementary Fig. 48, Page 28 of Supplementary Information). According to published reports, these subclusters could be categorized into four groups, including the Inflammatory macrophage group (Subcluster 1-3), M2c-like macrophage group (Subcluster 4-5), M2a-like macrophage group (Subcluster 6-7), and Others macrophage group (Subcluster 8-10). In particular, the M2c-like group was associated with clearing cellular debris and suppressing inflammation, and the M2a-like group contributed to tissue repair and remodeling through secreting regenerative factors (*Advanced Functional Materials, 2022, 32: 2208639; Nature communications, 2024, 15, 1666*). In addition, qualitative and quantitative analysis revealed that the CSO_1M_3 membranes markedly increase the population of the M2a-like group during early stage of bone repair.

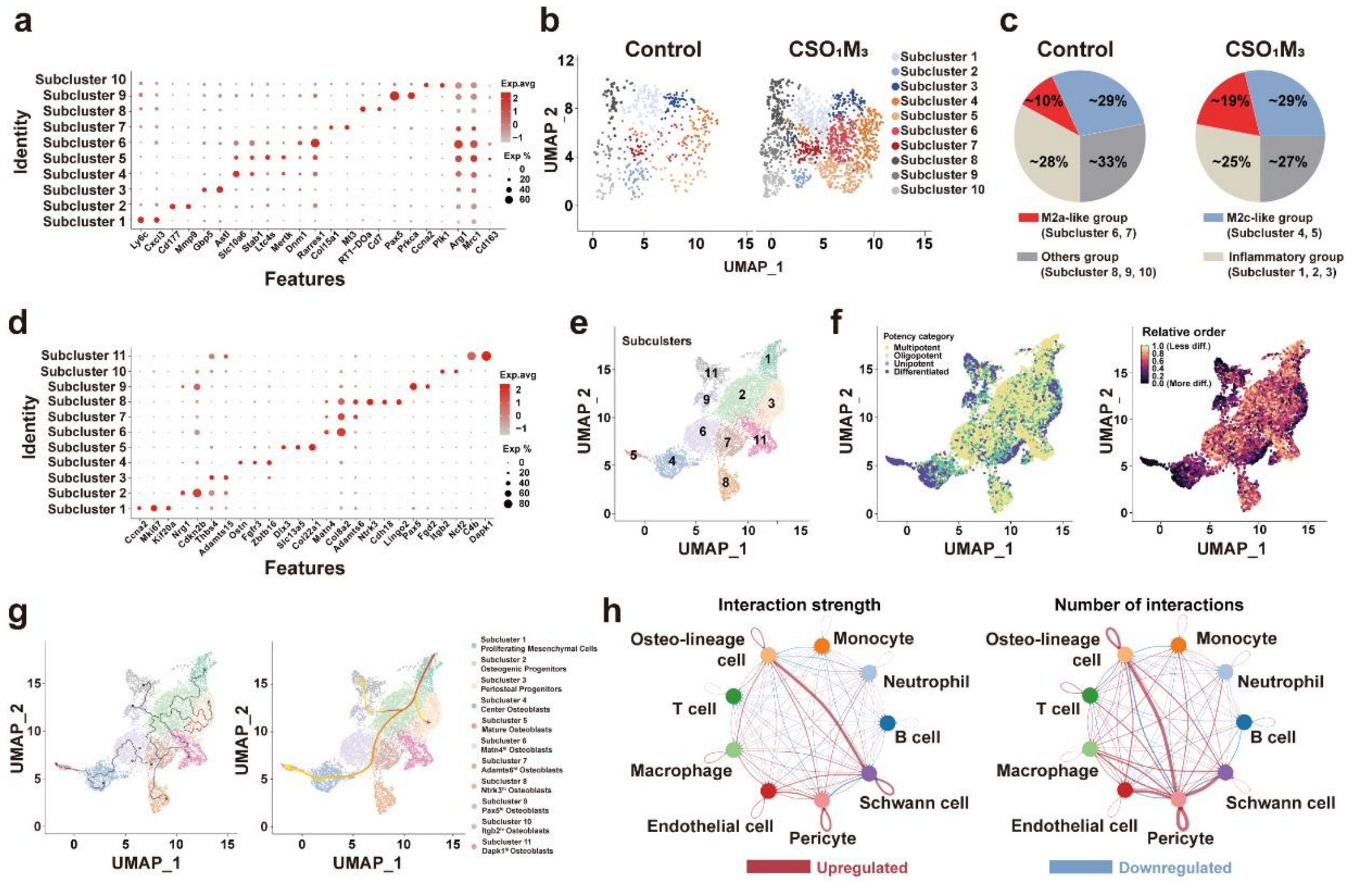

Fig. R7. scRNA-seq results analysis. a Dot plots of marker genes expression to define 10 cell subclusters of macrophages in the control and CSO_1M_3 groups after 1 week post-surgery. Dot size represented the proportion of cells expressing a given gene in the cluster, and the color bar represented the gene expression levels. **b** UMAP visualization of the 10 macrophage subclusters. **c** Quantitative analysis of functional groups of the macrophage subpopulations in the control and CSO_1M_3 groups. Values represent the mean ($n = 3$ samples per group). **d** Dot plots displaying the expression of representative marker genes for 11 cell subclusters of the OLCs in the control and CSO_1M_3 groups after 1 and 2 weeks post-surgery. **i** UMAP visualization of the 11 OLC subclusters. **e** CytoTRACE analysis illustrating the OLCs differentiation potential. Left: Cellular potency. Right: Relative differentiation order. **f** Developmental trajectory of the OLCs inferred by Monocle 3. Left: Pseudotime trajectory colored by subclusters. Right: Schematic of the predictable trajectory. **g** Cell-cell communication analysis between the control and CSO_1M_3 group at 1 week post implantation. Red lines: upregulated interactions. Blue lines: downregulated interactions. Cell clusters: B cell, Neutrophil, T cell, Monocyte, Macrophage, Osteo-lineage cell, Endothelial cell, Pericyte, and Schwann cell.

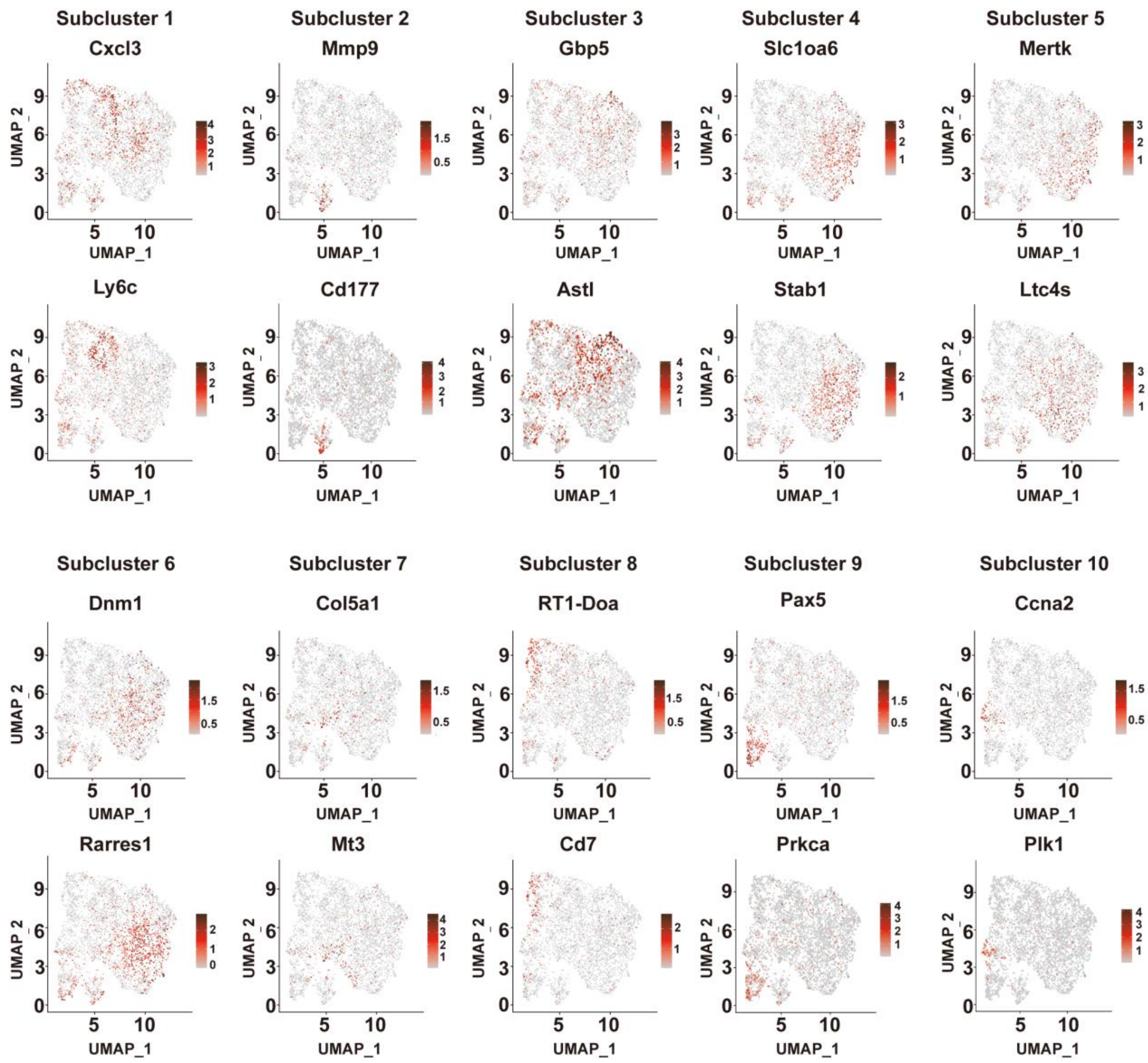

Fig. R8. UMAP visualization showing the expression of cell-specific marker genes used to identify 10 macrophage subclusters: Subcluster 1 (Cxcl3, Ly6c), Subcluster 2 (Mmp9, Cd177), Subcluster 3 (Gbp5, Astl), Subcluster 4 (Slc10a6, Stab1), Subcluster 5 (Mertk, Ltc4s), Subcluster 6 (Dnm1, Rarres1), Subcluster 7 (Col5a1, Mt3), Subcluster 8 (RT1-Doa, Cd7), Subcluster 9 (Pax5, Prkca), and Subcluster 10 (Ccna2, Plk1).

2) The osteo-lineage cells (OLCs) in all the groups at 1 and 2 weeks post-implantation were also merged to analyze the cell heterogeneity. We identified and visualized the OLCs subpopulations through dot plots and UMAP analysis based on the clustering-specific markers (Fig. R7d-g and Fig. R8) (Refer to Fig. 7h-k, Page 32 of Revision and Supplementary Fig. 49, Page 29 of Supplementary Information). As a result, the OLC subpopulations were annotated as follows: Subcluster 1 represented the Proliferating Mesenchymal Cells (expressing Ccna2, Kif2c); Subcluster 2 represented the Osteogenic Progenitors (expressing Cdkn2b, Nrg1); Subcluster 3 represented the Periosteal Progenitors (expressing Thbs4, Adamts15); Subcluster 4 represented Center Osteoblasts (expressing Ostn, Fgfr3); Subcluster 5 represented Mature Osteoblasts (expressing Slc13a5, Dlx3); Subcluster 6 represented Matn4^{hi} Osteoblasts (expressing

Matn4, Ucma); Subcluster 7 represented Adamts6^{hi} Osteoblasts (expressing Adamts6, Col8a2); Subcluster 8 represented Ntrk3^{hi} Osteoblasts (expressing Ntrk3, Lingo2); Subcluster 9 represented Pax5^{hi} Osteoblasts (expressing Pax5, Fgd2); Subcluster 10 represented Itgb2^{hi} Osteoblasts (expressing Itgb2, Ncf2); and Subcluster 11 represented Dapk1^{hi} Osteoblasts (expressing Dapk1, C4b) (*Nature Communications, 2025, 16: 6200*). Furthermore, we performed CytoTRACE analysis to evaluate the cell differentiation potential, which identified the Proliferating Mesenchymal Cells (Subcluster 1) as the population with the highest stemness. Consequently, we defined Subcluster 1 as the root for the subsequent pseudotime analysis. Afterwards, Monocle 3 was employed to construct the single-cell trajectories, forming a clear, continuous developmental progression. The trajectory originated from the high-stemness proliferating pool (Subcluster 1) and first toward the functional state of the Osteogenic Progenitors (Subcluster 2). Then, trajectory bifurcated successively to the Periosteal Progenitors (Subcluster 3) and the osteoblasts (Subcluster 9-10). Notably, a major lineage commitment was identified moving through the Osteogenic Progenitors (Subcluster 2) and ultimately advancing toward osteoblasts (Subcluster 7-4-5). This trajectory illustrated the transition of the OLCs from an undifferentiated and proliferative state to a functionally specialized mineralizing state during the bone repair process.

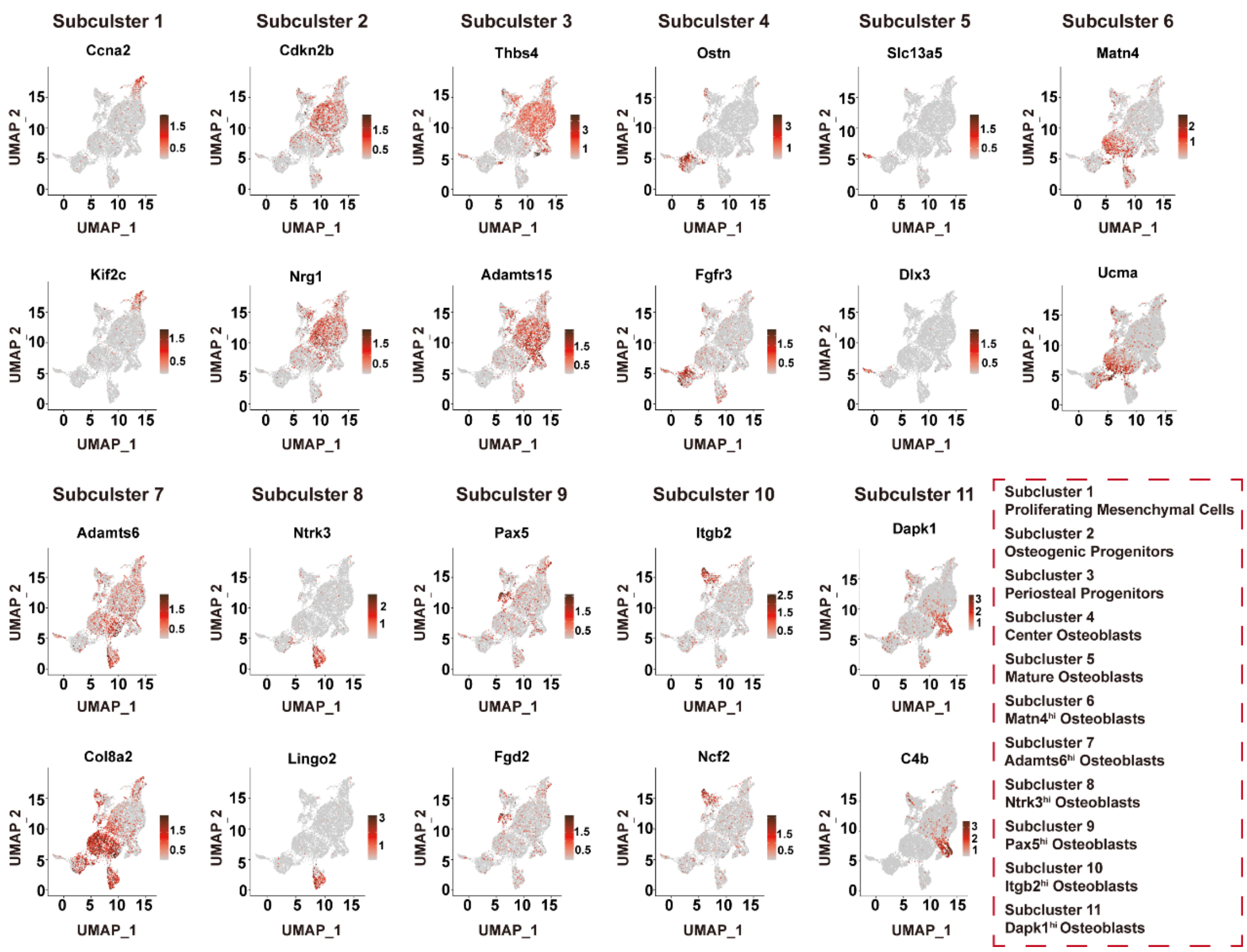

Fig. R9. UMAP visualization showing the expression of cell-specific marker genes used to identify Osteo-lineage cells (OLCs) subclusters: Subcluster 1 (Ccna2, Kif2c), Subcluster 2 (Cdkn2b, Nrg1), Subcluster 3 (Thbs4, Admts15), Subcluster 4 (Ostn, Fgfr3), Subcluster 5 (Slc13a5, Dlx3), Subcluster 6 (Matn4, Ucma), Subcluster 7 (Adamts6, Col8a2), Subcluster 8 (Ntrk3, Lingo2), Subcluster 9 (Pax5, Fgd2), Subcluster 10 (Itgb2, Ncf2), and Subcluster 11 (Dapk1, C4b).

3) As suggested, comprehensive CellChat analysis was also performed to evaluate cellular crosstalk among various cell clusters (Fig. R7h) (Refer to Fig. 7l, Page 32 of Revision). The strength and number of interactions were indicated by the degree of line thickness. Comparison between the control and CSO_1M_3 groups at 1 week post-implantation revealed a pronounced remodeling of the cellular communication network. Specifically, the CSO_1M_3 group exhibited enhanced interaction strengths and numbers among key regenerative cell clusters (such as the Schwann cells, endothelial cells and OLCs) relative to the control group. Within this optimized regenerative niche, Schwann cells and endothelial cells exhibited a robust, synchronized crosstalk with the OLCs. These results collectively demonstrated that the CSO_1M_3 membrane could promote a tightly integrated neurovascular-osteogenic communication network, thereby effectively improving bone regeneration.

The figures and results have been added and highlighted in the revised manuscript (Refer to Page 31-34 of Revision).

Revision:

[Page 31, Paragraph 3]

"To dissect cellular heterogeneity and classify the macrophages subsets, we merged macrophages obtained from the control group and CSO_1M_3 group at 1 week post-implantation for subclustering. Based on marker gene expression, 10 macrophage subclusters were identified, including Subcluster 1 (Cxcl3, Ly6c), Subcluster 2 (Mmp9, Cd177), Subcluster 3 (Gbp5, Astl), Subcluster 4 (Slc10a6, Stab1), Subcluster 5 (Mertk, Ltc4s), Subcluster 6 (Dnm1, Rarres1), Subcluster 7 (Col5a1, Mt3), Subcluster 8 (RT1-Doa, Cd7), Subcluster 9 (Pax5, Prkca), and Subcluster 10 (Ccna2, Plk1) (Fig. 7e and f, Supplementary Fig. 48). According to published reports, these subclusters could be categorized into four groups, including Inflammatory macrophage group (Subcluster 1-3), M2c-like macrophage group (Subcluster 4-5), M2a-like macrophage group (Subcluster 6-7), and Others macrophage group (Subcluster 8-10). In particular, the M2c-like group was associated with clearing cellular debris and suppressing inflammation, and the M2a-like group contributed to tissue repair and remodeling through secreting regenerative factors [61,62]. In addition, qualitative and quantitative analysis revealed that the CSO_1M_3 membranes markedly increase the population of the M2a-like group during early stage of bone repair (Fig. 7g).

The osteo-lineage cells (OLCs) in all the groups at 1 and 2 weeks post-implantation were also merged to analyze the cell heterogeneity. We identified and visualized the OLCs subpopulations through dot plots and UMAP analysis based on the clustering-specific markers (Fig. 7h and Supplementary Fig. 49). As a result, the OLC subpopulations were annotated as follows: Subcluster 1 represented the Proliferating Mesenchymal Cells (expressing Ccna2, Kif2c); Subcluster 2 represented the Osteogenic Progenitors (expressing Cdkn2b, Nrg1); Subcluster 3 represented the Periosteal Progenitors (expressing Thbs4, Adamts15); Subcluster 4 represented Center Osteoblasts (expressing Ostn, Fgfr3); Subcluster 5 represented Mature Osteoblasts (expressing Slc13a5, Dlx3); Subcluster 6 represented $Matn4^{hi}$ Osteoblasts (expressing Matn4, Ucma); Subcluster 7 represented $Adamts6^{hi}$ Osteoblasts (expressing Adamts6,

Col8a2); Subcluster 8 represented Ntrk3^{hi} Osteoblasts (expressing Ntrk3, Lingo2); Subcluster 9 represented Pax5^{hi} Osteoblasts (expressing Pax5, Fgd2); Subcluster 10 represented Itgb2^{hi} Osteoblasts (expressing Itgb2, Ncf2); and Subcluster 11 represented Dapk1^{hi} Osteoblasts (expressing Dapk1, C4b) (Fig. 7i) [63]. Furthermore, we performed CytoTRACE analysis to evaluate the cell differentiation potential, which identified the Proliferating Mesenchymal Cells (Subcluster 1) as the population with the highest stemness (Fig. 7j). Consequently, we defined Subcluster 1 as the root for the subsequent pseudotime analysis. Afterwards, Monocle 3 was employed to construct the single-cell trajectories, forming a clear, continuous developmental progression (Fig. 7k). The trajectory originated from the high-stemness proliferating pool (Subcluster 1) and first toward the functional state of the Osteogenic Progenitors (Subcluster 2). Then, trajectory bifurcated successively to the Periosteal Progenitors (Subcluster 3) and the osteoblasts (Subcluster 9-10). Notably, a major lineage commitment was identified moving through the Osteogenic Progenitors (Subcluster 2) and ultimately advancing toward osteoblasts (Subcluster 7-4-5). This trajectory illustrated the transition of the OLCs from an undifferentiated and proliferative state to a functionally specialized mineralizing state during the bone repair process.

Comprehensive CellChat analysis was also performed to evaluate cellular crosstalk among various cell clusters (Fig. 7l). The strength and number of interactions were indicated by the degree of line thickness. Comparison between the control and CSO_1M_3 groups at 1 week post-implantation revealed a pronounced remodeling of the cellular communication network. Specifically, the CSO_1M_3 group exhibited enhanced interaction strengths and numbers among key regenerative cell clusters (such as the Schwann cells, endothelial cells and OLCs) relative to the control group. Within this optimized regenerative niche, Schwann cells and endothelial cells exhibited a robust, synchronized crosstalk with the OLCs. These results collectively demonstrated that the CSO_1M_3 membrane could promote a tightly integrated neurovascular-osteogenic communication network, thereby effectively improving bone regeneration."

2. In addition, spatial transcriptomics (e.g., Visium) is advised to clarify the three-dimensional spatial relationships between nerves, vessels, and new bone, enabling direct analysis of co-localization patterns.

Response:

We sincerely appreciate the reviewer's valuable suggestion. We fully acknowledge that spatial transcriptomics is a useful tool for studying the spatial relationships of new-growth tissues. However, due to its technically demanding and time-consuming sample processing requirements for mineralized bone tissue, we instead used a triple-color immunofluorescence staining approach to analyzing the spatial relationships among the new-growth nerves, blood vessels, and bone tissues. As shown in Fig. R10 (Refer to Supplementary Fig. 45, Page 26 of Supplementary Information), the $TUBB3^{+}$ nerves and $CD31^{+}$ blood vessels were not randomly distributed, but closely surrounded $Osterix^{+}$ newly formed bone tissues in all groups at 4 weeks post-implantation This specific spatial arrangement indicated these three systems might have a mutually reinforcing relationship. In particular, the CSO_1M_3 membrane induced significantly improved new-tissue formation and coupling intensity in the surrounding region. Generally, bones were covered by neural and vascular networks essential for the development, remodeling, and repair (*Military Medical Research, 2025, 12(1): 9*). Specifically, nerve regeneration regulated osteogenic differentiation and bone metabolism via neuropeptides and cytokines (e.g., CGRP, NGF). The neural pathways could also guide the spatial alignment of newly formed bone tissues. Angiogenesis established a nutrient supply network to supply the basic necessities (e.g. oxygen, nutrients, growth factors) and remove the waste products for the defect region. Blood vessels also played a key role in the recruitment of osteoprogenitor cells. Additionally, nerves and blood vessels interacted synergistically, which nerves promoted angiogenesis via neurotransmitter secretion, and blood vessels in turn supported nerve fiber growth by supplying oxygen and nutrients. Upregulated GO terms including nervous system development, epithelial differentiation, and osteoblast differentiation in Fig. R11 and R12 (Refer to Fig. 6j, Page 25 of Revision and Supplementary Fig. 46, Page 27 of Supplementary Information) also confirmed that the CSO_1M_3 group significantly enhanced the synergistic interaction among these new tissues compared to the control group. The CSO_1M_3 membrane was proved to establish a well-integrated spatial structure, highlighting its superior bioactivity for regulating stage-adaptive bone regeneration.

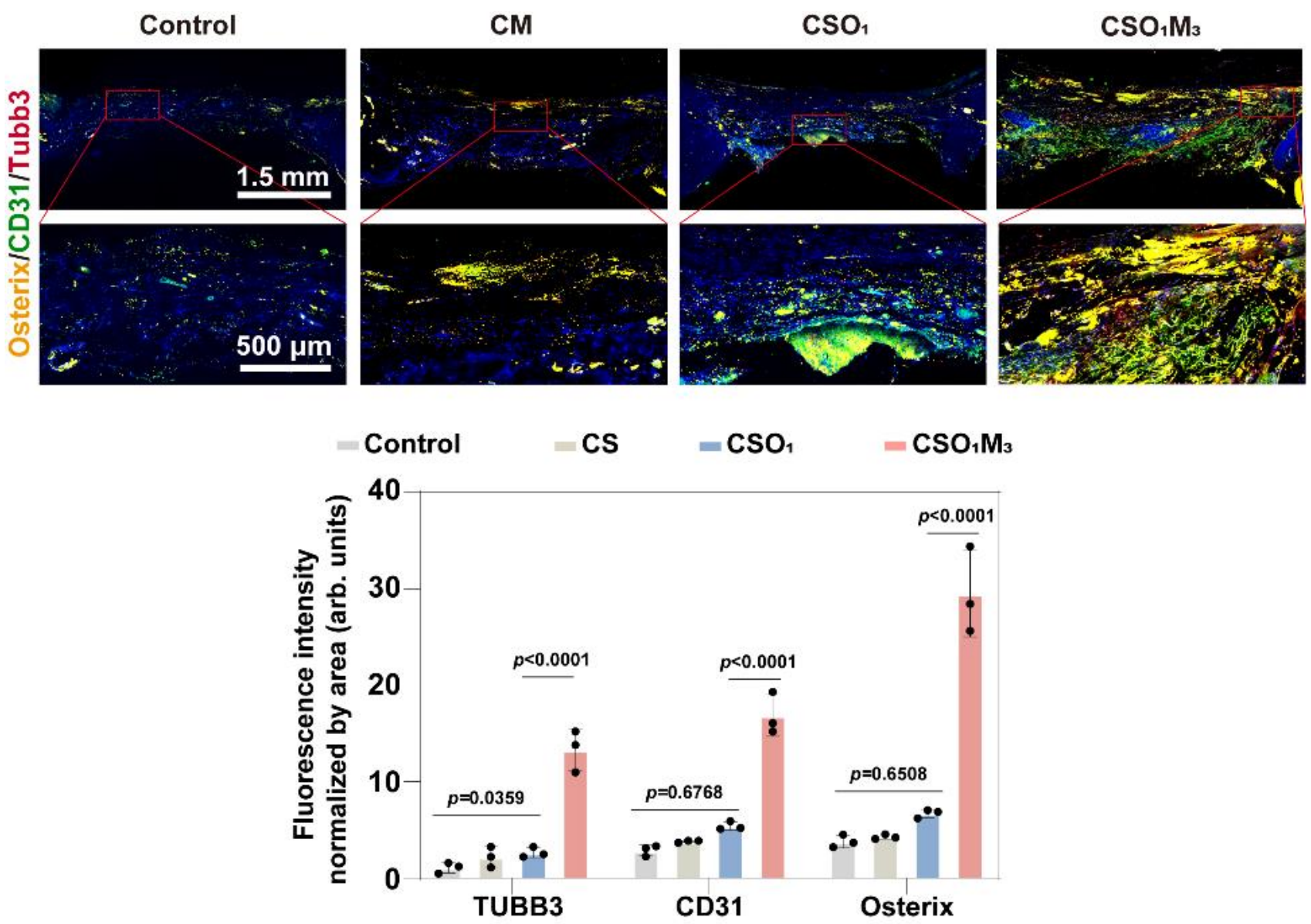

Fig. R10. Representative IF staining of TUBB3 (red, neural), CD31 (green, vascular) and Osterix (orange, osteogenic) images of the tissues from defective sites at 4 weeks post implantation and corresponding quantitative fluorescence intensities. Values represent the mean and standard deviation ($n = 3$ samples per group). Statistical analyses were performed using one-way ANOVA followed by Tukey's post hoc test. $p < 0.05$ was considered statistically significant differences between the compared groups.

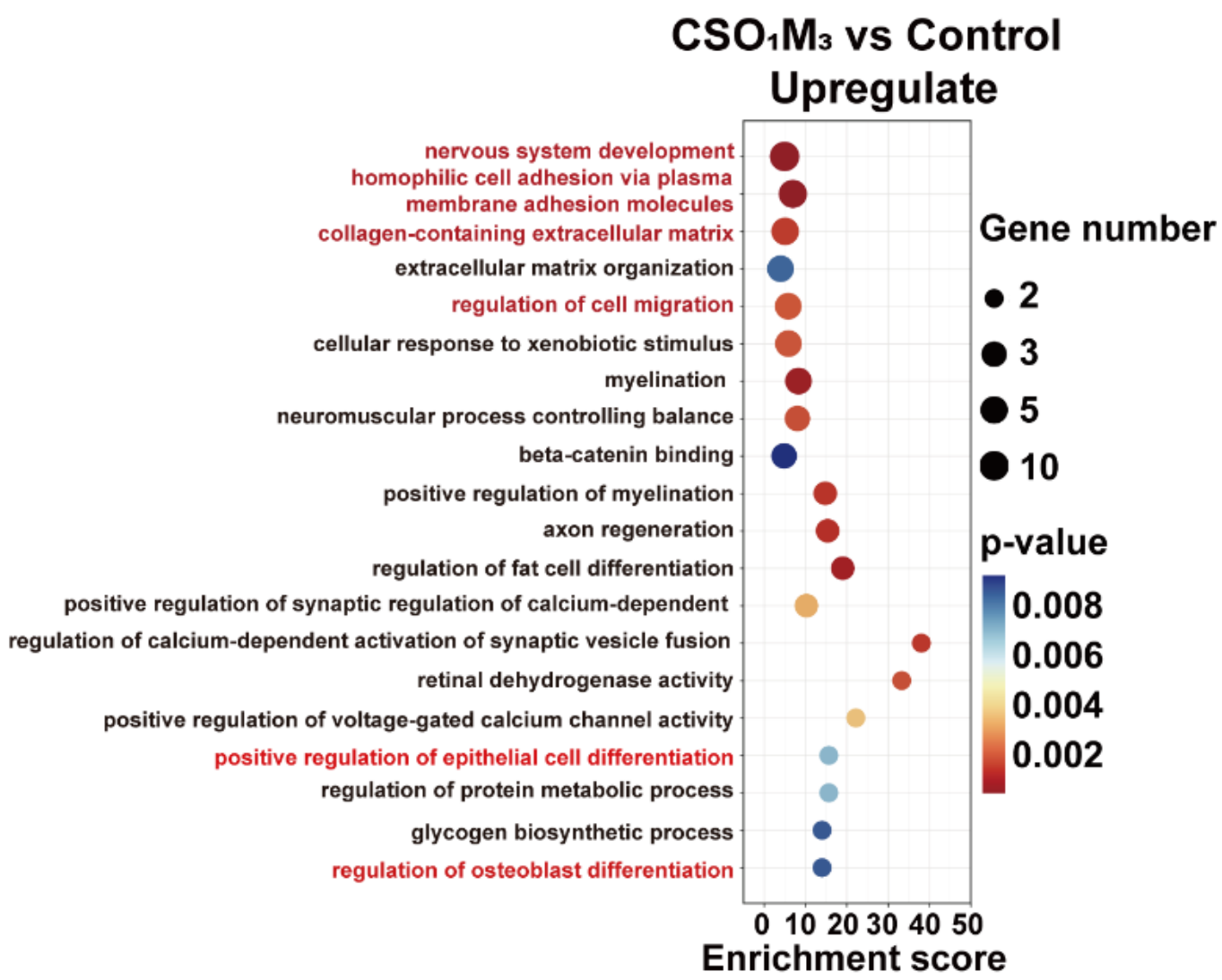

Fig. R11. Transcriptomic comparison between the CSO_1M_3 and control groups at 4 weeks post implantation. GO enrichment analysis of upregulated genes related to various biological responses and functions.

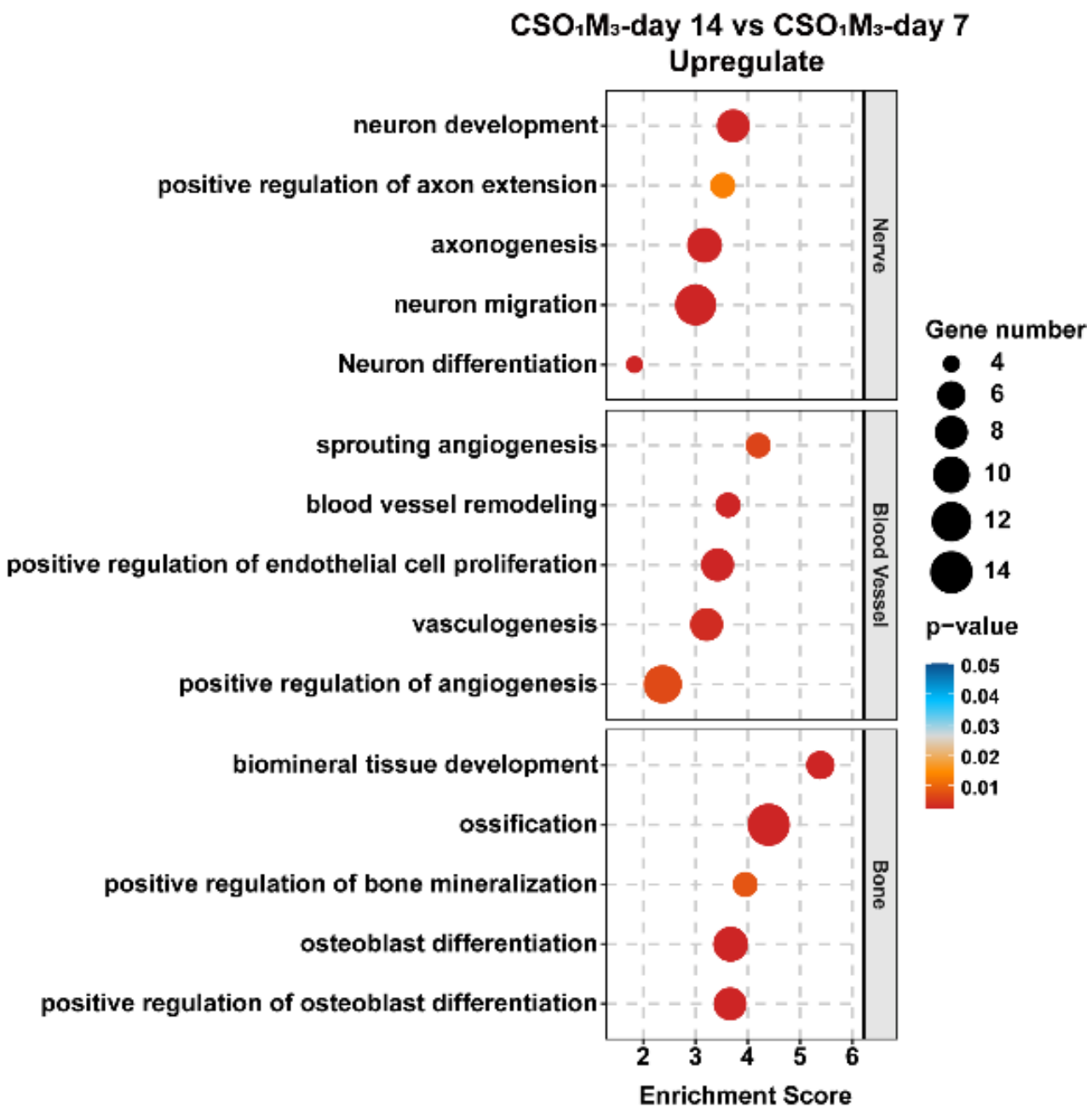

Fig. R12. Single-cell transcriptomic comparison between the CSO_1M_3 groups at 1 week and 2 weeks post implantation. GO enrichment analysis of upregulated genes related to nerve, blood vessel and bone tissues regeneration.

The results and corresponding discussion have also been supplemented in our revised manuscript (Refer to Page 30 of Revision).

Revision:

[Page 30, Paragraph 1 and 2]

"…In addition, a triple-color immunofluorescence staining results suggested the $TUBB3^+$ nerves and $CD31^+$ blood vessels were not randomly distributed, but closely surrounded $Osterix^+$ newly formed bone tissues in all groups at 4 weeks post-implantation (Supplementary Fig. 45). This specific spatial arrangement indicated these three systems might have a mutually reinforcing relationship. In particular, the CSO_1M_3 membrane induced significantly improved new-tissue formation and coupling intensity in the surrounding region…

Generally, bones were covered by neural and vascular networks essential for the development, remodeling, and repair [58]. Specifically, nerve regeneration regulated osteogenic differentiation and bone metabolism via neuropeptides and cytokines (e.g., CGRP, NGF). The neural pathways could also guide the spatial alignment of newly formed bone tissues. Angiogenesis established a nutrient supply network to supply the

basic necessities (e.g. oxygen, nutrients, growth factors) and remove the waste products for the defect region. Blood vessels also played a key role in the recruitment of osteoprogenitor cells. Additionally, nerves and blood vessels interacted synergistically, which nerves promoted angiogenesis via neurotransmitter secretion, and blood vessels in turn supported nerve fiber growth by supplying oxygen and nutrients. Upregulated GO terms including nervous system development, epithelial differentiation, and osteoblast differentiation in Fig. 6j and Supplementary Fig. 46 also confirmed that the CSO_1M_3 group significantly enhanced the synergistic interaction among these new tissues compared to the control group. The CSO_1M_3 membrane was proved to establish a well-integrated spatial structure, highlighting its superior bioactivity for regulating stage-adaptive bone regeneration."

Reviewer #3

General Comments:

In this study, the authors propose a novel periosteum-inspired composite membrane comprised of chitosan (CS), oxidized tannic acid (OTA) and magnesium oxide (MgO) nanoparticles, to mechanically protect and adaptively regulate the bone regeneration process. As natural periosteum's structure, the composite membrane was constructed with unified barrier layer and porous layer through a homogeneous isomeric in-situ fabrication strategy. This unified bilayer membrane was demonstrated to sequentially kill bacteria, regulate immune responses, improve neurovascular ingrowth and enhance osteogenesis through in vitro and in vivo studies. The authors also explored and discussed the regulatory mechanisms underlying stage-adaptive bone regeneration of the composite membrane. The manuscript is generally well-written, logically structured, and supported by extensive data. Therefore, the manuscript is recommended for publication after moderate revision. Some comments and suggestions are listed as below:

Response:

We appreciate the reviewer for giving positive comments and insightful advices to help us improve the manuscript.

1. Bioactive implants usually work in a physiologically wet environment, so it is impressive that the proposed membrane has a good mechanical strength (~8.6 MPa) in wet state. The reader wonders what the strength of the membrane will be after biodegradation for 8 weeks (the period for bone regeneration in this work). And whether the strength is sufficient to maintain suturing with the tissues or not.

Response:

Thanks so much for this reviewer's valuable comment. The qualitative and quantitative analysis of the structural stability of the composite membrane after degradation for 8 weeks have been supplemented as shown in Fig. R13 (Refer to Supplementary Fig. 7, Page 6 of Supplementary Information). The description has also been added and highlighted in our revised manuscript (Refer to Page 9 of Revision).

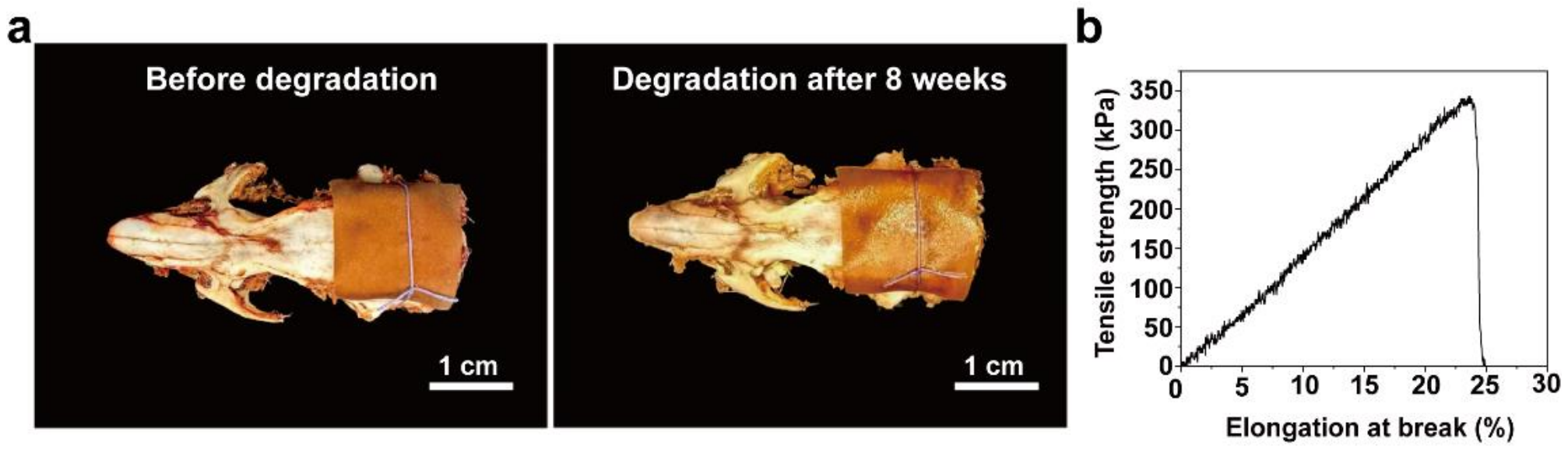

Fig. R13. The qualitative (a) and quantitative (b) analysis of the structural stability of the CSO_1M_3 membrane after degradation for 8 weeks. $n = 3$ samples per group.

Revision:

[Page 9, Paragraph 2]

"Macroscopic observations confirmed the shape stability of the CSO_1M_3 membranes on the rat skull after 8 weeks of degradation, with a corresponding residual tensile strength of ~300 kPa (Supplementary Fig. 7). This mechanical performance could ensure stable tissue suturing for structural support during bone repair process."

2. Since the dual-crosslinking network is important for the composite membrane, it would be helpful to provide a schematic figure to describe its preparation process

Response:

We thank the reviewer's kind suggestion. As shown in Fig. R14 (Refer to Supplementary Fig. 1, Page 3 of Supplementary Information), the composite membrane was fabricated using chitosan (CS), oxidized tannic acid (OTA) and magnesium oxide (MgO) nanoparticles. The structural backbone of the composite membranes was constructed via covalent Schiff-base linkages between the amino groups of CS and the quinone groups of oxidized OTA. This primary network was further strengthened through phenolic coordination systems formed by the phenolic hydroxyl groups of OTA and MgO nanoparticles. To achieve high mechanical properties and mimic the bilayer architecture of native periosteum, we utilized a homogeneous isomeric in-situ forming method to unify the two disparate layers of the composite membranes. This fabrication strategy brings physical stacking with chemical crosslinking interaction at the interface between the two layers of the composite membranes, which can eliminate interfacial discontinuities between layers to enhance structural uniformity and mechanical reliability of the membranes for supporting the entire bone repair process.

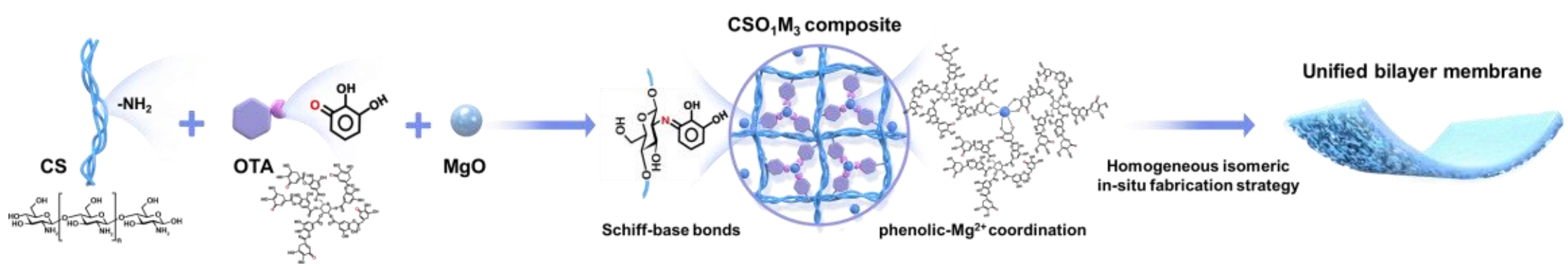

Fig. R14. Schematic illustration of preparation of the unified bilayer membranes.

3. The authors propose triple antibacterial mechanisms by the unified composite membrane. Please add a brief discussion on the relative contributions of these mechanisms, highlighting which might be the most critical.

Response:

We thank for reviewer's comment. In this study, we summarized triple antibacterial mechanisms of the composite membrane including the Rapid Chemical Elimination of Planktonic Bacteria (RCEPB) mechanism, the Dynamic Anti-Adhesion and Contact-Killing mechanism (DAACK), and the Physical Exclusion by Dense Barrier Layer (PEDBA) mechanism in Fig. R15 (Refer to Fig. 3j, Page 12 of Revision). Among these mechanisms, the RCEPB mechanism might be the dominant one because it provided rapid and effective antibacterial defense at the initial stage of bacterial contamination. The DAACK mechanism then eliminated bacteria that reached the membrane's surface, preventing the formation of mature biofilms through the smooth surface and positive charges of the CS matrix. Furthermore, the PEDBA mechanism could provide long-term barrier function to block bacterial penetration into the defective site. The results in Fig. R16 (Refer to Supplementary Fig. 10, Page 7 of Supplementary Information) showed that the CSO_1M_3 membranes were demonstrated with significantly higher 24-hour antibacterial rates against Staphylococcus aureus (*S. aureus*) (~74%) and Escherichia coli (*E. coli*) (~70%) compared to those of the CS membranes (~46% and ~40%), representing a ~1.6-fold improvement. Therefore, these synergistic effects endowed the CSO_1M_3 membrane with comprehensive antibacterial protection, creating a favorable microenvironment for efficient bone regeneration.

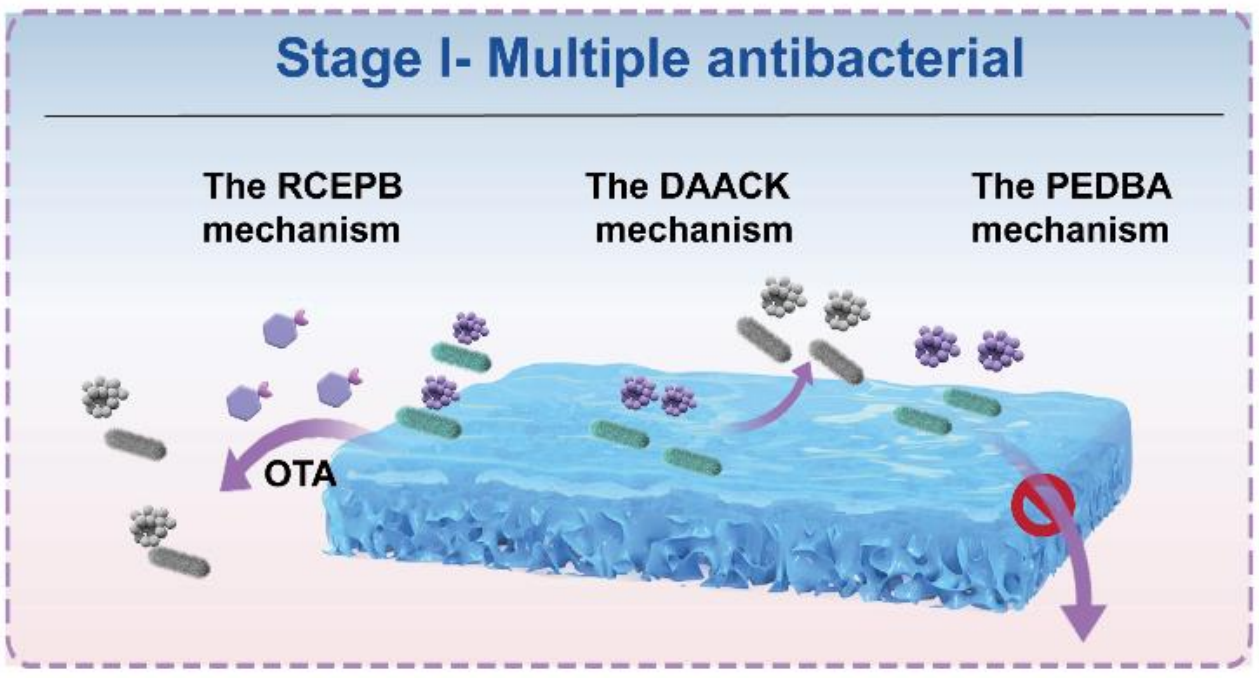

Fig. R15. Schematic of antibacterial mechanisms of the unified bilayer membranes. The RCEPB mechanism: Rapid Chemical Elimination of Planktonic Bacteria mechanism. The DAACK mechanism: Dynamic Anti-Adhesion and Contact-Killing mechanism. The PEDBA mechanism: Physical Exclusion by Dense Barrier Layer mechanism.

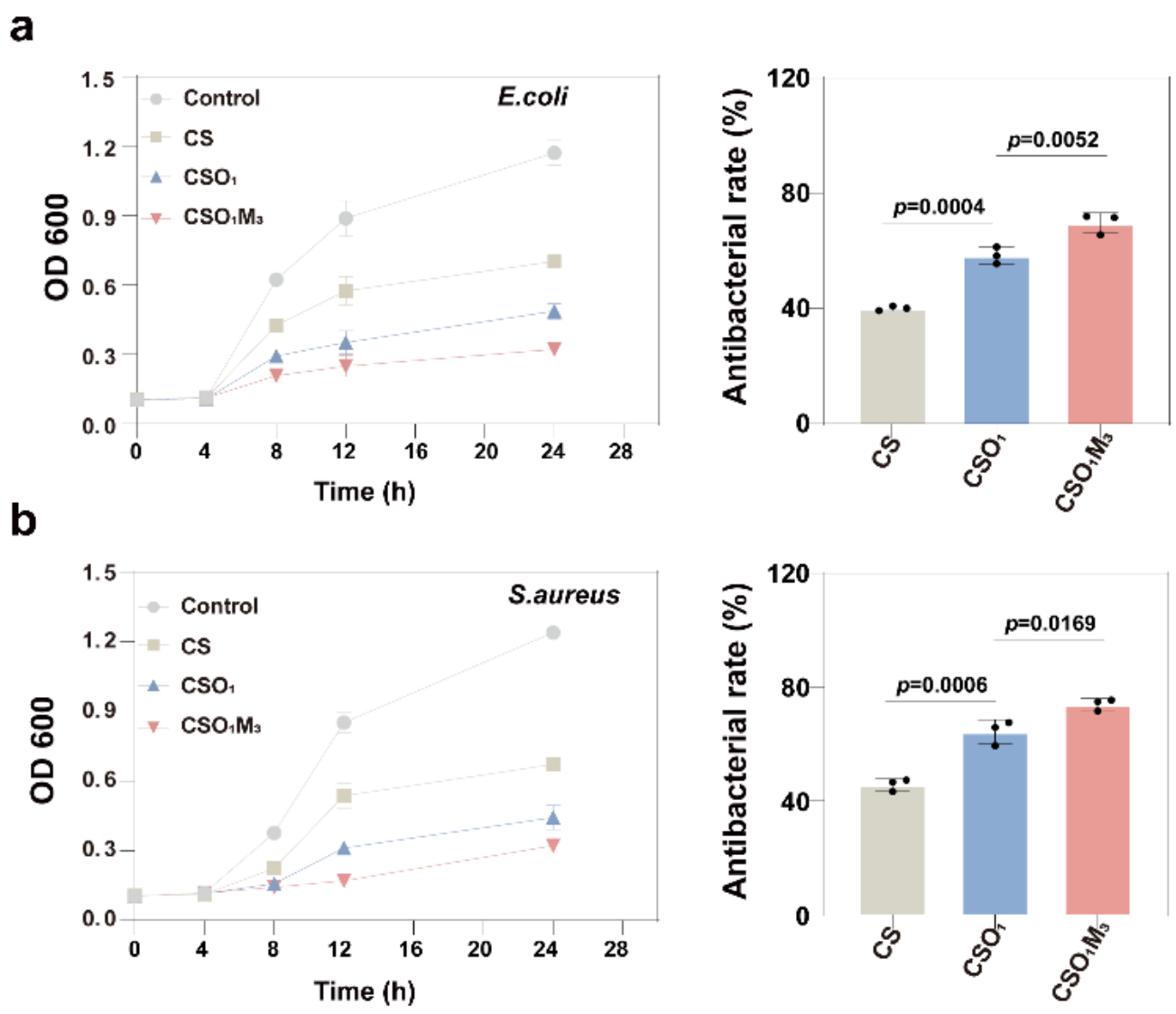

Fig. R16. Antibacterial properties of the CS, CSO_1, and CSO_1M_3 membranes. a Bacterial growth curves and antimicrobial rate against Escherichia coli (*E. coli*). **b** Bacterial growth curves and antimicrobial rate against Staphylococcus aureus (*S. aureus*). Values in (a) and (b) represent the mean and standard deviation ($n = 3$ samples per group). Statistical analyses were performed using one-way ANOVA followed by Tukey's post hoc test. $p < 0.05$ was considered statistically significant differences between the compared groups.

The discussion has also been added and highlighted in our revised manuscript (Refer to Page 14 of Revision).

Revision:

[Page 14, Paragraph 1]

"Among these mechanisms, the RCEPB mechanism might be the dominant one because

it provided rapid and effective antibacterial defense at the initial stage of bacterial contamination. The DAACK mechanism then eliminated bacteria that reached the membrane's surface, preventing the formation of mature biofilms through the smooth surface and positive charges of the CS matrix. Furthermore, the PEDBA mechanism could provide long-term barrier function to block bacterial penetration into the defective site."

4. Neurovascularization (Stage III) at the bone defect site seems to serve as a linking stage during the bone regeneration process. Please explain the effects of both nerve regeneration and angiogenesis on the ultimate bone repair.

Response:

We appreciate the reviewer's comment. Previous reports have shown that bones are covered by neural and vascular networks essential for the development, remodeling, and repair (*Military Medical Research, 2025, 12(1): 9*). Specifically, nerve regeneration regulated osteogenic differentiation and bone metabolism via neuropeptides and cytokines (e.g., CGRP, NGF). The neural pathways could also guide the spatial alignment of newly formed bone tissues. Angiogenesis established a nutrient supply network to supply the basic necessities (e.g. oxygen, nutrients, growth factors) and remove the waste products for the defect region. Blood vessels also played a key role in the recruitment of osteoprogenitor cells. Additionally, nerves and blood vessels interacted synergistically, which nerves promoted angiogenesis via neurotransmitter secretion, and blood vessels in turn supported nerve fiber growth by supplying oxygen and nutrients. In this work, the CSO_1M_3 membrane induced significantly improved new-tissue formation and coupling intensity in the surrounding region. Upregulated GO terms including nervous system development, epithelial differentiation, and osteoblast differentiation in Fig. R17 and Fig. R18 (Refer to Fig. 6j, Page 25 of Revision and Supplementary Fig. 46, Page 27 of Supplementary Information) also confirmed that the CSO_1M_3 group significantly enhanced the synergistic interaction among these new tissues compared to the control group. The CSO_1M_3 membrane was proved to establish a well-integrated spatial structure, highlighting its superior bioactivity for regulating stage-adaptive bone regeneration. Consequently, the neurovascularization effectively stimulated osteogenesis and ultimately promoted bone repair.

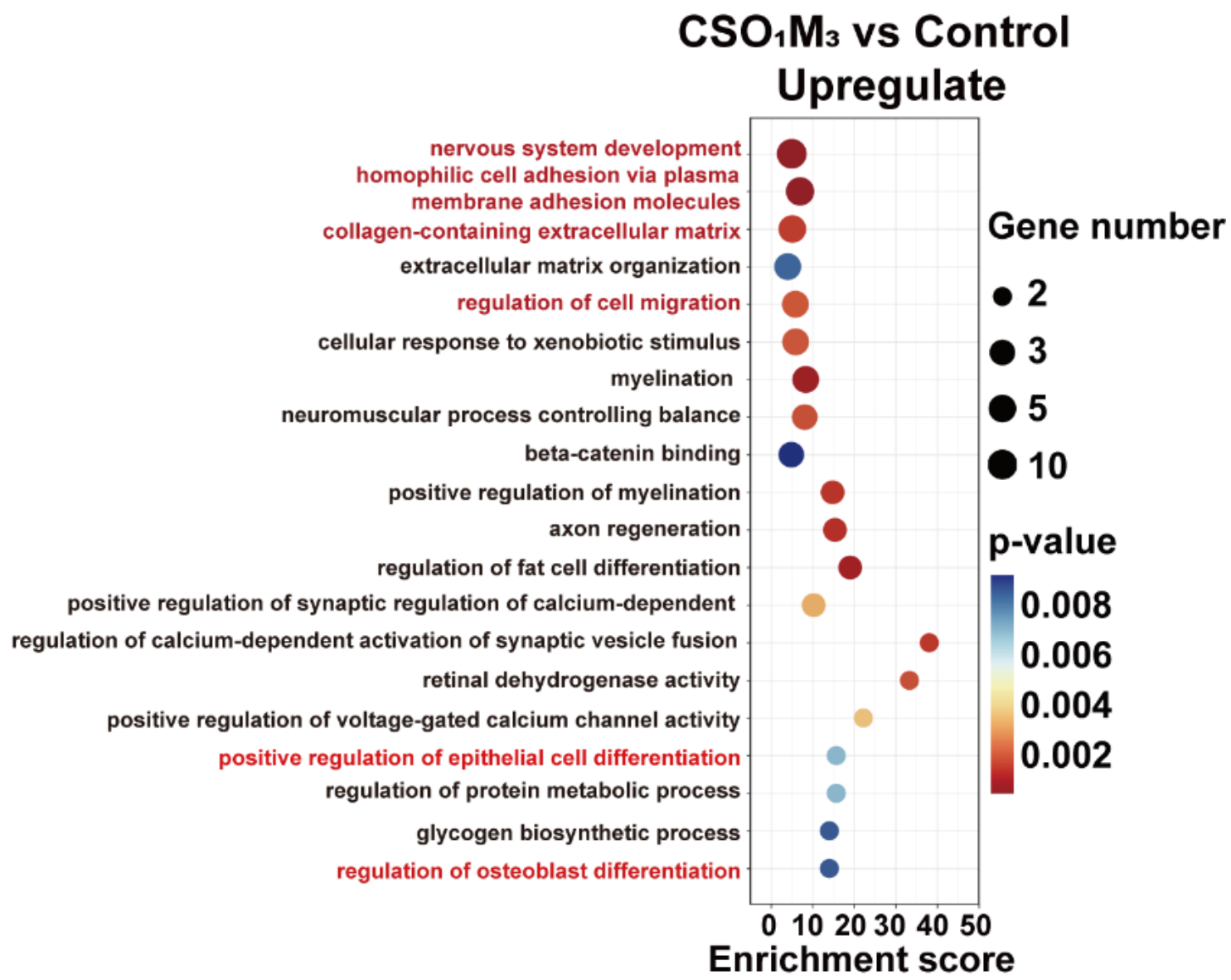

Fig. R17. Transcriptomic comparison between the CSO_1M_3 and control groups at 4 weeks post implantation. GO enrichment analysis of upregulated genes related to various biological responses and functions.

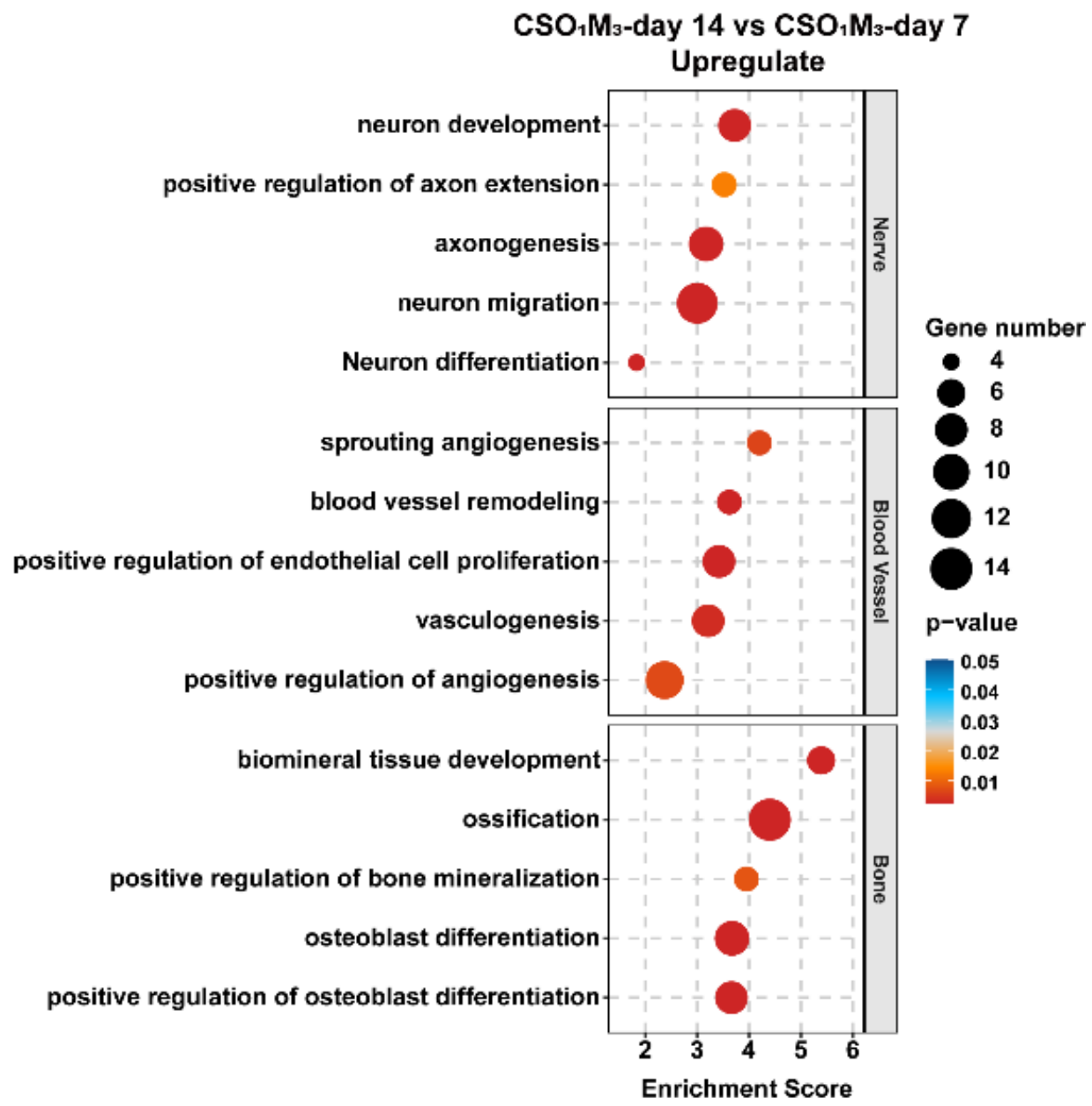

Fig. R18. Single-cell transcriptomic comparison between the CSO_1M_3 groups at 1 week and 2 weeks post implantation. GO enrichment analysis of upregulated genes related to nerve, blood vessel and bone tissues regeneration.

The results and corresponding discussion have also been supplemented in our revised

manuscript (Refer to Page 30 of Revision).

Revision:

[Page 30, Paragraph 1 and 2]

"…In addition, a triple-color immunofluorescence staining results suggested the $TUBB3^{+}$ nerves and $CD31^{+}$ blood vessels were not randomly distributed, but closely surrounded $Osterix^{+}$ newly formed bone tissues in all groups at 4 weeks post-implantation (Supplementary Fig. 45). This specific spatial arrangement indicated these three systems might have a mutually reinforcing relationship. In particular, the CSO_1M_3 membrane induced significantly improved new-tissue formation and coupling intensity in the surrounding region…

Generally, bones were covered by neural and vascular networks essential for the development, remodeling, and repair [58]. Specifically, nerve regeneration regulated osteogenic differentiation and bone metabolism via neuropeptides and cytokines (e.g., CGRP, NGF). The neural pathways could also guide the spatial alignment of newly formed bone tissues. Angiogenesis established a nutrient supply network to supply the basic necessities (e.g. oxygen, nutrients, growth factors) and remove the waste products for the defect region. Blood vessels also played a key role in the recruitment of osteoprogenitor cells. Additionally, nerves and blood vessels interacted synergistically, which nerves promoted angiogenesis via neurotransmitter secretion, and blood vessels in turn supported nerve fiber growth by supplying oxygen and nutrients. Upregulated GO terms including nervous system development, epithelial differentiation, and osteoblast differentiation in Fig. 6j and Supplementary Fig. 46 also confirmed that the CSO_1M_3 group significantly enhanced the synergistic interaction among these new tissues compared to the control group. The CSO_1M_3 membrane was proved to establish a well-integrated spatial structure, highlighting its superior bioactivity for regulating stage-adaptive bone regeneration."

Response to reviewers:

Reviewer #1:

The authors have well addressed my comments.

Response:

We thank the reviewer for accepting our response and giving positive comment.

Reviewer #2:

The authors have largely addressed my concerns.

Response:

We thank the reviewer for accepting our response and giving positive comment.

Reviewer #3:

I have no further question on this manuscript; it can be accepted for publication at its current status.

Response:

We appreciate the reviewer for accepting our response and giving positive comment.
