# Supplementary material for: Author Correction: Unified bilayer membranes with mechanically reinforced interface for stage-adaptive bone regeneration
Source: Nat Commun. 2026 Sep 28;17:10276. doi: 10.1038/s41467-026-78171-y (PMC13619558; doi:10.1038/s41467-026-78171-y)
Supplement: Supplementary file 1 [file 41467_2026_78171_MOESM1_ESM.pdf]

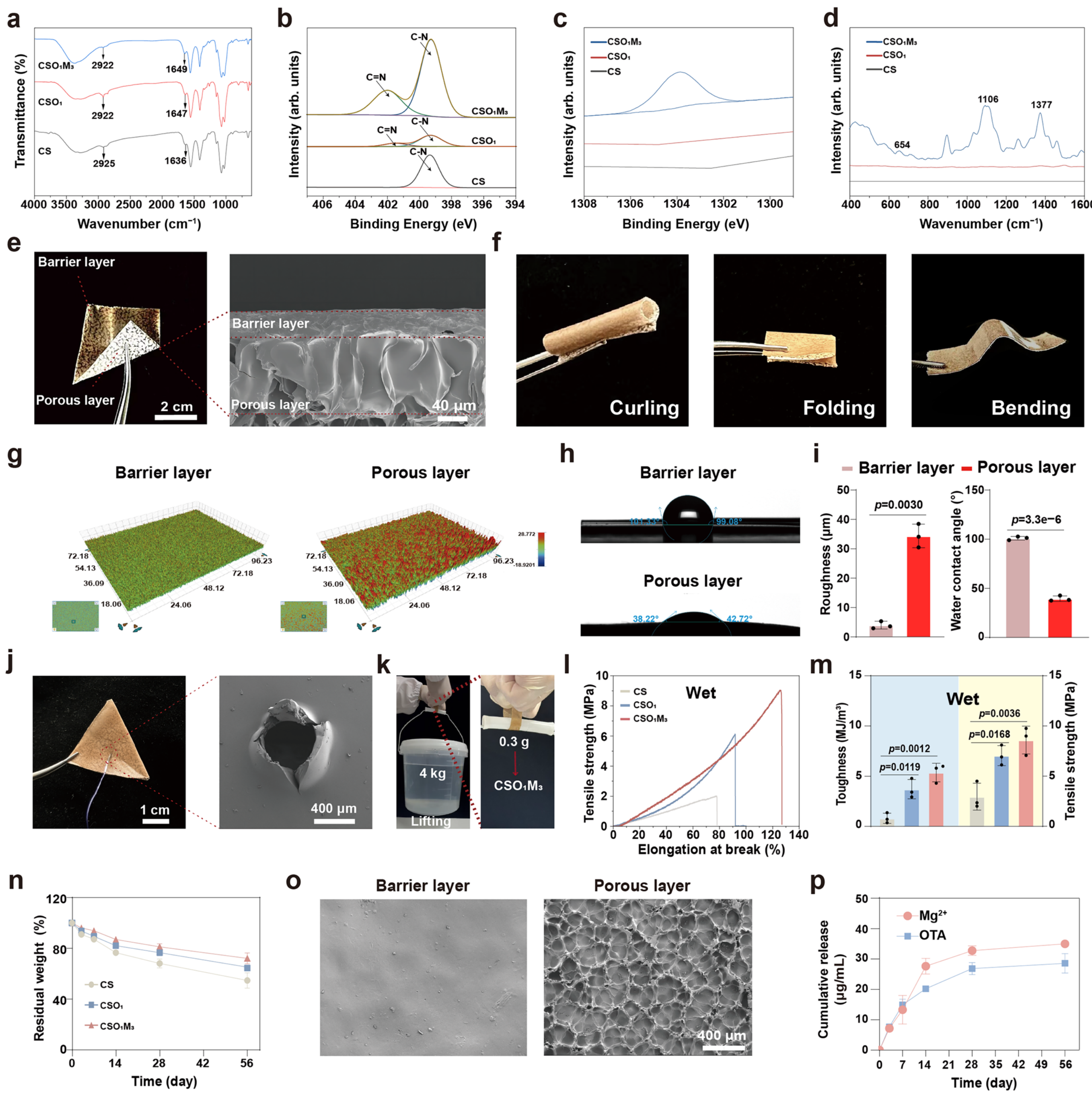

Fig. 2 | Characterization of the unified bilayer membranes. **a** FTIR spectra of various membranes. CS: pristine CS. CSO_1: CS incorporated with 1 wt% OTA. CSO_1M_3: CSO_1 with 3 wt% MgO. **b** Binding energies of nitrogen (N 1*s*) in the XPS spectra of membranes. **c** Binding energies of Mg (Mg 1*s*) in the XPS spectra of membranes. **d** Raman spectra of the membranes. **e** Macroscopic photographs and cross-sectional SEM images of the CSO_1M_3 membranes. The images were repeated at least three times with consistent results. **f** Images of the CSO_1M_3 membranes with various deformations. **g**–**i** Heterogeneous morphologies of barrier layer and porous layer including surface roughness analysis (**g**), water contact angle measurements (**h**) and corresponding data statistics (**i**). **j** Surgical suture puncture tests of the CSO_1M_3 membranes and amplified SEM images of the puncture sites. The images were repeated at least three times with consistent results. **k** Lifting tests with a 4 kg load using the CSO_1M_3 membranes, the red arrow indicates the CSO_1M_3 membranes. **l**, **m** Representative tensile stress-strain curves (**l**) and derived tensile strength and toughness (**m**) of various composite membranes under hydrated conditions. **n** Degradation in vitro of the composite membranes in 56 days. **o** SEM images of the two layers of the CSO_1M_3 membranes after 56-days in vitro degradation. The images were repeated at least three times with consistent results. **p** Cumulative magnesium ions and OTA release curve of the CSO_1M_3 membranes in 56-days in vitro degradation. Values in (**i**, **m**, **n**), and (**p**) represent the mean and standard deviation (n = 3 independent samples per group). Statistical analyses were performed using two-tailed Student's *t*-test (**i**) and one-way ANOVA followed by Tukey's post hoc test (**m**). $p < 0.05$ was considered statistically significant differences between the compared groups. Source data are provided as a Source Data file.
